# The polyphenol oxidase gene family in land plants: Lineage-specific duplication and expansion

**DOI:** 10.1186/1471-2164-13-395

**Published:** 2012-08-16

**Authors:** Lan T Tran, John S Taylor, C Peter Constabel

**Affiliations:** 1 Centre for Forest Biology and Department of Biology, University of Victoria, PO BOX 3020,, Station CSC, Victoria, BC, V8W 3N5, Canada; 2 Department of Biology, University of Victoria, PO BOX 3020,, Station CSC, Victoria, BC, V8W 3N5, Canada

## Abstract

**Background:**

Plant polyphenol oxidases (PPOs) are enzymes that typically use molecular oxygen to oxidize *ortho*-diphenols to *ortho*-quinones. These commonly cause browning reactions following tissue damage, and may be important in plant defense. Some PPOs function as hydroxylases or in cross-linking reactions, but in most plants their physiological roles are not known. To better understand the importance of PPOs in the plant kingdom, we surveyed PPO gene families in 25 sequenced genomes from chlorophytes, bryophytes, lycophytes, and flowering plants. The PPO genes were then analyzed *in silico* for gene structure, phylogenetic relationships, and targeting signals.

**Results:**

Many previously uncharacterized PPO genes were uncovered. The moss, *Physcomitrella patens*, contained 13 PPO genes and *Selaginella moellendorffii* (spike moss) and *Glycine max* (soybean) each had 11 genes. *Populus trichocarpa* (poplar) contained a highly diversified gene family with 11 PPO genes, but several flowering plants had only a single PPO gene. By contrast, no PPO*-*like sequences were identified in several chlorophyte (green algae) genomes or Arabidopsis (*A. lyrata* and *A. thaliana*). We found that many PPOs contained one or two introns often near the 3’ terminus. Furthermore, N-terminal amino acid sequence analysis using ChloroP and TargetP 1.1 predicted that several putative PPOs are synthesized via the secretory pathway, a unique finding as most PPOs are predicted to be chloroplast proteins. Phylogenetic reconstruction of these sequences revealed that large PPO gene repertoires in some species are mostly a consequence of independent bursts of gene duplication, while the lineage leading to Arabidopsis must have lost all PPO genes.

**Conclusion:**

Our survey identified PPOs in gene families of varying sizes in all land plants except in the genus Arabidopsis. While we found variation in intron numbers and positions, overall PPO gene structure is congruent with the phylogenetic relationships based on primary sequence data. The dynamic nature of this gene family differentiates PPO from other oxidative enzymes, and is consistent with a protein important for a diversity of functions relating to environmental adaptation.

## Background

Polyphenol oxidases (PPOs) are dicopper enzymes that oxidize *ortho*-diphenols to *ortho*-diquinones using molecular oxygen. Some PPOs also convert monophenols to *ortho*-diphenols [1]. PPO genes have been identified in green plants as well as in animals and fungi, where they are often referred to as tyrosinases and appear to be involved in pigment formation. The reactive *ortho*-quinone PPO products lead to the familiar browning reactions in damaged fruits and vegetables when exposed to oxygen, for example in freshly sliced apples and potatoes. Thus, preventing PPO-mediated browning reactions is of great importance in the fresh fruit and produce industry as well as for processed food. While the biochemical reactions catalyzed by PPOs are well known, data on physiological functions of the enzyme are scarce. Plant PPOs are often considered to be defense proteins due to their herbivore-, pathogen- and wound-induced expression [2,3]. Furthermore, most PPOs are predicted to be localized in the chloroplast while their phenolic substrates accumulate in the vacuole. Thus, the enzymes can come into contact with its substrate only if cells are disrupted, such as during tissue damage [4]. There is strong evidence for a defensive role of PPO in some plants, for example in tomato and poplar. In other species, the evidence is mixed [1,5].

Expression profiling of PPO transcripts in plants with multiple PPO genes such as tomato and poplar indicates that despite strong stress-induced regulation of some PPO genes, most PPOs are developmentally regulated [6,7]. The diversity of tissues and conditions under which PPO is expressed suggests these enzymes can play roles in a variety of processes [1]. In dandelion (*Taraxacum* spp.), a PPO has recently been implicated in latex coagulation [8], and the hydroxylase activity of some PPO-like proteins suggests they can function in the biosynthesis of phenylpropanoids. For example, aureusidin synthase (AmAS1) and larreatricin hydroxylase (LtLH) are PPOs that are involved in the biosynthesis of aurones and lignans, respectively [9,10]. In the Caryophyllaceae, PPOs function as hydroxylases in betalain biosynthesis [11].

Plant PPO proteins typically consist of three domains: an N-terminal chloroplast transit peptide (cTP), a dicopper centre, and a C-terminal region [12]. The 8–12 kDa bipartite cTP [13], which is usually found at the N-terminus, regulates import into the thylakoid lumen via the twin arginine-dependent translocation (Tat) pathway [14]. However, a signal peptide for the secretory pathway was identified and vacuolar localization subsequently demonstrated in two PPOs, snapdragon (*Antirrhinum majus*) AmAS1, and poplar (*Populus trichocarpa*) PtrPPO13 [7,15]. The dicopper centre consists of two conserved copper-binding domains (CuA and CuB), each with three histidine residues that coordinate a copper ion and comprise the active site [16]. Each copper-binding domain is approximately 50 amino acids in length, separated by a linker segment of approximately 100 residues [12]. Although both domains are conserved and define the PPO protein family, the CuA domain is more variable than the CuB domain and this variation may affect substrate preferences. A C-terminal fragment of the PPO protein in some species is susceptible to proteolytic cleavage, for example in broad bean (*Vicia faba*) and grape berry (*Vitis vinifera*). Cleavage of this domain appears to facilitate activation of latent PPO [17].

The high level of conservation of the PPO Cu-binding domain facilitated the early isolation of PPO cDNAs from a diversity of angiosperms including apple (*Malus domestica*), tomato (*Solanum lycopersicum*) and potato (*S. tuberosum*). Cloning of the respective PPO genes suggested that plants contain multiple, intronless PPO genes. For instance, seven single-exon PPO genes were characterized in tomato [18], and five single-exon PPOs in potato [19]. Subsequent studies from monocots revealed that PPOs can in fact contain introns, for example in pineapple and wheat [20,21]. Little is known about PPO genes in non-economic plants, and no PPO-like genes have been reported from *A. thaliana*. As a result, sequence comparisons do not capture the full diversity of plant PPO gene occurrence and structure. To date, a multi-species analysis of the PPO gene family from sequenced plant genomes has not been conducted [12,22].

Here we take advantage of recent whole genome sequencing projects to test the idea that in green plants, the PPO gene family is highly variable in both gene number and structure. We hypothesized that if there are fewer functional constraints than on other genes, there should be evidence of both expansion and contraction of the gene family. We survey and characterize PPO genes in a diversity of green plants: five green algae, one bryophyte, one lycophyte, five monocotyledonous anthophytes, and 13 eudicotyledonous anthophytes. We hypothesized that comparing these sequences will expose conserved motifs/sub-domains that will facilitate a better understanding of PPO function, as well as delineate the gene duplication events that have generated PPO gene diversity among land plants. A more complete characterization of PPOs may also identify additional genes in economically important species and stimulate future gene silencing efforts. Our results show that the PPO gene family has recently expanded in some species, but is reduced or absent in others. We also discovered that most monocot PPO genes and some eudicot PPO genes contain introns, and that a subset of PPOs are likely not plastidic as previously believed, but are targeted to the secretory pathway. Our work suggests that the evolutionary history of PPOs in plants is complex, and that this likely reflects a diversity of PPO functions.

## Results

### Genomic identification of PPO genes in land plants

Our TBLASTX search uncovered over 130 candidate PPO genes in 18 of the 25 genomes analyzed (Table 1; Additional files 1 and 2), representing four distantly-related lineages of land plants (bryophytes, lycophytes, monocotyledonous anthophytes (monocots) and eudicotyledonous anthophytes (eudicots)). Of these, 107 PPO genes contained no premature stop codons, were at least 1200 bp in length, and encoded proteins with two complete copper-binding regions (Additional file 1). The non-vascular *Physcomitrella patens* (a moss) contained the largest PPO gene family (13 genes) in our survey. The lycopod S*elaginella moellendorffii* had 11 PPO genes, which was unexpected as it has one of the smallest plant genomes known [23]. Among the flowering plants, soybean (*Glycine max*) and monkey flower (*Mimulus guttatus*) have large PPO gene families (11 and 9 members, respectively). The poplar (*Populus trichocarpa*) genome was also found to have 11 PPO genes (with some uncertainty due to annotation ambiguities, see reference [7]), while the genomes of the closely related species cassava (*Manihot esculenta*) and castor bean (*Ricinus communis*) contain only a single PPO gene. Among monocots surveyed, sorghum (*Sorghum bicolor*) has the largest PPO gene family with eight genes, whereas maize (*Zea mays*) and purple false brome (*Brachypodium distachyon*) each contain six PPO genes, and fox millet (*Setaria italica*) has four PPOs. Interestingly, rice (*Oryza sativa*) contains only two PPOs. Despite extensive searches, no PPO genes were detected in the genomes of *A. thaliana* or *A. lyrata*. Though surprising, this result is consistent with an earlier survey of the *A. thaliana* genome, which also failed to identify PPOs [24]. No PPO genes were uncovered in the green algae *Chlamydomonas reinhardtii*, *Micromonas pusilla*, *Ostreococcus lucimarinus*, *O. tauri*, or *Volvox carteri.*

**Table 1:** Number of putative PPO genes identified in available Viridiplantae genomes

**Species**		**Estimated Genome Size (Mb)**^**a**^	**PPO Genes**^**b**^
**Chlorophytes**			
green algae (unicellular)	Chlamydomonas reinhardtii*	120	0
	*Micromonas pullisia*	15	0
	*Ostreococcus lucimarinus*	13	0
	*Ostreococcus tauri* *	12	0
(multicellular)	Volvox carteri*	120	0
**Bryophytes**			
moss	Physcomitrella patens*	500	13
**Lycophytes**			
spike moss	*Selaginella moellendorffii*	100	11
**Monocotyledonous Anthophytes**			
purple false brome	Brachypodium distachyon*	355	6
rice	Oryza sativa*	466	2
foxtail millet	*Setaria italica*	490	4
cereal grass	Sorghum bicolor*	760	8
maize	Zea mays*	2400	6
**Eudicotyledonous Anthophytes**			
blue columbine	*Aquilegia coerulea*	302	7
lyrate rockcress	*Arabidopsis lyrata*	230	0
thale cress	Arabidopsis thaliana*	125	0
papaya	Carica papaya*	372	4
cucumber	Cucumis sativus*	367	1
soybean	Glycine max*	1200	11
cassava	*Manihot esculenta*	760	1
barrel medic	*Medicago truncatula*	500	4
monkey flower	*Mimulus guttatus*	430	9
black poplar	Populus trichocarpa*	480	11
peach	*Prunus persica*	290	4
castor bean	Ricinus communis*	400	1
grapevine	Vitis vinifera*	500	4

^a^ Estimated genome sizes as indicated in NCBI ( http://www.ncbi.nlm.nih.gov/genomeprj).

^b^ Denotes minimum number of PPO genes as identified from this analysis. Additional putative functional PPO gene models with discrepancies were identified for some genomes, but were excluded from this. For *P. trichocarpa*, up to 11 putative functional PPO genes have been identified.

* Indicates genomes described in a publication: *A. thaliana*[25], *B. distachyon*[26], *C. papaya*[27], *C. reinhardtii*[28], *C. sativus*[29], *G. max*[30], *O*. *sativa*[31], *O. tauri*[32], *P. patens*[33], *P. trichocarpa*[34], *R. communis*[35], *S. bicolor*[36], *V. vinifera*[37], *V. carteri*[38] and *Z. mays*[39].

Since several sequences uncovered by our TBLASTX search were either incomplete or had annotation discrepancies (Additional file 2), the numbers of PPO genes reported here are likely to be minimums. For example, the soybean gene sequence Glyma07g31290.1 predicts a five-exon gene that would encode an excessively large protein of 1000 amino acid residues that would be much larger than typical PPOs. Multiple sequence alignments of this gene and characterized PPOs indicated that the predicted gene structure is likely not correct. Specifically, if only exons I, II, IV, and V are considered, this gene could encode a 615 amino acid polypeptide comparable in size to the other soybean PPOs. Other putative sequencing errors were detected in the *Mimulus* gene mgf021284m, which appears to be annotated with an incorrect ATG initiation codon that would produce a protein larger than expected from its paralogs. However, since we did not independently verify them, these and other problematic sequences were not used for further analyses.

### Functional domains of PPOs are conserved

**Figure 1:**
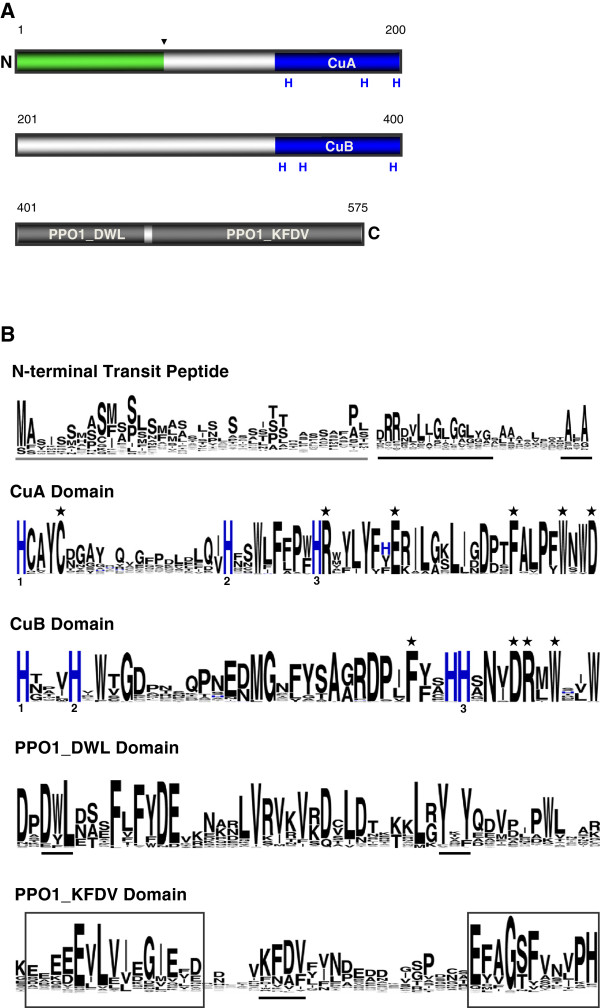
**Schematic diagram of PPO domains and conserved residues.** (**A**) Typical PPOs contain an N-terminal transit peptide (green), which is cleaved at an alanine motif (inverted triangle) after import into the thylakoid lumen. The conserved CuA and CuB domains are shown in blue, the C-terminal domains in grey. (**B**) WebLogo sequence logos indicating conserved residues in PPO domains. The first 35 amino acids of the transit peptide are shown (underlined in grey). The thylakoid transfer domain, the alanine (AxA) cleavage motif, the DWL motif, the tyrosine (YxY) motif and the KFDV motif are underlined in black. The three conserved histidine residues in both the CuA and CuB domains are numbered and shown in blue. Black stars indicate absolutely conserved residues. The boxed sequences in the PPO1_KFDV domain are conserved regions identified in this study.

PPO proteins generally contain three conserved regions: an N-terminal cTP, a CuA and CuB (tyrosinase) domain and a C-terminus extension (Figure 1a). Sequence logos for each of these regions were generated using WebLogo [40], which identified highly conserved amino acid residues (Figure 1b). In the first 35 residues of the predicted PPO protein, we observed a high proportion of serine residues, typical of the stromal peptide of the cTP. Adjacent to this sequence, a thylakoid transfer domain (TTD) and an alanine cleavage motif (AxA) were often evident. Together, these features suggest that most PPO proteins are transported to the thylakoid lumen in the chloroplast. For approximately 75% of these PPOs, a plastidic localization domain was detected by ChloroP 1.1 (Additional file 1) [41]. 

Surprisingly, PPO genes in *P. patens* and a small number of flowering plants did not contain a cTP. Rather, these PPOs appeared to have an N-terminal signal peptide and are predicted by TargetP 1.1 to be synthesized via the secretory pathway [42]. Examples of predicted non-plastidic PPOs are found in both monocot and eudicot groups, including rice, maize, and columbine (*A. coerulea*) (Additional file 1). Experimental proof of a non-plastidic localization for a PPO protein has so far only been achieved for AmAS1 from snapdragon and PtrPPO13 from poplar [7,15], both of which localize to the vacuole.

The Cu-binding domains are characterized by several conserved histidine residues. In the CuA domain, the first of these occurs at the beginning of a HXXXC motif [16] and is most commonly HCAYC (Figure 1b). The second Cys in this motif is predicted to form a thioether bond with the second conserved histidine of the CuA domain. Some PPOs, however, contained rarer motif variants such as HEAYC or HQSYC. Between this HXXXC motif and the second conserved histidine, the sequence is highly variable in both number and type of residue. Other highly conserved residues in the CuA domain were arginine, glutamic acid, phenylalanine, tryptophan and aspartic acid, located downstream from the third conserved histidine. In the CuB domain, we found the first two conserved histidine residues to be within in a previously unidentified HxxxH sequence motif (Figure 1b). At the fourth position in the motif, a hydrophobic residue, either alanine, valine, leucine, isoleucine or methionine, was usually present. C-terminal from the second conserved histidine within the CuB domain, a phenylalanine residue was 100% conserved (Figure 1b).

The C-terminal end of PPO consists of a 50 amino acid PPO1_DWL domain (Pfam12142), and a 140–150 amino acid PPO1_KFDV domain (Pfam12143) (Figure 1b). The functional significance of these domains is not known, but in those PPOs where proteolytic processing of the C-terminus has been documented the cleavage occurs in the PPO1_DWL domain immediately C-terminal to the tyrosine (YxY) motif [22]. As a result of this processing, a polypeptide fragment of approximately 16–18 kDa containing the PPO1_KFDV domain is lost [17]. Our analysis identified two visible sequence motifs within this domain, which were also recently noted [43]. The first motif (EEEEEVLVI) is enriched in glutamic acid residues and is present in most of the land plant PPOs (Figure 1b). C-terminal to this sequence motif is the KFDV motif, also present in many anthophyte PPO sequences and three *Selaginella* PPOs (*SmoPPO1*, *SmoPPO2*, and *SmoPPO3*). In addition, an EFAGSF motif is present in many PPOs. In some sequences, immediately C-terminal to the histidine at the end of the EFAGSF motif, are up to four additional histidines residues that have been hypothesized to form a third copper-binding domain [44]. The functional importance of all these motifs still needs to be determined, however.

### Phylogenetic analysis reveals many species-specific PPO clades

**Figure 2:**
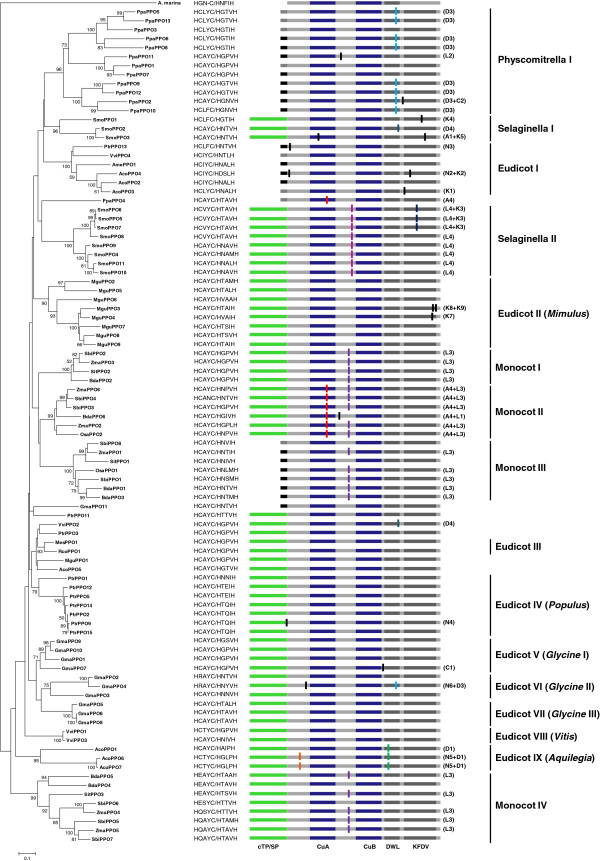
**Neighbour-joining phylogenetic tree from four major land plant lineages, together with corresponding visual representation of conserved regions, functional motifs, and relative intron positions.** A putative tyrosinase sequence from the cyanobacterium *A. marina* (GenBank accession ACJ76786) was used to root the tree. Bootstrap replicates (1000) were used to determine the level of support at each node (only values > 50% are shown). The conserved first five amino acids for each of the CuA and CuB domains is shown at the end of each branch as HxxxC / HxxxH. Predicted targeting sequences are colored as green (chloroplast transit peptide), black (signal peptide), or grey (unknown). The CuA and CuB domains are colored blue, and C-terminal conserved areas dark grey. Approximate intron positions are shown as vertical bars, mapped onto the predicted protein. Shared colors indicating the same intron positions, and black bars mark unique introns. The introns are named by their location: N, N-terminus; A, CuA domain; L, linker; D, DWL domain; K, KFDV domain; C, C-terminus. Exact intron positions are listed in Additional file 4. The PPO sequences are numbered and named based on species names as follows: *P. patens*, Ppa; *S. moellendorffii* , Smo; *B. distachyon*, Bda; *O. sativa*, Osa; *S. italica*, Sit; *S. bicolor*, Sbi; *Z. mays*, Zma; *A. coerulea*, Aco; *G. max*, Gma; *M. esculenta*, Mes; *M. guttatus,* Mgu; *P. trichocarpa*, Ptr; *R. communis*, Rco; *V. vinifera*, Vvi. Mexican poppy (*Argenome mexicana*) *AmePPO1* (GenBank accession ACJ76786) was also included in the phylogeny because of our interest in the Eudicot I clade.

A neighbour-joining phylogenetic reconstruction was generated from a multiple sequence alignment of the copper-binding domains and the PPO1_DWL domain of PPO protein sequences from 14 of the 25 plant genomes we had surveyed (Additional file 3). The genomes were chosen to be representative and to cover a broad range of plant lineages. The analysis separated PPOs into a number of distinct clades (Figure 2). While the nodes at the base of the larger clades were not well supported (low scores in the bootstrap reanalyses), nodes at the base of many smaller clades were robust (bootstrap values > 70%). In *Physcomitrella, Selaginella,* and in the eudicots, PPO diversification is largely a consequence of species-specific gene duplication and divergence. Thus, 12 of the 13 *Physcomitrella* sequences occur in one group, and eight of the eleven *Selaginella* sequences form one clade, with the remaining three genes forming a second clade. Among the eudicots, 10 of the 11 *Glycine* PPOs form a monophyletic group, seven of the 10 *Populus* PPOs form a monophyletic group, and all but one of the nine *Mimulus* PPOs occur in a single clade. While these data show that PPO gene diversification has occurred independently in different eudicots, we note that these species also have one or two PPOs on separate branches, sometimes in well-supported clades with other eudicot genes. This indicates that the common ancestor of the eudicot lineage had several PPO genes.

This pattern is exemplified by the *Populus* PPO gene family. As seen in Figure 2, seven *Populus* genes form the Eudicot IV clade. However, *Populus PtrPPO3* is in a group with orthologs from *V. vinifera, M. esculenta, R. communis*, *M. guttatus* and *A. coerulea*. Likewise *Populus PtrPPO11* occurs in a separate group together with a *Glycine* PPO gene (*GmaPPO11*). Finally, *Populus PtrPPO13* occurs in a well-supported, multispecies clade with orthologs from diverse angiosperm lineages including *V. vinifera*, *Argemone mexicana* and *A. coerulea* (Eudicot I). The genes in this clade are distinct in that they encode proteins that possess signal peptides rather than cTPs. Together, these observations suggest that, depending upon the correct position of *PtrPPO11*, there were three or four PPO genes in the common ancestor of the eudicots in our survey.

By contrast, monocot PPO diversification appears to have occurred prior to the divergence of the lineages included in our survey (Figure 2). Indeed, in only two instances are paralogs also sister genes on the tree (*BdaPPO1/BdaPPO3* and *SbiPPO3/SbiPPO4*). In all other cases, duplication events occurred prior to the divergence of at least two of the species in our analysis. Interestingly, it appears that the ancestor of all monocots in our survey had four PPO genes, much like the eudicot ancestor. Some of these PPOs appear to have been lost in rice, as Monocot clades I and IV do not contain PPOs from rice. Clade Monocot III is distinct in that its members all have signal peptides and five of seven contain introns, both also features of the Eudicot I clade.

### Introns are common features of PPO genes

Early studies detected introns in PPO genes from monocots [20,21,45], but not eudicot species. The discovery of introns in cherimoya (*Annona cherimola*) *AcPPO* and poplar *PtrPPO13* genes provided the first exceptions to this pattern [7,46]. The current analysis predicted introns in 58 of the 107 PPO-encoding genes (Figure 2, Additional file 4), and suggests a broad distribution of introns in PPO genes from several plant lineages. Introns were also identified in a number of eudicot lineages, but were much less common in this group.

Mapping the pattern of intron distribution and position onto the phylogeny revealed both shared and unique introns. For example, it seems most likely that the PPO gene that gave rise to the large clade of *Physcomitrella* genes possessed an intron (D3). Retroduplication, generating an intronless gene, appears to have occurred at the base of a three-gene clade (*PpaPPO1, PpaPPO7,* and *PpaPPO11*). Similarly, *PpaPPO3* also lacks introns. In addition to these two-intron loss events, *PpaPPO2* and *PpaPPO11* appear to have gained introns independently. In the largest *Selaginella* clade (eight genes), all of the PPO genes share intron L4 and three of these genes share a second intron (K3). As mentioned above, the remaining three *Selaginella* PPO genes (*SmoPPO1, SmoPPO2,* and *SmoPPO3*) form a monophyletic group, but each gene appears to have gained one or two introns independently.

Our phylogenetic analysis indicates that intron L3 was present in the common ancestor of all monocot PPO genes in our study. This intron appears to have been lost independently in two *B. distachyon* genes (*BdaPPO4* and *BdaPPO6*), in three *S. bicolor* genes (*SbiPPO6*, *SbiPPO7*, and *SbiPPO8*) and in *SitPPO1* from *S. italica*. Our tree also shows the gain of a second intron (A4) at the base of monocot clade II, which contains PPOs from *S. bicolor, Z. mays, B. distachyon*, and *O. sativa*. Introns were also identified in the eudicot gene surveyed, but were much less common in this group (Figure 2, Additional file 4). Of the eudicots, *A. coerulea* had the most intron-containing PPO genes. For example, *AcoPPO6* and *AcoPPO7* shared introns N5 and D1, while *AcoPPO1* has lost intron N5 but retained intron D1. In most of the other eudicot genomes, PPO introns were not common and often unique, suggesting these were gained recently.

The position of the introns within the PPO coding sequence showed a non-random distribution. Introns were most common within the linker that separates the CuA and CuB domains, and within the PPO_DWL domain (Figure 2, Additional file 4). Only rarely was an intron predicted within a functional domain. For example, in the Monocot II clade the CuA domain is interrupted by intron A4 immediately after the third conserved histidine residue in the FFPWH motif. In some cases, introns at other positions were predicted, such as the 164 bp intron at the 5' terminus of the poplar *PtrPPO13* gene. Interestingly, this is similar in position to the intron in the *A. cherimola* PPO gene [46]. Intron lengths in the PPO genes ranged from 39 to 2203 nucleotides. In *Physcomitrella* and *Selaginella* they ranged from 45 to 988 nucleotides, while in monocots, PPO introns were 50 to 2203 bp in length. Inspection of the predicted introns identified a 5’ GT-AG 3’ terminal dinucleotide consensus sequence in all but one of the intron-containing PPO genes, typical of eukaryotic U2-type introns.

## Discussion

In our survey of 25 genomes from different lineages in the plant kingdom, many previously uncharacterised PPO genes were identified. We found substantial diversity among species in PPO gene number, with many examples of lineage-specific gene family expansion and gene loss. Exon-intron structure also varied. Intron gain and loss, likely as a result of retroduplication, were common.

### Variable numbers of PPO genes are present in all land plants surveyed but absent in Arabidopsis

The largest number of PPO genes was identified in the moss *P. patens* (Table 1), of which only one had been previously described [47]. In *Selaginella*, an early tracheophyte with a very small genome [23], we also discovered an extensive PPO gene family. The presence of PPO enzyme activity was previously reported in other non-vascular plants including *Marchantia polymorpha*[48]. By contrast, we found no evidence of PPO-like genes in unicellular green algae (*C. reinhardtii*, *M. pusilla*, *O. lucimarinus* and *O. tauri*), or in the multicellular alga *Volvox carteri* (Table 1). The current genomics resources thus suggest that PPO genes became important concurrently with the emergence of land plants. Interestingly, class III peroxidases and laccase genes, which encode enzymes that carry out reactions similar to PPO, are also numerous in *P. patens*, and *S. moellendorfii* (Table 2) [49,50]*.* Thus it is possible that oxidative enzymes including PPO only became important in plants when they successfully colonized land. The PPO family differs from the laccase and class III peroxidase families in that it did not expand with the diversification of land and flowering plants, but was either maintained or reduced, and in the case of Arabidopsis, eliminated completely. Thus, PPOs seem to be more variable in number than similar oxidases, perhaps a reflection of different functions (see below). 

**Table 2:** Sizes of gene families encoding oxidative enzymes from selected plant genomes

	***P. trichocarpa***	***O. sativa***	***A. thaliana***	***S. moellendorffii***	***P. patens***	***C. reinhardtii***
**PPO**	**11**	**2**	**0**	**11**	**13**	**0**
laccase^a^	39	20	17	10	12	3
Class III peroxidase^a^	105	138	73	79	43	0

^a^ Gene family data from [49].

Surprisingly, no PPO sequences have been characterized from gymnosperms. However, we recently identified ESTs that encode fragments of PPO enzymes from *Picea sitchensis* and *Cryptomeria japonica* (unpublished data). This confirmed that PPO genes are indeed found in gymnosperms, although their low prevalence in EST databases suggests they are not widely expressed. Because these ESTs only encoded PPO fragments, however, we were not able to analyze them further.

Despite exhaustive searches, no PPO genes could be identified in *A. thaliana* or its close relative *A. lyrata*. Likewise, we found no evidence of PPO genes in the closely related *Brassica napus* after searching the *Brassica* BRAD EST database [51] and Genbank. Based on the presence of a PPO gene in papaya, we assume that the common ancestor of Brassicales and Malvales must have contained a PPO gene, which was lost from the ancestor of Arabidopsis and its relatives after the divergence of these sister groups. The lack of a PPO gene in Arabidopsis suggests that PPOs are likely not required for a primary metabolic function. Rather, this finding points to ecological or secondary metabolic functions for PPOs (see below). Alternatively, there may be functional redundancy and that the lack of PPO genes in Arabidopsis could be compensated by other oxidative enzymes such as laccases. Although structurally not related, laccases and PPOs carry out similar phenolic oxidations using molecular oxygen [52]. The Arabidopsis genome contains 17 laccase genes (Table 2); however, none of these contain a chloroplast TP, suggesting they are unlikely to easily compensate for the lack of PPO.

The monocots typically contained two to eight PPO genes (Table 1), but in eudicots gene numbers ranged from zero to eleven. For example, poplar has one of the larger PPO gene families with up to 11 genes in several clades. One of these is the result of extensive duplication, leading to a clade of six closely related genes within the *Populus PtrPPO2* subgroup. By contrast, castor bean (*R. communis*), despite being closely related to poplar, has only a single PPO (Table 1, Figure 2). The variable number of PPO genes in different species is intriguing, in particular because this variability is not seen in other oxidative enzymes and suggests PPO family expansion is driven by clade-specific ecological or metabolic selection pressures. It is tempting to speculate that PPO genes duplicated in those lineages with complex phenolic-based secondary metabolism. This might be the case in *P. patens*, where other genes associated with the phenylpropanoid pathway are also overrepresented, and 17 putative chalcone synthase (CHS) genes have been identified [53]. Soybean and poplar, species with large PPO gene families, belong to taxa known for their abundant and diverse phenolics and flavonoids [54,55]. Both are known to contain high levels of PPO activity [3]. Among cereal grains (monocots), sorghum has the largest PPO family, and also has high levels of phenolics compared to other monocots [56].

### Phylogenetic analysis reveals lineage-specific expansion of the PPO gene family

It is evident from the phylogenetic reconstruction that there are several well-supported PPO clades (> 70% bootstrap support), which are generally congruent with the conserved intron positions (Figure 2). This pattern is most evident for the monocot PPOs. Here, a common ancestor of the modern grasses likely had at least three PPO genes, which are retained in the major cereals today. Independent support for the Monocot II and Monocot IV clades comes from a more detailed analysis of PPOs in barley, where one clade consisting of the two-intron PPO genes and a second clade with the signal peptide-encoding PPO genes was recently described [45].

The structure of the eudicot PPO clades is not as clear or consistent as for the monocots, as low bootstrap values obscure the exact relationships. In the eudicots, there are several clades where gene duplications have clearly contributed to the expansion of PPO gene families within a lineage. The poplar, soybean, and monkey flower show large PPO gene families, which may have been generated by tandem gene duplication. Although we did not specifically examine the physical location of PPO genes on chromosomes, a tandem arrangement can be inferred in a few cases. Inspection of chromosomal localization of PPOs in the soybean genome suggests that at least some PPO genes are in close proximity (Additional file 1). In soybean, nearly three quarters of genes are known to exist as duplicate or multiple copies, with some arranged in tandem [30]. In poplar, where the whole genome has undergone a recent duplication [34], the *PtrPPO2* subgroup has expanded substantially through additional gene duplications. Although the chromosomal location and exact number of *PtrPPO2* subgroup genes has been difficult to resolve [7], we predict that these genes are arranged in close proximity to each other. Tandem duplications are also likely in the tomato PPO gene family, in which all seven PPO genes were mapped to chromosome eight [18]. Similarly, a cluster of PPOs was described for red clover [57]. Interestingly, a recent genome-wide study comparing orthologous groups of genes in four model genomes (Arabidopsis, poplar, rice, and *P. patens*) found that genes with stress-responsive expression patterns (including defense) are more likely to have undergone lineage-specific tandem duplication than genes involved in primary metabolic and cellular functions [58]. Tandem gene arrangements would therefore be consistent with functions of PPO related to stress and ecological adaptation.

### Both conserved and unique introns suggest PPO is a dynamic gene family

PPO genes were originally thought to lack introns, as the first PPO genes to be cloned were from eudicots [18]. Our study confirmed that eudicot PPOs typically have no introns, with the Eudicot I and IX clades being marked exceptions to this trend. By contrast, our work identified a large number of intron-containing PPOs in monocot, *Physcomitrella*, and *Selaginella* groups (Figure 2). Primary sequences and gene structures were usually consistent, and genes in well-supported monophyletic clades tended to have the same intron structure. This is most evident in the highly conserved PPO gene structures from monocots. Most PPO genes in monocot clades I-IV, for example, contained intron L3, while genes in Monocot clade II also contain intron A4 (Figure 2). Most of the PPO genes in the well-supported *Physcomitrella* I clade share an intron, though both gain and loss events were observed in this clade. Evolutionary relationships among PPO genes in *Selaginella* clade II were perfectly correlated with exon-intron structure. In the smaller clade (*Selaginella* I), we observed several independent intron gain events. Within the eudicots, the distribution of introns on the tree suggests that most were generated recently and that the ancestral PPO was a single-exon gene.

The observation that unlike the other groups, most eudicot PPO genes have no introns, could suggest that the eudicot genes are monophyletic and are descendants of a gene that was retroduplicated in the eudicot ancestor. This would imply multiple independent and unique intron insertions among the 44 eudicot PPO genes in our analysis. Under the assumption that the eudicot, monocot, *Selaginella* and *Physcomitrella* PPO genes each form monophyletic groups (a hypothesis neither strongly supported nor refuted by our phylogenetic analysis), two major patterns of intron gain or loss are equally parsimonious. If the ancestral PPO was a single exon gene, then intron D3 was gained at the base of the *Physcomitrella* PPO clade, intron L4 was gained at the base of the *Selaginella* PPO clade, and intron L3 was gained at the base of the monocot PPO clade. This pattern also infers a number of intron losses across these three groups, and several intron gains within the eudicots. In this scenario, the absence of introns in the three eudicot clades is a shared ancestral trait. The other, equally parsimonious hypothesis is that the ancestor of plant PPO genes had intron D3, which is still present in most *Physcomitrella* genes, but lost in the ancestor of all non-moss PPOs. This hypothesis also implies the gain of intron L4 in the PPO that gave rise to *Selaginella* paralogs, the gain of intron L3 at the base of the monocot PPO clade, as well as numerous other intron gains and losses in individual genes of the smaller clades.

Regardless of the structure of the ancestral gene that gave rise to all of the genes in our survey, PPO gene structure of extant plants varies enormously. Multiple intron gain and loss events are inferred by our tree. Some of these events are very old, i.e. gains or losses at the base of each of these major taxonomic lineages, and some are recent, occurring in only one gene. Such a dynamic pattern is consistent with our phylogeny, where gene duplication has given rise to expanded PPO gene families in some lineages but not others.

### PPOs as adaptive proteins for a diversity of ecological functions

The features of the PPO gene family including variation in gene number, cellular localization, and lineage-specific diversification is consistent with the idea of PPOs as flexible enzymes that evolution has adapted to a variety of specific functions. Our data show that the PPO gene family is dynamic and greatly expanded in some lineages, but reduced in others. This pattern is reminiscent of the distribution of secondary plant metabolites, which is also very much lineage-dependent, varies tremendously among plant taxa, and appears to be the result of gene duplication and diversification [59]. Secondary metabolites are known as important mediators of ecological interactions and environmental adaptation, and we speculate that the variable expansion of the PPO gene family also reflects such an adaptive function.

Their broad substrate specificity and ability to oxidize a variety of *ortho*-diphenolic compounds make PPOs flexible enzymes which could play diverse physiological roles. The reaction products, the *ortho*-quinones, are reactive chemicals which are often important in situations requiring rapid cross-linking. Two well-documented examples are the PPO-mediated latex coagulation in *Taraxacum* species [18], and the entrapment of aphids by PPO-containing glandular trichomes in tomato and potato [60]. One frequently discussed function of PPO is as an induced herbivore defense against leaf-chewing insects, and the effectiveness of PPO has been demonstrated convincingly in tomato [61]. Herbivore-inducible PPO genes are known from a number of plants [1]. However, these inducible PPO genes do not cluster together in phylogenetic trees [7] and were thus likely recruited for defense independently. A dispersed distribution was also seen for PPOs that function in hydroxylation reactions, such as aureusidin synthase and larreatricin hydroxylase [9,10], which both group with different PPO clades [7]. Therefore, it appears that similar physiological functions for PPO have evolved repeatedly in different lineages.

A rapid mechanism for the evolution of novel functions could be targeting of PPOs to new compartments within the cell. The plastidic location of most PPOs is well established but perplexing because the phenolic substrates are typically stored in the vacuole. Our discovery of several well-supported clades of PPO genes with predicted signal peptides is a potential clue. The Eudicot I clade has representatives from several flowering plants, including the vacuole-localized PtrPPO13 [7], and we speculate that like the *AmAS1* gene product [9], it could also have a biosynthetic function. It will be interesting to determine the cellular localization of the other non-plastidic PPOs identified here as a first step towards discovering additional roles for PPO in plants.

## Conclusions

Our survey of PPO genes in sequenced green plant genomes uncovered significant diversity in PPO gene family size as well as gene structure. This diversity reflects the pattern of lineage-specific gene family expansion, as well as gene loss, revealed by phylogenetic analysis. The dynamic nature of the gene family is consistent with diverse potential roles of PPOs in ecological adaptation.

## Methods

Three PPO genes from hybrid poplar (*P. trichocarpa* x *P. deltoides*), *PtdPPO1*, *PtdPPO2* and *PtdPPO3* (GenBank accessions AF263611, AY665681 and AY665682) were used to search for PPO homologs among the gene predictions from 25 green plant genomes (masked) available at the United States Department of Energy Joint Genome Institute ( http://www.jgi.doe.gov/) (Table 1). TBLASTX searches were conducted using default parameters. BLAST hits returned were translated, manually checked, and analyzed using a combination of NCBI BLASTP and SMART (Simple Modular Architecture Research Tool, http://smart.embl-heidelberg.de/) to confirm the presence of both conserved CuA and CuB domains (tyrosinase domain, Pfam00264). Sequences lacking all three essential histidine residues in both domains [16] were eliminated, as were truncated gene models (shorter than 1200 bp), or models with premature stop codons or other annotation discrepancies. Altogether, 107 putative full-length or near full-length PPO sequences were retained for further analysis. N-terminal transit peptide sequences were predicted using ChloroP 1.1 and TargetP 1.1 [41,42]. Gene models were inspected for annotation of introns, and exon-intron boundaries manually checked. For a subset of genes, predictions pertaining to the types of introns were independently checked using CIWOG (Common Introns Within Orthologous Genes, http://ciwog.gdcb.iastate.edu/) [62].

PPO multiple sequence alignments were generated using MUSCLE (Multiple Sequence Comparison by Log Expectation) [63] with default parameters ( http://www.ebi.ac.uk/Tools/muscle/index.html). Alignments confirmed the positions of the conserved histidine residues in both the CuA and CuB domains. For multiple sequence alignments the N- and C-termini were removed, leaving the core PPO protein containing the CuA and CuB domains, and the PPO1_DWL domain (Additional file 3). Other alignment manipulations were completed in BioEdit. We used WebLogo [40] to help visualize sequence conservation in these domains.

A neighbour-joining phylogenetic tree based on the alignment described above was generated using MEGA 4.0 [64]. Genetic distances were estimated using the Dayhoff amino acid substitution matrix. Positions in the alignment lacking amino acid residues were excluded from the pairwise distance estimates. Bootstrap replicates (1000) were used to indicate the level of support from the data for each node of the tree. A putative polyphenol oxidase (tyrosinase) from the cyanobacterium *Acaryochloris marina* (GenBank accession YP_001521388) was used as the outgroup for the tree.

## Abbreviations

PPO: Polyphenol oxidase; cTP: Chloroplast transit peptide.

## Competing interests

The authors declare that they have no competing interests.

## Authors’ contributions

LTT carried out the bioinformatic and genomic analyses and drafted the manuscript. JST participated in the design of the study, interpretation of results, and writing of the manuscript. CPC contributed to study design, data analysis and writing of the manuscript. All authors read and approved the final manuscript.

## Supplementary Material

Additional file 1 **PPO gene models identified using BLAST searches of selected land plant genomes and validated as described under Materials and Methods.** Table with additional information on validated PPO gene models identified in this study. Click here for file

Additional file 2 **Potential PPO gene models identified using BLAST searches but rejected due to sequence inconsistencies.** Table with additional information on rejected PPO gene models. Click here for file

Additional file 3 Alignment. Figure showing amino acid alignment used to generate the PPO phylogeny. Click here for file

Additional file 4 **Intron/exon gene structures identified in PPO genes.** Table of additional information regarding intron position and structure in PPO genes. Click here for file
