# Supplementary material for: The polyphenol oxidase gene family in land plants: Lineage-specific duplication and expansion
Source: BMC Genomics. 2012 Aug 16;13:395. doi: 10.1186/1471-2164-13-395 (PMC3472199; doi:10.1186/1471-2164-13-395)
Supplement: Additional file 3 — Alignment. Figure showing amino acid alignment used to generate the PPO phylogeny. [file 1471-2164-13-395-S3.pdf]

```
                  10         20         30         40         50         60         70         80         90        100
                  ....|....| ....|....| ....|....| ....|....| ....|....| ....|....| ....|....| ....|....| ....|....| ....|....|
BdaPPO1     ---------- ---------- ---------- --------MA MSTRCHAPLA ACVFLV---- CAVS-MAVYT A--------- ------FP-L SMSPCTNSLS
BdaPPO3     ---------- ---------- ---------- ---------M ASSRC-GPLG ACVLLI---- CAVA-TAVYT A--------- ------FP-V SVNPCTYSLP
SbiPPO1     ---------- ---------- ---------- ---------- -MAGSSSRNG GLLTLRIILL CAFA-AAAIT L--------- ------LP-L VMDPCAHSLS
OsaPPO1     ---------- ---------- ---------- ---------- -----MANER CRVALFVLIV CAFAYAAVYT S--------- ------SPAV SVNPCAQTLT
ZmaPPO1     ---------- ---------- ---------- ---------- ---MAGSNGT LLLALRIVLF CALA-SVATT L--------- ------LP-L VTRPCAYSLP
SitPPO1     ---------- ---------- ---------- ---------- -----MAGSN GALLAFRILL CCALAFTVTV L--------- ------LP-L ALRTCAYSLS
SbiPPO8     ---------- ---------- -----MPESF WREATKCILR VWAMAGSNGT LLIAFRIVLF CALASAATTL L--------- ------LP-L ITRPCASSLS
ZmaPPO2     ---------- ---------- ---------- -----MRSLS LSLSLYIYHH TSIPAPCTPQ CRQDREAYMD STNVASAAPR PRAPCSLQAA LVRRAAVVRT
OsaPPO2     ---------- ---------- ---------- ---------- --------ME SINVAPGTTA TPRMAPPPPP P--------- ------CITN LQSTLRYNNL
BdaPPO6     ---------- ---------- ---------- ---------- ---------M EISTGTPRFT SYTLLTPRSR I--------- ---------P YCIRLRSLLH
SbiPPO3     ---------- ---------- ---------- ---------- -------MAR HRSQLT---- SSAMGSATMP R--------- ---------- -------SFA
ZmaPPO6     ---------- ---------- ---------- ---------- ---------- ---------- -----MLIAT T--------- ------MP-- -------RGF
SbiPPO4     ---------- ---------- ---------- ---------- ---------- ---------- MLSATMPRGF A--------- ---------- ----------
MguPPO1     ---------- ---------- ---------- ---------- -------MAC SIKNTP---- HIATTGAGGA A--------- ------SSPL LV-----KPT
BdaPPO2     ---------- ---------- ---------- ---------- ---------- ---------- MASL-SQLIA R--------- ------PTTT VQCYPWSPCS
SitPPO2     ---------- ---------- ---------- ---------- ---------M ASMSQL---- IARPATFSLS S--------- ---------- -------PRT
SbiPPO2     ---------- ---------- ---------- ---------- ---------- -MASMS---- HLIAKPAPAA T--------- ------FP-L SS-----PRT
ZmaPPO3     ---------- ---------- ---------- ---------- ---------- -MASMS---- HLIAKPAPAA T--------- ------FP-L SL-----PRT
AcoPPO6     ---------- ---------- ---------- ---------- -------MSL SLLATS---- TVPS-SSLLN V--------- ------NP-L RS-----NTK
AcoPPO7     ---------- ---------- ---------- ---------- ---------M SLSLLA---- TSTVPSSSLL N--------- ------VNPL RS-----NTK
AcoPPO1     ---------- ---------- ---------- ---------- ---------M SLSFLA---- TTTP-TTTSF D--------- ------RR-S SK-----ATH
VviPPO1     ---------- ---------- ---------- ---------- ---------M ASLPWSLTTS TAIANTTNIS A--------- ------FPPS PLFQRASHVP
VviPPO3     ---------- ---------- ---------- ---------- ---------M ASLPWSLTTS TAIANTTNIS A--------- ------LPPS PLFHRVSHVP
AcoPPO5     ---------- ---------- ---------- ---------- --------MG SLSPLN---- YNTITTTSSP L--------- ------YP-F PLKKTKISAF
VviPPO2     ---------- ---------- ---------- ---------- --------MA SLSPPA---- TTTT-TNISS L--------- ------QP-S PLFHKASQVS
PtrPPO3     ---------- ---------- ---------- ---------- ---------M ASISPS---- TTTPTTISSS T--------- ---------F FPSFPKTSQL
MesPPO1     ---------- ---------- ---------- ---------- ---------M GSFFPS---- TLSTSSFFFP S--------- ------LPNT SQ-----ISI
RcoPPO1     ---------- ---------- ---------- ---------- --------MS SFTTSI---- TIST-SSFCP F--------- ------LSKT SQ-----LSI
PtrPPO11    ---------- ---------- ---------- ---------- --------MA YNLSPL---- TTSPSAISTS S--------- ---------L CPSILKKHAP
PtrPPO1     ---------- ---------- ---------- ---------- ---------- ---------- MSTLSFSPFF P--------- ---------- -----KPQHV
PtrPPO12    ---------- ---------- ---------- ---------- ---------- ---MAS---- CISLSSSIPL A--------- ------ASSF LPSFPKTHRV
PtrPPO5     ---------- ---------- ---------- ---------- ---------- ---MAS---- CISLSSSIPL A--------- ------ASSF LPSFPKTHQV
PtrPPO14    ---------- ---------- ---------- ---------- ---------- -MASCI---- CLSS-SIPLA A--------- ------SS-F LPSFPKTHRV
PtrPPO2     ---------- ---------- ---------- ---------- ---------- -MASFI---- SLSS-SIPLA A--------- ------SS-F LPSFPKTHRV
PtrPPO9     ---------- ---------- ---------- ---------- ---------- ---------- ---------- ---------- ---------- -------MRV
PtrPPO15    ---------- ---------- ---------- ---------- ---------- -MASCI---- CLSS-SIPLA A--------- ------SS-F LPSFPKTHRV
GmaPPO2     ---------- ---------- ---------- ---------- ---------M ASSTSHPSFW SLLNLSASIP I--------- ---------S SSVCMFPPSK
GmaPPO4     ---------- ---------- ---------- ---------- ---------M ASSTSHPSFW SLLNLSASIP I--------- ---------S SSVCMFPPSK
GmaPPO3     ---------- ---------- ---------- ---------- ---------M ASISSHPSFS SLLSFSASFS I--------- ---------P SMFPTSQKPC
GmaPPO5     ---------- ---------- ---------- -------MSQ KKIDHGFYIL SILFLPLQFL CTSSHFHLFF I--------- --------FH LPNFPNTMQP
GmaPPO6     ---------- ---------- ---------- ---------- --------MA YISSLSSFSL SNFSAPLPIS I--------- ----CSSSSA FLTSQIPCKP
GmaPPO8     ---------- ---------- ---------- ---------- --------MA YISSLS---- SFSL-SNFSA PLPISICSSS SAFL--TS-Q IP-----CKP
GmaPPO7     MSMSEFSCYP TTLFTTSVPY KLKGNICRSI INYKPQEDWI ISRIISWLLS SLFLVCPSLI SLHPFPISIS S--------- ------SSST FPLSQTPCKP
GmaPPO1     ---------- ---------- ---------- ---------- ---------M ACIPPQSFVS AAYNVPSKST T--------- ------LP-S SLHPFSQSQS
GmaPPO9     ---------- ---------- ---------- ---------- ---------M ASKCPFSFVS VVNNASSNSS T--------- ------TPPS SLHPISQFQS
GmaPPO10    ---------- ---------- ---------- ---------- --------MA SISPLSFVPT VNNV-SSNSI A--------- ------PP-S PLHPFSRFQS
PpaPPO4     ---------- ---------- ---------- ---------- ---------- ---------- ---------- ---------- ---------- ----------
SmoPPO6     ---------- ---------- ---------- ---------- ---------- --MFPA---- ALSLDRIKLQ H--------- ---------- -------RSQ
SmoPPO5     ---------- ---------- ---------- ---------- ---------- --MSSA---- ALSLDRVKLL H--------- ---------- -------RSP
SmoPPO7     ---------- ---------- ---------- ---------- ---------- --MSSA---- ALSLDRVKLK H--------- ---------- -------RSR
SmoPPO8     ---------- ---------- ---------- ---------- ---------- ---------- MSPAALSLDR I--------- ---------- -------KLQ
SmoPPO9     ---------- ---------- ---------- ---------- ---------- ---------- -----MVALS I--------- ---------- --------DR
SmoPPO4     ---------- ---------- --------MV ALSIDRLELQ RPSRTRLLKI AANARKEANA CNSNSSPVTP S--------- ---------- -------IDR
SmoPPO11    ---------- ---------- ---------- ---------- ---------- --MCCI---- AAFFDNVFAT E--------- ---------H VWDGGTVIDR
SmoPPO10    ---------- ---------- ---------- ---------- --------MY SLPSMS---- -----GMVAL S--------- ---------- -------IDR
MguPPO2     ---------- ---------- ---------- ---------- --------MA SLALSW---- SSAA-AAAAA A--------- ------SS-Q LP-----PPS
MguPPO5     ---------- ---------- ---------- ---------- ---------M ASLPLS---- CYTT-TQFSS S--------- ------IS-T QP-----PPR
MguPPO6     ---------- ---------- ---------- ---------- ---------- --MASSVQSS CTFLLATNHR H--------- ---------S RPPPSSPHSL
MguPPO3     ---------- ---------- ---------- ---------- --------MA SLPLSCATTT TTSATPLRHS S--------- ------PPPL FTKPSLFITR
MguPPO4     ---------- ---------- ---------- ---------- ---------M ASLHIP---- CTTTATTPSS A--------- -------TRP SSRHHFAKPS
MguPPO7     ---------- ---------- ---------- ---------- ---------M TSLYLS---- CATT-TTASR H--------- ------PF-L KP-----QHV
MguPPO8     ---------- ---------- ---------- ---------- ---------M TSLYLS---- CATTNTAISA T--------- ----PSSHPR PLLPKPSHSH
MguPPO9     ---------- ---------- ---------- ---------- ------MASL HQVLPAAAPS SSSLRSSSST A--------- ---------T RPVVFAKPSH
GmaPPO11    ---------- ---------- ---------- ---------- ------MGNP SKLFPFFFAF IVFLMPLVSL S--------- ------HNDF STFAIKTVSY
SmoPPO2     ---------- ---------- ---------- ---------- ---------M GRTPSSARIL NNVLPRMVGE S--------- ---------- --------LL
SmoPPO3     ---------- ---------- ---------- ---------- ---------M GRTPSSARIL NNVLPRMVGD S--------- ---------- --------LL
SmoPPO1     ---------- ---------- ---------- ---------- -------MAA ATNSSS---- NSLL-SALDR D--------- ------PR-T DLRLGRARIQ
PtrPPO13    ---------- ---------- ---------- ---------- ---------- ---------- MEAKKLGLVL G--------- ---------I IT-----IVA
VviPPO4     ---------- ---------- ---------- ---------- ---------- ---------- ---------- ---------- ---------- -------MIL
AmePPO1     ---------- ---------- ---------- ---------- --------ME TKRWVS---- LIFL-AFILL V--------- ---------- -------LSS
AcoPPO4     ---------- ---------- ---------- ---------- --------MK GNRLLHAVLF TFIIVGLFGS L--------- ---------Q VIKDSNNEEH
AcoPPO2     ---------- ---------- ---------- ---------- --------ME AKRWLHAVLF TFVMVGLFAS L--------- ---------- --VINVSNEE
AcoPPO3     ---------- ---------- ---------- ---------- ---------- ---------- MILVSLNFFL F--------- ---------- -------RKQ
PpaPPO9     ---------- ---------- ---------- ---------- ---------- ---------- ---------- ---------- ---------- ----------
PpaPPO12    ---------- ---------- ---------- ---------- ---------- ---------- ---------- ---------- ---------- ---------M
PpaPPO2     ---------- ---------- ---------- ---------- ---------- ---------- ---------- ---------- ---------- -----MKLIC
PpaPPO10    ---------- ---------- ---------- ---------- ---------- ---------- ---------- ---------- ---------- ---------M
PpaPPO1     ---------- ---------- ---------- ---------- ---------- ---MVH---- CVVT-DNFVV I--------- ------FR-L NI-----DPE
PpaPPO7     ---------- ---------- ---------- ---------- ---------- ---------- MTSL-PSVVI A--------- ---------- ----------
PpaPPO11    ---------- ---------- ---------- ---------- ---------- ---------- ---------- ---------- ---------- ----------
PpaPPO6     ---------- ---------- ---------- ---------- ---------- ---------- ---------- ---------- ---------- ----------
PpaPPO8     ---------- ---------- ---------- ---------- ---------- ---------- ---------- ---------- ---------- ----------
PpaPPO3     ---------- ---------- ---------- ---------- ---------- ---------- ---------- ---------- ---------- -------MRE
PpaPPO5     ---------- ---------- ---------- ---------- ---------- ---------- ----MGNFLE D--------- ---------- -------GSR
PpaPPO13    ---------- ---------- ---------- ---------- ---------- ---------- ---------- ---------- ---------- ----------
BdaPPO5     ---------- ---------- ---------- ---------- ---------- MSSSVVGTMT MVPFTSSSAS A--------- ---------- -------CLL
BdaPPO4     ---------- ---------- ---------- ---------- ---------- --MASP---- CFLLLQPLAA S--------- ---------- ---------A
SitPPO3     ---------- ---------- ---------- ---------- ---------- -MATPSAKTN GLPLVASPRP P--------- ---------- -------AYP
SbiPPO6     ---------- ---------- ---------- ---------- ---------M ATPSAAASSS FLVPATTAVA S--------- ---------T PSACPSTLPK
ZmaPPO4     ---------- ---------- ---------- ---------- ---------M ATASAA---- SSFLVPATAI A--------- ---------P TPSACPSTVP
SbiPPO5     ---------- ---------- ---------- ---------- --------MN GSMASA---- CATSRPLASA P--------- ------SA-- -------CPS
ZmaPPO5     ---------- ---------- ---------- ---------- ---------- --MASA---- CATSIPLVSA P--------- ---------- ------SACP
SbiPPO7     ---------- ---------- ---------- ---------- --------MN GSMASA---- CATS-SPLVS A--------- ------PS-- -------ACP
A. marina   ---------- ---------- ---------- ---------- ---------- ---------- ---------- ---------- ---------- ----------
```

```
                   110        120        130        140        150        160        170        180        190        200
            ....|....| ....|....| ....|....| ....|....| ....|....| ....|....| ....|....| ....|....| ....|....| ....|....|
BdaPPO1     RALLAISGLD PYITSCADHD DASAARLSD- ---------- ---------- ---------- -GGGSDNI-- -I-------- -----GGP-- -IVT------
BdaPPO3     RALLAVSGLD PYIVSCAADE DAFTAPLSN- ---------- ---------- ---------- -GGNDDKN-- -I-------- -----GGP-- -IVT------
SbiPPO1     RSILAASGLD AYLLPCTTDA SKAPSSHGKD ANRTSG---- ---------- ---------- -GGNTGA--- ---------- -----RRP-- -IIT------
OsaPPO1     RALLAVTGLD PYVVSCAADD GVSTPLLSD- ---------- ---------- ---------- -GGHDKINAG RV-------- -----GGP-- -IVT------
ZmaPPO1     RTILAASGLD PRLISCADDG SAKAQLSDGD ---------- ---------- ---------- -GGHNKAGSG ---------- -----GRP-- -IVT------
SitPPO1     KTILAATGLD PQLISCAGDP ATKAPLSGY- ---------- ---------- ---------- -GGDAGNKAG SG-------- -----GRP-- -IVT------
SbiPPO8     RTILAATALD PHLISCADDG SKSNAELSEG D--------- ---------- ---------- -GGNKPAGSG ---------- -----GRP-- -IVT------
ZmaPPO2     TTKNATTRPR SLRLSCKAGD DDDG------ ---------- ------VDRR DVLLGLMAGA GAAAAGIASN NTHGSSSAL- -----AAP-- -VQA------
OsaPPO2     LLHRKTKGWK PRNVSCR--- ---------- ---------- ------VDRR DVLL------ --GISGAAAM VATQGGGGAL -----AAP-- -IQA------
BdaPPO6     GSMRSSAPRL QHPLRCISRK AAASDGDNRT NV-------- ------VDRR DVLLGL---- -GAGAAAAIG APRRAL---- -----AAP-- -IKA------
SbiPPO3     APRAPCRSLH TLRCRCKATG GA-------- ---------- -------DRR DVLLGL---- -GGAAAAGLL SSSLSNRGGA L----AAP-- -IPA------
ZmaPPO6     AFAAPCRSLQ TTTVRCKATG AA-------- ---------- -------DRR DVLLGL---- -GGAAASGLL RSSSSSSSG- -----GAP-- -IQA------
SbiPPO4     ALRAPPCCSL QTTLRCKATG GA-------- ---------- -------DRR DVLLGL---- -GGAAAAGLL TSSSSRGGAI -----AAP-- -IQA------
MguPPO1     HIPTIRKPSH RHIISCKSTN NHDQEPAGK- ---------- ------FDRR DVLIGL---- -GGLYGATTL SASPFAF--- -----AAP-- -ISA------
BdaPPO2     NSSSLKPRRA AGRVRCTLST DATGGRAEHD GPR------- ------LDRR DVLLRL---- -GTLGASATA GLLSSPRLAG -----AAP-- -VAT------
SitPPO2     RSGLRPRRAT VHRVPCASSR GEERSEPDAP K--------- ------HDRR DVLLGL---- -GALGASATL MSARRAG--- -----ADP-- -VAT------
SbiPPO2     SSGFRPRRVT VQRVSCASPR GERSEHDAPK ---------- ------HDRR DVLLGL---- -GALSASATA TLVTARRAG- -----ADP-- -VAT------
ZmaPPO3     SSGFRPRRVT VQRVSCASPR GERYELDARK H--------- -------DRR DVLLGL---- -GALGASATA TLASARRAG- -----ADP-- -VAT------
AcoPPO6     NRSTVRVGKK VAYTSCEQKQ SNEG----DS HT-------- ------IDRR NVLLGL---- -GGLYGASAT IGSQGKIAM- -----GAP-- -VAP------
AcoPPO7     NRSTVRVGKK VAYTSCEQKQ SNEGDSHT-- ---------- ------IDRR NVLLGL---- -GGLYGASAT IGSQGKIAM- -----GAP-- -VAP------
AcoPPO1     SVARTLIRKR FTPTTCEQKH EGNGVGVNAE RSG------- ------IDRR DVLVGL---- -GGLCGATAT FGSNMAV--- -----GAP-- -VQP------
VviPPO1     VARNRSCRFA PSKVSCNSAN GDPNSDSTSD VRETSSGK-- ------LDRR NVLLGI---- -GGLYGAAGG LGATKPLAF- -----GAP-- -IQA------
VviPPO3     IARNRSRRFA PSKVSCNAAN GDPNSDSTSD IRETSPGK-- ------LDRR NVLLGI---- -GGLYGAAAG LGATKPLAF- -----GAP-- -IQA------
AcoPPO5     GKPSINRVTR RVLITCNARN EESMGK---- ---------- ------LDRR NVLLGL---- -GGLCGLSGL SATDNRLAM- -----AAP-- -IMP------
VviPPO2     LVGKRNPRFV RRVVSCNATN GDERSTSSSK SGESP----- ---LGKLDRR NVLLGL---- -GGLYGVSGL GAADPFAF-- -----AAP-- -ISP------
PtrPPO3     SLIKKRNHRY TPRFSCRATN DDSQNPTT-- ---------- --------RR DLLIGL---- -GGLYGATSL SDPFAF---- -----AAP-- -VSA------
MesPPO1     NKMRNHSYVG -RVSSCKTTN DNDHQNPTT- ---------- --------RR DVLIGL---- -GGFCGATTL ADHPFAF--- -----AKP-- -ISA------
RcoPPO1     PKNRNCNYVN PHLISCKATA NDHDQNPTT- ---------- --------RR DILIVL---- -GGLYVAITL GDPLAF---- -----AKP-- -VSA------
PtrPPO11    FVARNQSHPN FHTVSCKARN DDHQNPST-- ---------- --------RR DVLIGL---- -GGLYGATNL SDPFAY---- -----AKP-- -IQP------
PtrPPO1     TKTKRLNHPY VPRVSCKATD DTQNPPT--- ---------- --------RR DVLIGL---- -GGLYSATNL ADRTAF---- -----AKP-- -ITP------
PtrPPO12    SRFKKPNRHN IPIVSCKSGK NDHEQNPAT- ---------- --------RR DLLIGL---- -GGLYGATSL SDPFAY---- -----ANP-- -IAP------
PtrPPO5     SRVKKPNRPN IPIVSCKSGK NDHEQNPAT- ---------- --------RR DLLIGL---- -GGLYGATSL SDPFAY---- -----ANP-- -IAP------
PtrPPO14    SRIKKPNRPN IPIVSCKSGK NDHEQNPAT- ---------- --------RR DLLIGL---- -GGLYGATSL SDPFAY---- -----ANP-- -IAP------
PtrPPO2     SRIKKPNRHN IPIVSCKSGK NDHEQNPAT- ---------- --------RR DVLIGL---- -GGLYGATSL SDPFAY---- -----ANP-- -IAP------
PtrPPO9     SRIKKPNRHN IPIVSCKSGK NDHEQNPAT- ---------- --------RR DLLIGL---- -GGLYGATSL ---------- ---------- ----------
PtrPPO15    SRIKKPNRPN IPIVSCKSGK NDHEQNPAT- ---------- --------RR DLLIGL---- -GGLYGATSL SDPFAY---- -----ANP-- -IAP------
GmaPPO2     KPSKATKRRH AWEVACNGNP RN-------- ---------- --------RR DILIGL---- -GGLYGATTS LTSNNTGSAF -----GAS-- -LSP------
GmaPPO4     KPSKATKRRH AWEVACNGNP RN-------- ---------- --------RR DILIGL---- -GGLYGATTS LTSNN----- ---------- ----------
GmaPPO3     QPSKPKRGRH ASKVACNGNP NP-------- ---------- ------RNRR DILIGL---- -GGLYGATTL SGNSTGSAF- -----GAP-- -VSP------
GmaPPO5     TKRSKPKRHH VSKVTCNSNQ NTPTPNPEEE RPSYN----- ---ILGKYRR DVLLGI---- -GGLYGASAL SNTNPLAMA- -----AAP-- -ILQ------
GmaPPO6     SKRSKPKGHH VSKVSCNSNQ NTPTPNPEEE KPSSY----- --NILGKHRR DILLGI---- -GGLYGASAL SNTNPLAMA- -----AAP-- -ILE------
GmaPPO8     TKRSKPKGHH VSKVSCNSNQ NTPTPNPEEE KPSSY----- --NILGKHRR DVLLGI---- -GGLYGASAL SNTNPLAMA- -----AAP-- -ILE------
GmaPPO7     TKHSKPKRHH VSKVSCHSNQ NNSAPNPEEG KPSHN----- --IVAGKNRR DVLIGF---- -GGLYGASTL TNNNNNNPLA I----AAP-- -ILP------
GmaPPO1     TKYRKSKHHH TPRVTCNSGN QNKGEKPDIH I--------- ------EQRR NILLGL---- -GGLCGAATL NNNPFAF--- -----AAP-- -ISP------
GmaPPO9     TKNRKPKRHH IPRTTCSENQ NNPTPNPSEG ELSHI----- -----VGHRR NVLLGL---- -GGLCGAVTL NNNPFAF--- -----AAP-- -ISP------
GmaPPO10    IKNRKPKPHH IPRITCSGNQ NNPTPNPNSQ GEPPH----- ----IVGHRR NVLLGL---- -GGLCGAVTL NNNNPFAF-- -----AAP-- -ISP------
PpaPPO4     ---------- --MYTCK--- ---------- ---------- ---------- ---------- ---------- ---------- -----KHA-- -VVP------
SmoPPO6     CPLLPNRSRG ALKITASARK EANAAAGESG NPSFPSKKGS STPASIIDRR ELLA------ -GTIAGGASL AASPTGPVL- -----AEP-- -VGS------
SmoPPO5     CPLLPNRSRG ALKITANARK EANAATGESG NPSFPSKKGS STPASIIDRR ELLA------ -GTIAGGASL AASPTGPAL- -----AEP-- -VGS------
SmoPPO7     CPLLPNRSRG TLKITANARK EANAATGESG NPSFPSKKGS STPASIIDRR ELLA------ -GTIAGGASL AASPTGPAL- -----AEP-- -VGS------
SmoPPO8     HRSRCPLLPN RFKITANARK EANAAKSSN- -PSFPSKKGS STPASIIDRR ELLA------ -GTIAGGASL AASPSGPAL- -----AEP-- -VGS------
SmoPPO9     LELQRPSRTR PLKIAANARK EANACNSNSS PVTPS----- ------IDRR ELLA------ -GAIAGGASL ASSVDSHAL- -----ADP-- -IGL------
SmoPPO4     LELQRPSRTR PLKIACNSNS SPVTPS---- ---------- ------IDRR ELLA------ -GAIAGGASL ASSVDSPAL- -----ADP-- -IGP------
SmoPPO11    LELQRPSRTR PLKIAANARK EANACNSNSS PVTPS----- ------IDRR ELLA------ -GAIAGGASL APSVDSPAL- -----ADP-- -LGL------
SmoPPO10    LELQRPSRTR LLKIAANARK EANACNSNSS PVTPS----- ------IDRR ELLA------ -GAIAGGASL ASSVDSHAL- -----ADP-- -IGL------
MguPPO2     NSSVKTQTRR CNNITCKATN NDKNTEPTQK ---------- ------LDRR NLLLGI---- -GGVYGGAAH LLSADNPPAA F----AAP-- -ITS------
MguPPO5     QRFSAQTPPP TRRFQVRSTG GPNNEKGENS PPNGG----- -----KFDRR NLLMGM---- -GGLYSAANL VPTDSPAS-- -----ATP-- -ISS------
MguPPO6     TPAGRHRRSH LFQVSCKQQN PPPPSSEGK- ---------- ------VDRR NMLLGL---- -GGLYGAANL IPTNPEAAT- -----ASP-- -IEP------
MguPPO3     HANGSHHHRG LHVISAQKQN DNPESTHPQG K--------- ------LDRR NVLLGM---- -GGLYGAAAS LVSTPPEAS- -----ANP-- -VQA------
MguPPO4     HLITHAKRNN RLQISCSDSQ KQKDSSQNAD TPQGN----- ------VDRR NVLLGF---- -GGLCGAANL ISTHTAS--- -----ANP-- -VQA------
MguPPO7     ITTHAKPSHP LHQISCSSTT SQNQNDDTKS QSGKV----- ------VDRR NVLLGM---- -GGLCGAANL VSNTPAAS-- -----ANP-- -ILP------
MguPPO8     FITHAKLTNR LHHVSCSSTP QNQNDETKKP TAKV------ ------VDRR NMLVGM---- -GGLYGATTL ASTPSAA--- -----ANP-- -IQA------
MguPPO9     FLTHAKRNHH RRLISCSSSS SNEGSK---- ---------- ------VDRR NMLLGL---- -GGMYGAANL LSTPDSAS-- -----ANP-- -IQA------
GmaPPO11    LVSFSENPNH NGHITTSSNE RDKSRLWR-- ---------- ---------- ---------- -KAFIGLKNT HEPSSNI--- -----SRA-- -ISL------
SmoPPO2     SRLLVTKFYF PVQILCFLLL STATGRR--- ---------- ---------- ---------- -EASLGLATG SLQQEQQAW- -----ARP-- -IDN------
SmoPPO3     SRLLVTKFYF PVQILCFLLL STATGRR--- ---------- ---------- ---------- -EASLGLATG SLQQEQQGW- -----ARP-- -IDN------
SmoPPO1     FGGRSGKRLC TTTIKCVHEG EEHARRSGRT SD-------- ------VNRR EFVL------ -GSIGGGAAA TLPAAGAL-- -----ANP-- -VEL------
PtrPPO13    AFTRILHILE APEVQHALLG EFN------- ---------- ---------- ---------- -FIIPGMLKL ASSPGKDQQ- -----EKP-- LVIL------
VviPPO4     GISVGEISPQ HHDVDAKKH- ---------- ---------- ---------- ---------- ---------- ---------- -----AKN-- -VVS------
AmePPO1     DLFLTDSQQQ MKPISF---- ---------- ---------- -----IRDLK KTILGIFD-- -GTWVSMETT SSSSTSEK-- -----KNPKF VVLS------
AcoPPO4     THMREQKNQQ VSIISTSFLW FKNIASSLAF ---------- ---------- ---------- -ADWQFNKVP VSKNP----- -----EKPQK FGIS------
AcoPPO2     HTHMREEKNK LVSISTTFLW LKKIVSSLAF ---------- ---------- ---------- -GDWQFNKVP VSEHP----- -----EKPHR VGLS------
AcoPPO3     NDMNIVQNNQ QVSISTPFLW LKNIVSSFAS ---------- ---------- ---------- -GDWQWDKVP VSKHP----- -----DKPHR VVFS------
PpaPPO9     ---------- -MKFTCVLL- ---------- ---------- ---------- ---------- -VVLAVLLAL NLRVD----- -----GAP-- -FPP------
PpaPPO12    NSVYVALAVV ---------- ---------- ---------- ---------- ---------- -AALFALCPQ VE-------- -----GAP-- -FPP------
PpaPPO2     VLLELLVVVV --LLMC---- ---------- ---------- ---------- ---------- -AQVY----- ---------- -----GAP-- -FPA------
PpaPPO10    KLNYVSGVAV AVLLACVLVE ---------- ---------- ---------- ---------- ---------- ---------- -----GAP-- -FPA------
PpaPPO1     DVSSLTRRMS TSQIMTTFTG AMMALTF--- ---------- ---------- ---------- -LAIIGIITL VQTVE----- -----GKP-- -FPA------
PpaPPO7     ---------- -LAVLCAV-- ---------- ---------- ---------- ---------- -GSIALVKIV D--------- -----GKP-- -FPA------
PpaPPO11    -------MAS RIASLC---- ---------- ---------- ---------- ---------- -GVLATVFVI YLIAPTE--- -----GKP-- -FPA------
PpaPPO6     --MKVMDQLR RVPISAMK-- ---------- ---------- ---------- ---------- -GVIYAAVLS MVFAWTEATT RPRGPGGP-- -MPA------
PpaPPO8     ---------- ---MKCAALV ---------- ---------- ---------- ---------- -LVLSVSFTL VEATT--GK- -----SRA-- -LPA------
PpaPPO3     VYAERGFEMG GLVLARNF-- ---------- ---------- ---------- ---------- -GVVTWGSIL AMLIVNVVT- -----AVP-- -LPP------
PpaPPO5     KDLTGFKMRA AVLILSQIL- ---------- ---------- ---------- ---------- -GDFTS---- ---------- -----GVP-- -IPA------
PpaPPO13    ---------M LMRITS---- ---------- ---------- ---------- ---------- ---------- ---------- -----GTP-- -VPA------
BdaPPO5     PLTSKPSKHG RRRLSCKAMA SSGDDDDSYR ---------- ------IDRR DVLLGLTAAT TGSTLGRARG LLAAEGV--- -----KQP-- -MPI------
BdaPPO4     SATSCTNKRR PRRVSCNATP SSRSSTPTGD GEEDM----- --QPRRLDRR EVVLV----- -GGLTGAAAA NLGLMPALAG DD---TEP-- -AAT-CITAP
SitPPO3     SMLPKKPTQR RRSLSCRAAA PR-------- ---------- ------FDRR DVLAGLTGVA AGGLATRPGL AATEDASD-- -----VCPRG EKVT------
SbiPPO6     KNAAGAGRRR HRTMQCRASS GGGRRGDGDD VDSRL----- ----LWLPRR QVLTGL---- -SGVAAGFVG YPDDLASIAL ALE--ANP-- -VESCRRGEK
ZmaPPO4     KNKKNAAGRR RRTLQCRASG RRGDDEDSR- ---------- ---LLWLPRR EVLTGL---- -GGVAASFVG YPDLASIALE -----ANP-- -VESCRRGEK
SbiPPO5     KKTTAARFRR RRTVSCRATA GGDNDGLLWP ---------- ------LPRR DVML------ -NGLTGVAAG LAWYPGVAS- -----GADSS TTSPCTAADK
ZmaPPO5     SKKTTVARFR RRTATCRASS GGGGGRGGEN D--------- --GLLWLPRR DVMLNGLSSV AAGLAWYPGV ASGADAVCTR -----ADK-- -VNE------
SbiPPO7     SKKTAARFRR RRTVSCRATG GDND------ ---------- --GLLWLPRR DVML------ -SGLSGVAAG LACYPGLADA AECTRSDK-- -VNE------
A. marina   --MFTSKTRR EFLISA---- ---------- ---------- ---------- ---------- -GAATALF-- ---------- ---------- -FLP------
```

```
             210        220        230        240        250        260        270        280        290        300
      ....|....| ....|....| ....|....| ....|....| ....|....| ....|....| ....|....| ....|....| ....|....| ....|....|
BdaPPO1    --NLLT--CG NA-------T LPPHAL---P PFYCC----- ----PPMTT- ---AEPINF- TFP----DPS EPLRVRRPAH --A------V GAEY-MAKYE
BdaPPO3    --NLLT--CG KP-------K LPPHAL---P PFYCC----- ----PPMSA- ---SEPIDF- TFP----DPS EPLRVRRPAH --A------V GAEY-MAKYE
SbiPPO1    --DQL---CV KG-------V LPPHAL---P PFQCC----- ----PPPPST SSASGPINF- TFP----DPA EPLRTRRPVH --V------V GAEY-MAKYA
OsaPPO1    ---DLLQ-CR KP-------E GPDFP----E DLQCC----- ----PPMPT- ---SEPIDF- TLP----DPS EPLRTRRPAH --V------A GAEY-MAKYE
ZmaPPO1    ---DLLS-CG EP-------R LPSHAL---P PFRCC----- ----PPTPAS D--AAVANF- TFP----DPG EPLRTRQPAH -GA------A GADS-VARYA
SitPPO1    ---DLRW-CG EP-------S LPPHAL---S PFHCC----- ----PPAPVS E--PAVINF- TFP----DPA APLRTRRPAH --D------A GAAGDMAKLA
SbiPPO8    ---DLLS-CG EP-------D LPSHAL---P PFRCC----- ----PPTPPS G--TPIANF- TFP----DPG EPLRTRRPAH --ESGSGSGT DNSV-VARYA
ZmaPPO2    --PDLQA-CR TP-------D VPATA----A DPSCC----- ----MTYRAG LAPPAIIDF- QPPRASSSSS SPLRVRPAAH --L------V DSAY-VAKYE
OsaPPO2    --PDLGD-CH QP-------VD VPATA----P AINCC----- ----PTYSAG ---TVAVDF- APP----PAS SPLRVRPAAH --L------A DRAY-LAKYE
BdaPPO6    --PDLRD-CH AP------ED LPEAIT---G ATNCC----- ----PTYDG- ---TGIVDF- ELP-VSAGSS PVTRVRPAAQ --L------V DAEY-VAKYE
SbiPPO3    --PNLSN-CH PP-------D LPDTV----S DINCC----- ----PPGAG- ---TAIVDF- TPP-----PT VGRRVRPAAH --L------V DEEY-VAKYE
ZmaPPO6    --PDLRN-CH PP-------D LPDTA----E DVNCC----- ----PS---- ---PGIVDF- TPS------- PRLRVRPAAH --L------V DAEY-VAKYE
SbiPPO4    --PDLRD-CH PP-------D LPDTV----G DVNCC----- ----PPGAG- ---TAIVDF- TLP-----PA VGLRVRPAAH --L------V DEEY-LAKYE
MguPPO1    --PDVTK-CG PA-------D LPQGA----A PTNCC----- ----PPPT-- ---ATIVDY- KFP----PPS TTMRVRPAAH --L------A DKAY-VAKFN
BdaPPO2    --PDISS-CG KP-----DLG LPPNA----N LLTCC----- ----PPPSN- ---ALPVDF- SPP----DAS TPLRTRPAAH --S------V GADY-VAKLN
SitPPO2    --PDIST-CG PA-------D LPPGA----N VVTCC----- ----PPPSA- ---ALPVDF- TPP---DAAS SPLRTRQPAH --S------V TADY-VARLN
SbiPPO2    --PDISS-CG PA-------D LPPSA----N VLTCC----- ----PPPSN- ---ALPVDF- TLP---DATS LPLRTRPAAH --S------V TADY-VAKFN
ZmaPPO3    --PDISS-CG QA-------N LPVSA----N VLTCC----- ----PPPSS- ---ALPVDF- ILP---DATS LPLRTRPAAH --S------V TTDY-VAKFN
AcoPPO6    --PDLSK-CQ LA-------- ---TDAANGE QVYCC----- ----PPYSS- ---ADIKPF- VPP-----ND GILRKRKPAH --K------L NRKE-IEDFK
AcoPPO7    --PDLSK-CQ LA-------- ---TDEANGE QVYCC----- ----PPYSS- ---ADIKPF- VPP-----ND SILRKRKPAH --K------L NRKE-IEDFK
AcoPPO1    --PDLSA-CR LA-------- ---NDLQLVN RVDCC----- ----PPYGT- ---TTITDF- VLP----DQS EPMRVRRSAE ALA------S DPVY-LEKFR
VviPPO1    --PDISK-CG TA-------T VPDGV----T ATNCC----- ----PPVT-- ---TKIIDF- QLP----SSD SPMRTRPAAH --L------V SKEY-LAKYK
VviPPO3    --PDIRK-CG TA-------T VPNGV----I PTNCC----- ----PPVT-- ---TKPIPF- QLP----SSD SPMRTRPAAH --L------V SKEY-LAKYK
AcoPPO5    --PDLSK-CG PA-------D FPPGS----Q PTNCC----- ----PPTD-- ---LKIVDF- KLP----SRS TPMRVRPAAH --L------V DDEY-IAKYS
VviPPO2    --PDLSE-CG AA-------D LPAGA----Q PTNCC----- ----PPVS-- ---SKIIDF- KLP----PKN SPLRIRPAAH --L------A SKEY-IAKYK
PtrPPO3    --PDISK-CG RA-------D LPTGA----N ETNCC----- ----PPVPS- ---TKILDF- KRP----PSN APLRVRPAAH --L------A DKDY-IAKYK
MesPPO1    --PDITK-CG EA-------D FPAGA----K PTNCC----- ----PPQS-- ---TKILDF- KLP----SSN SPLRVRPAAH --L------V DESY-VAKYS
RcoPPO1    --PDLTK-CG KA-------D LPSSA----K PTNCC----- ----PPPS-- ---TKILDF- KLP----SSN SPLRIRPAAH --V------V DDAY-LAKYS
PtrPPO11   --PDIAD-CS VI-------N EPDPE----N PTNCC----- ----PPLT-- ---RKIIDF- KLP----SQN EPLRIRRPAH --L------V DDNY-IAKYN
PtrPPO1    --PDLTK-CE LV-------D LPNPE----N PSNCC----- ----TPLP-- ---KKIIDF- RPP----SPF SPLRTRRAAH --L------V DEDY-VAKYA
PtrPPO12   --PDITQ-CE LV-------T LPTES----D PSNCC----- ----PPTS-- ---TKIKNF- EFP----SAS SPMRIRPAAH --L------V DKAY-VAKYA
PtrPPO5    --PDITQ-CE LV-------T LPSES----N PTNCC----- ----PQTS-- ---TKIKNF- EFP----SAS SPMRIRPAAH --L------V DKAS-LAKYA
PtrPPO14   --PDITQ-CE LV-------P LPSES----D PSNCC----- ----PPTS-- ---TEIKNF- EFP----SAS SPMRIRPAAH --L------V DQAY-VAKYA
PtrPPO2    --PDITQ-CE LV-------T LPTES----D PSNCC----- ----PPTS-- ---TKIKNF- EFP----SAS SPMRIRPAAH --L------V DKAY-LAKYA
PtrPPO9    ---NITQ-CE LV-------P LPTES----D PSNCC----- ----PPTS-- ---TKIKNF- EFP----SAS SPMRIRPAAH --L------V DKAY-VAKYA
PtrPPO15   --PDITQ-CE LV-------T LPTES----D PSNCC----- ----PPTS-- ---TKIKNF- EFP----SAS SPMRIRPAAH --L------V DKAY-VAKYA
GmaPPO2    --PDPTN-CV QP-------- -------DPE KDPFC----- ----PP---- ---PPFKDY- ELP-PHDDKT LPLRIRPAAH --L------V TDDY-IAKYE
GmaPPO4    --------TG SA-------- ---------- ---------- ---------- ---------- ---------- ---------- ---------- -----FAKYE
GmaPPO3    --PDPTN-CV PP-------- LLPGE----N DVNCC----- ----PP---- ---SPIVDF- EFP-----SH KRLRHRRPAQ --W------V DDDY-LNKYK
GmaPPO5    --PDLEH-CC IT-------D DVPPKGVIEA QVYCC----- ----PPRSS- ---SPPIDF- KLP-----KG TPLRVRPPAQ --F------V TDEY-LEKYK
GmaPPO6    --PDLEH-CC IT------DD VPKGEIE--K QVYCC----- ----PPKSS- ---SPPIDF- KLP-----KG TPLRVRPPAQ --F------V TDEY-LEKYK
GmaPPO8    --PDLEH-CC IT------DD VPPKGVIE-A QVYCC----- ----PPKSS- ---SPPIDF- KLP-----KG TPLRVRPPAQ --F------V TDEY-LEKYK
GmaPPO7    --PDLKT-CG PP-------D LATGA----K PVNCC----- ----PPIS-- ---STIIDF- KLP----SSG APLRVRPAAH --L------V NDVY-IAKYR
GmaPPO1    --PDLTT-CG PP-------D LPEGA----E PTNCC----- ----PPFS-- ---STIIDF- KFP----PSN KPLRVRPAAH --L------V DKNY-LAKYK
GmaPPO9    --PDLNT-CG PP-------D TPAGA----N PTNCC----- ----PPS--- ---SKIIDF- KFP----PSN QPLRVRPAAH --L------V NDEY-LAKYK
GmaPPO10   --PDLNT-CG PP-------D LPAGV----K PTNCC----- ----PPS--- ---SKIIDF- KFS----PSN QPLRVRPAAH --L------V NDEY-LAKYK
PpaPPO4    --PYSTV--- ---------- ---------V S--CC----- ----PPLPT- ---RPIKDF- KFE----DQT LPMRVRRPAH --K------V DAAY-IEKYN
SmoPPO6    --PTFKR-CQ PA-------K IENTN----T FVACC----- ----PPKPK- ---RDTIDF- KPD------T GPMRTRPAAH --L------V DEDY-IQKYN
SmoPPO5    --PTFKR-CQ PA-------N IANTN----T FVACC----- ----PPKPK- ---RDTIDF- KPD------T GPMRTRPAAH --L------V DEDY-IQKYN
SmoPPO7    --PTFKR-CQ PA-------N IANTN----T FVACC----- ----PPKPK- ---RDTIDF- KPD------T GPMRIRPAAH --L------V DEDY-IQKYN
SmoPPO8    --PTFKR-CQ PA-------N IANTN----T FVMCC----- ----PPKPK- ---RDTIAF- KPD------T GPMRTRRPAH --L------V DEDY-IHKYN
SmoPPO9    --PSFKH-CH FP-------- ----SVGDDI IRVCC----- ----PPKPK- ---RATIDF- KPD------S GQMRVRPAAH --L------V DKDY-IAKYN
SmoPPO4    --PTFKR-CH QA-------- ----TIGDDI NRACC----- ----PPKPK- ---GATSYF- KPD------S RPMRVRPAAH --L------V DKDY-IAKYN
SmoPPO11   --PSFKH-CH RA-------- TISDD----I NRRCC----- ----PPQPK- ---RATIDF- KPD------S GPMRVRPAAH --L------V DKDY-IAKYN
SmoPPO10   --PSFKH-CH RA-------- TISDD----I NRRCC----- ----PPQPK- ---RATIDF- KPD------S GPMRVRPAAH --L------V DKDY-IAKYN
MguPPO2    --PDISK-CT TG-------- TNLNTQKP-L DINCC----- ----PPQS-- ---AEIIDY- QLP-----PV TQMRYRPAAH --L------A GKEY-YAKFE
MguPPO5    --PDLSK-CS RG-------- TDLSTQQP-L DVNCC----- ----PPVV-- ---DGITNY- KLP-----PV TKMRFRPAAH --L------A GKEY-IAKYE
MguPPO6    --PEFKA-CG AA------RD VQSGDL---L NINCC----- ----PPTS-- ---DKIVDF- KLP----PPP AKLRVRPPAH --R------V SKEY-LAKFE
MguPPO3    --PQLDK-CG TA-------- -TDLNTGKKL DLNCC----- ----PPVT-- ---DNIIDY- KIP-----PA LRMKLRPSAH --R------V SAQY-MFKYN
MguPPO4    --PQIDK-CG VA-------- -TNLNTGKQL DINCC----- ----PPMG-- ---KNIIDF- KPP-----PL VQMRTRPSAH --R------V SAEY-MFKYN
MguPPO7    --PELNK-CG TA-------- TNWNNGESLD GINCC----- ----PPLT-- ---TDIIDY- KLP-----PV FRMKIRPSAH --R------A SAEY-MYKYN
MguPPO8    --PELNK-CG TA-------- -TNLNNGEKL DINCC----- ----PPIS-- ---DRIIDY- KLP-----PV FQMKIRPSAH --R------V SPEY-MFKFN
MguPPO9    --PQIDK-CG NS-------- TYDTSAGVAT QINCC----- ----PPFT-- ---GTIIDY- KLP-----NF PKTKVRPSAH --R------L SPEY-LFKFN
GmaPPO11   ---NARE-CF PV-------E LPSDAI---T STRCC----- ----PPRPSP ---SNIIDFK DFA----SPN ATLRVRKPAH --M------V DEEY-IAKLE
SmoPPO2    GTIDLEW-CT QA-------E TPAGK----- -VGCC----- ----PPRST- ---TSVVDF- VHN-----TQ LPSRLRQPVH --L------L SSDY-IAKYI
SmoPPO3    GTIDLEW-CT QA-------E TPAGK----- -VGCC----- ----PPRSK- ---TPVVDF- VHN-----TQ LPSRLRQPVH --L------L SSDY-IAKYI
SmoPPO1    --GSISS-CS PA-------E LPPGA----T PVNCC----- ----PPPNS- ---SPIVDF- QYN-----TR LPQRLRRPAH --L------L RPDE-IARYN
PtrPPO13   --PNLTS-CH ES-------- MGRSD----L PVYCC----- ----PPMNQS N--VAIIDF- QFP----DPS LPLRLRRPTH --L------L DDNY-ISKYK
VviPPO4    --PNLTT-CH ES-------- VPRPG----L SVYCC----- ----PPKPES E--EPFIDF- QFP----DPS SPLRLRRAAH --L------V DDDY-IAKYR
AmePPO1    --PNLTT-CH KS-------- LSDAD----R PVYCC----- ----PPKRES L--EPIIDF- QFP----DIS SPIRVRKPAH --L------V DADY-IAKYN
AcoPPO4    --PNLSS-CH SS-------- -FANPVLVSM PVYCC----- ----PPNIES D--EQIIDF- EFP----DPS EQLRIRKRAH --M------R DSDY-IAKYA
AcoPPO2    --PNTSS-CH KS-------- FAKPG----F QVYCC----- ----PPKPES E--EPVIDF- EFP----DPS VPLRIRKRVH --L------R DSDY-IAKYT
AcoPPO3    --PNLSS-CH NSTNIWDFDP IHPVA----F PVYCC----- ----PPKSES N--EPVIDF- EFP----DLS APLRVRKRAH --L------P DRDY-IAKYA
PpaPPO9    --PQFRL-CQ SA-------- ---------- -DRCC----- ----PPVSQ- ---KPPVPF- QFQ-----TG LPWRTRIAAQ --F------A DAKY-IAKYQ
PpaPPO12   --PQFRN-CR SL-------- ---------- -DGCC----- ----PPVNR- ---KPPVPF- QLP-----TN LPWRTRRAAQ --L------V DAAY-IAKYH
PpaPPO2    --PQIRY-CS NT-------- ---------- -TGCC----- ----PPPDA- ---RVPVDF- FFQ-----PG LPMRVRSAAQ --F------V DGKY-IAKYR
PpaPPO10   --PLIKL-CK ST-------- ---------- -QGCC----- ----PLPDS- ---RTPVDF- TFR-----PN LPIRIRRAAH --L------A DDAY-IAKYR
PpaPPO1    --PDLKL-CN YT-------- -------VRS FEGCCT---- ----LPVRL- ---GSLKKF- DFQ-----PQ LPMRVRPAAH --L------I DDAY-IAKYQ
PpaPPO7    --PDFKL-CN LA-------- -------FGS TEACCT---- ----LPERL- ---NPLKEF- DFQ-----PH LPMRVRPAAH --L------V DDAY-IAKYQ
PpaPPO11   --PDLKL-CS YA-------- -------FNS TEGCCT---- ----LPAPP- ---GPVKKF- DFK-----PR LPMRVRQAAH --L------V DDAY-IAKYQ
PpaPPO6    --ANLPEDCN TL-------- ---------- -ERCC----- ----MPKPYT GT-PGIRQF- EYE-----PN LPIRVRRPAH --L------L TKKE-IARLE
PpaPPO8    --ANLPEDCN TI-------- ---------- -ERCCMLHAK AIYGMPRPYT GK-PGIRSF- EFP-----KD QPTRIRKPAQ --H------L DKAY-VKKLE
PpaPPO3    --PDVAEYCN SE-------- ---------- -ERCC----- ----MPRPYV G--QPAHQF- YPD-----PE LPIRIRRPVH --L------L TDAE-VARLE
PpaPPO5    --PEFPEKCT SE-------- ---------- -ENCC----- ----MPYPYT G--KPVRQF- TID-----PN LPLRIRRPVH --K------L NDDE-IAKLE
PpaPPO13   --PILPDNCT SE-------- ---------- -EGCC----- ----MPQPYT G--KPARDF- EGD-----LA LPIRIRRPVH --K------L NESE-IARLE
BdaPPO5    -TSEVLK-CV SA-------- ---------- GDFVC----- ----PAGSTD YDDATVVNFS DLP----APT GPPRVRRPAH --L------L SAQE-VEKLE
BdaPPO4    ITDKLIK-CV SS-------- ---------S DGFRC----- ----PVAYLP ---EDVIDL- ASLPGPPPTG GPLRVRRPAH --L------L DAAY-VKKYE
SitPPO3    --DTLLT-CQ KT-------- ---------- GQKPC----- ----PPTSP- ---VAAVDF- TPP------T GPTRLRQPAH --L------A DPET-VEKYR
SbiPPO6    VTDKIVE-CS DP-------- ---------N RGFPC----- ----PPATR- ---IPVVDF- TPE------P KVTRIRQPAH --L------L DAEY-QAKYK
ZmaPPO4    VTEKLVE-CS DP-------- ---------N RDFPC----- ----PPASR- ---VPIVDF- TPE------A RVTRVRRPAH --L------L DPEY-QEKYR
SbiPPO5    VNEKVLQ-CT DT-------- ---------A NQKPC----- ----PLVSP- ---SSPVDF- TPE------T KVKRVRQAVH --L------L SREN-QEKYK
ZmaPPO5    --KTVQ--CT DP-------- ---------A GQLPC----- ----PLVSP- ---TDPVDF- KPE------S KVTRIRQPVH --L------L SREY-QEKYK
SbiPPO7    --NTVQ--CT DT-------- ---------E GALPC----- ----PLVSTT ---APVVDF- TPE------T KVTRVRQPVH --L------L SQEY-QEKYK
A. marina  --------TK VA-------- ---------- ---------- ---------- ---------- --------QA QELRTRKNVA --S------P DAEGDLASLR
```

```
             310        320        330        340        350        360        370        380        390        400
             ....|....| ....|....| ....|....| ....|....| ....|....| ....|....| ....|....| ....|....| ....|....| ....|....|
BdaPPO1      RVIALMKALP H---SDPRSF YQVANIHCAY CTT-SYR-Q- ------ANP- -------KLG VQ-IHFSWLF FTFHRAHLYF FERIAAKLLG EPEFALPFWS
BdaPPO3      RAIALMKALP D---SDPRSF YQMANIHCAY CTG-SYR-QT ------AHR- -------ELN VQ-IHFSWFF FAFHRAYLYF FERIAAKLLG EPGFAVPFWS
SbiPPO1      RAVALMKALP Q---SDPRSF YQQANIHCAY CTG-AHR-QL ------GYP- -------ELG IQ-IHFSWLF FPFHRAYLYF FERIAAKLLG DPAFALPIWS
OsaPPO1      RAIALMKALP R---SDPRSF YQQAHIHCAY CTG-AYR-QV ------GHP- -------ELA VQ-VHFSWLF FPFHRAYLYF FERIAGKLLG DPGFAVPFWS
ZmaPPO1      RAVALMKALP E---SDPRSF YQQANIHCAY CTA-AYR-QA ------GRP- -------ELH VQ-IHFSWLF FPFHRAYLYF FERVAARLLG DPGFAVPFWS
SitPPO1      RAVALMKALP A---SDPRSF YQQANIHCAY CAG-AHR-QA ------GRP- -------ELP LQ-IHASWLF FPFHRAYLHF FERIAARLLG DPGFAVPFWS
SbiPPO8      RAVALMKALP E---SDPRSF YQQANVHCAY CAA-AYR-QA ------GRP- -------ELP LQ-IHYSWLF FPFHRAYLYF FERVAARLLG DPGFAVPFWS
ZmaPPO2      RAVALMRELP D---DDPRSF AQQAHVHCAY CNG-AYG-QA ------GFP- -------DLD LQ-IHNCWLF FPWHRLYVYF HERILGKLIG DDSFALPFWN
OsaPPO2      RAVSLMKKLP A---DDPRSF EQQWRVHCAY CDG-AYD-QV ------GFP- -------GLE IQ-IHSCWLF FPWHRMYLYF HERILGKLIG DETFALPFWN
BdaPPO6      KAIRLMKDLP A---DDPRSF EQQWRVHCAY CDG-AYD-QA ------GFP- -------DLE IQ-IHNCWLF FPWH------ ---------- --SFALPFWN
SbiPPO3      KAVALMKELP D---DDPRSF TQQWRVHCAY CDG-AFD-QV ------GFP- -------DLE IQ-IHNCWLF FPWHRLYLYF HERILGKLIG DDKFALPFWN
ZmaPPO6      KAVALMKQLP D---DDPRSF AQQWRVHCAY CDG-AFD-QV ------GFP- -------DLE IQ-IHNCWLF FQWHRFYLYF HERILGKLVG DDKFALPFWN
SbiPPO4      KAVALMKELP D---DDPRSF AQQWRVHCAN CDG-AFD-QV ------GFP- -------DLE IQ-IHNCWLF FPWHRFYLYF HERILGKLIG DDKFALPFWN
MguPPO1      RAMELMRALP D---DDPRSF KQQAAVHCAY CDG-AYD-QA ------GFP- -------NLE LQ-VHNSWLF FPFHRYYLFF FERILGKLID DPTFAMPYWN
BdaPPO2      RAMAAMKALP A---EDPRSF AAQASIHCAY CNG-SYG-VE ------GFP- -------GSD LQ-VHNSWLF LPFHRCYLYF FERILGSLIG DPSFAMPFWN
SitPPO2      AGIAAMKALP A---GDPRSF AAQASVHCAY CDG-SYS-PN ------GFP- -------RVE LQ-VHNSWLF FPFHRCYLYF FERILGSLIG DPSFAIPFWN
SbiPPO2      AGIAAMKALP A---DDPRSF AAQATVHCAY CDG-SYS-PE ------GFP- -------DVE LQ-VHNSWLF FPFHRCYLYF FERILGSLIG DPSFAIPFWN
ZmaPPO3      AGIAAMKALP A---DDPRSF AAQASVHCAY CDG-SYS-PE ------GFP- -------GVE LQ-VHNSWLF FPFHRCYLYF FERILGSLIG DPGFAVPFWN
AcoPPO6      RGIQLMKELP E---TDPWSY YQQATIHCTY CNG-AFD-QV ------GFE- -------DVL LQ-IHGSWLF TPWHRYYIYF WEKILGKLLK DPTFAIPYWS
AcoPPO7      RGIQLMKELP E---TDPWSY YQQATIHCTY CNG-AFD-QV ------GFE- -------DVL LQ-IHGSWLF TPWHRYYIYF WEKILGKLLK DPTFAIPYWS
AcoPPO1      RAVALMKALP A---DDPWNF MQQAQIHCAY CND-AYT-QV ------GFP- -------AVS LQ-LHGSWLF LPWHRYYIYF WEKILGKLID DPTFAIPYWN
VviPPO1      KAIELQKALP D---DDPRSF KQQANVHCTY CQG-AYD-QV ------GYT- -------DLE LQ-VHASWLF LPFHRYYLYF NERILAKLID DPTFALPYWA
VviPPO3      KAIELQKALP D---DDPRSF KQQANVHCAY CQG-AYD-QV ------GYT- -------DLE LQ-VHASWLF LPFHRYYLYF NERILAKLID DPTFALPYWA
AcoPPO5      RAIALMKALP D---DDPRSF KQQANIHCAY CDG-AYD-QV ------GFP- -------NLE VQ-VHNSWLF FPFHRYYIYF HEKILGSLIG DPTFALPYWN
VviPPO2      KAIELMKALP D---DDPRSF MQQADVHCAY CNG-AYH-QV ------GFP- -------DLD LQ-VHNSWLF LPYHRYYLYF YEKILGKLIN DPTFALPFWN
PtrPPO3      KAIELMKALP E---DDPRSF MQQADVHCAY CNG-AYD-QV ------GFP- -------NLE IQ-VHNSWLF FPFHRYYLYF YEKILGKLID DPTFALPFWN
MesPPO1      KAVELMKALP D---DDPRSF KQQADVHCAY CDG-AYH-QV ------GFP- -------DLE LQ-IHNSWLF FPFHRYYLYF HERILGKLID DPTFALPYWN
RcoPPO1      KAIELMKALP D---DDPRSF MQQANVHCAY CDG-AYH-QV ------GLP- -------DLD LQ-VHNSWLF FPFHRYFLYF HEKILGKLID DPTFALPFWN
PtrPPO11     RAIALMKQLP E---DDPRNF TQQANVHCAY CDG-GYH-QV ------GMP- -------DLN YQ-VHFSWLF FPWHRYYLYF YERILGKLIN DPTFALPFWN
PtrPPO1      EAISLMKSLP E---DDPRNF YQQANVHCAY CNG-AYE-QV ------GFP- -------KLE ID-VHSCWFF FPWHRYYLYF YERILGKLIN DPTFALPFWN
PtrPPO12     KAIALMKSLP D---DDPRSF KSQADVHCAY CDG-AYH-QA ------GFP- -------DLD LQ-IHFSWLF FPWHRLYLYY FERILGKLID DPTFALPFWN
PtrPPO5      KAIALMKSLP D---DDPRSF KSQANVHCAY CDG-AYH-QA ------GFP- -------DLE LQ-IHFSWLF FPWHRAYLYY FERILGKLID DPTFALPFWN
PtrPPO14     KAIALMKSLP D---DDPRSF KSQADVHCAY CDG-AYH-QA ------GFP- -------DLE LQ-IHFSWLF FPWHRLYLYY FERILGKLID DPTFALPFWN
PtrPPO2      KAIALMKSLP D---DDPRSF KSQADVHCAY CDG-AYH-QA ------GFP- -------DLD LQ-IHFSWLF FPWHRLYLYY FERILGKLID DPTFALPFWN
PtrPPO9      KAIALMKSLP D---DDPRSF KSQADVHCAY CDG-AYH-QA ------GFP- -------DLD LQ-IHFSWLF FPWHRLYLYY FERILGKLID GPTFALPFWN
PtrPPO15     KAIALMKSLP D---DDPRSF KSQADVHCAY CDG-AYH-QA ------GFP- -------DLD LQ-IHFSWLF FPWHRLYLYY FERILGKLID DPTFALPFWN
GmaPPO2      EAVRRMQDLP P---DDPRSF MQQANVHRAY CDGRGYT-QK ------GFA- -------DYK LD-VHGSWIF FPWHRWYLYF YEKILGKMIG DPTFALPFWN
GmaPPO4      EAVRRMQDLP P---DDPRSF MQQANVHRAY CDGRGYN-QK ------GFA- -------DYK LD-VHGSWIF FPWHRWYLYF YEKILGKMID DPTFALPFWN
GmaPPO3      EAVRLMRALP L---DDPRNF MQQAKIHCAY CHN-GYR-QK ------GFP- -------DHN LQ-VHGNWLF APFHRWYLYF HERILGSLIG DPTFALPFWN
GmaPPO5      LALKRMRELP S---DDPRSF KQQADIHCAY CDG-GYK-QL ------GFPT -------ELD FK-VHFSWLF FPFHRWYLYF YERILGSLID DPTFALPYWN
GmaPPO6      LALKRMRELP S---DDPRSF KQQADIHCAY CDG-GYK-QL ------GFPV -------ELD FK-VHFSWIF FPFHRWYLYF YERILGSLID DPTFALPYWN
GmaPPO8      LALKRMRELP S---DDPRSF KQQADIHCAY CDG-GYK-QL ------GFPV -------ELD FK-VHFSWIF FPFHRWYLYF YERILGSLID DPTFALPYWN
GmaPPO7      EALKRMKALS P---NDPRNF TQQANVHCAY CDG-AYH-QV ------GFP- -------NLD LQ-VHNCWLF FPYHRWYLYF YERILGNLIG DPTFALPFWN
GmaPPO1      KAIDLMKKLP A---NDPRNF MQQANVHCAY CTG-SYD-QV ------GFP- -------GLE LQ-VHSSWLF FPYHRWFLYF YERILGSLIN DPTFALPFWN
GmaPPO9      KALDLMKKLP S---DDPRNF TQQANVHCAY CDG-AYH-QV ------GFP- -------DLD LQ-IHNSWLF FPFHRWYLYF YEKILGSLIN DPTFALPFWN
GmaPPO10     KALDLMKKLP S---DDPRNF TQQANVHCAY CDG-AYH-QV ------GFP- -------DLD LQ-VHNSWLF FPFHRWYLYF YERILGSLIN DPTFALPFWN
PpaPPO4      RAYRLMRALP Q---DDPRSF HQQSNIHCAY CGF-GFK-QL ------GI-- -------NVT LE-MHGTWLT FPFHRWYIYF HERILASLLN DSSFALPFWN
SmoPPO6      EAYAKMRALP S---DDPRSF RQQANVHCVY CNF-GYR-QE ------GDP- -------KST LQ-IHFNWLF LPWHRWYLYY HEKILGSLIN DPTFALPFWN
SmoPPO5      EAYAKMRALP S---DDPRSF RQQANVHCVY CNY-GYR-QE ------GDP- -------KST LQ-IHFNWLF LPWHRWYLYY HEKILGSLIN DPTFALPFWN
SmoPPO7      EAYAKMRALP S---DDPRSF RQQANVHCVY CNF-GYR-QE ------GDP- -------KST LQ-IHFNWLF LPWHRWYLYY HEKILGSLIN DPTFALPFWN
SmoPPO8      TAYAKMRALP S---DDPRSF RQQANVHCVY CNY-GYH-QE ------GDP- -------KST LQ-IHFNWLF LPWHRWYLYY HEKILGSLIN DPTFALPFWN
SmoPPO9      EAYAKMRALA P---DDPRSL RQQANVHCAY CSY-GYR-QE ------GAP- -------KQT LQ-IHFNWLF LPWHRWYLYF HEKILGSLIG DPSFALPFWS
SmoPPO4      EAYAKMRALP P---DDPRSL RQQAKVHCAY CNY-GYR-QK ------GAP- -------KQT LQ-IHFNWLF LPWHRWYLYF HEKILGSLIG DPSFALPFWS
SmoPPO11     EAYAKMRALS R---GDPRSL RHQAKVHCAY CNY-GYR-QE ------GAP- -------KQT LQ-IHFNWLF LPWHRWYLYF HEKILGSLIG DPSFALPFWS
SmoPPO10     EAYAKMRALS R---GDPRSL RHQAKVHCAY CNY-GYR-QE ------GAP- -------KQT LQ-IHFNWLF LPWHRWYLYF ---------G DPGFALPFWS
MguPPO2      KAVALMKALP A---DDPRSF MQQANVHCAY CNL-TYP-QT ------GDP- -------KLK LQ-IHNCWHF FPWHRWYLYF YERILGNLIG DPTFGLPFWN
MguPPO5      KAVAAMKALD KTDPTDPRGY SQQANVHCAY CNL-TYE-QS ------EDS- -------DTK LQ-IHNSWHF YPWHRWYLYF YERILGSLID DPTFGLPFWN
MguPPO6      EAIRRMKALD KSDPSDPRGF MQQANIHCAY CNG-AHD-QV ------GYK- -------DLD LQ-VHNSWLF FPFHRWYLYF YERILGSLID DPTFVLPFWN
MguPPO3      LAVDRMKRLP D---DDPRSF SQQAMIHCAY CNG-AYD-QP ------GQG- -------SLD LQ-IHNSWLF FPFHRWYLYF YERILGKLIG DPTFALPFWN
MguPPO4      TAIDRMKRLP K---DDPRSF MQQANIHCAY CNG-AYD-QP ------GQG- -------TLD LQ-VHNSWLF FPFHRWYLYF YERILGKLIG DPTFALPFWN
MguPPO7      LATDRMKRLP A---DDPRNF MQQANIHCAY CNG-AYD-QP ------GQG- -------TLD IQ-IHKCWLF FPFHRWYLYF YERILGKLIG DPTFALPFWN
MguPPO8      TAIDRMKRLP K---DDPRNF MQQANIHCAY CNG-AYD-QP ------GQG- -------TLD LQ-IHNSWLF FPFHRWYLYF YERILGKLIG DPTFALPFWN
MguPPO9      TAVDRMRRLP K---DDPRSF MQQANIHCAY CNG-GYD-QP ------GQG- -------TLD LQ-VHNGWLF FPFHRWYLYF YERILGKLIG DPTFALPFWN
GmaPPO11     KGIALMKALP D---DDPRNF IQQAKVHCAY CNG-AYHLRH ------PFQ- -------DTK LN-IHRSWFF FPFHRWYLYF FERTLGKLIG DPNFALPFWN
SmoPPO2      RAYELMKALP P---DDPRNF DQQANVHCAY CDG-AFL-YA ------NS-- -------TER LH-IHRNWFF FPWHRWFLYF HERILASLIE DDTFALPFWN
SmoPPO3      RAYELMKALP P---DDPRNF DQQANIHCAY CDG-AFL-ET ------ET-- -------GWC LTGGHRNWFF FPWHRWFLYF HERILASLIE DDTFALPFWN
SmoPPO1      RAYQLMRALP A---SDPRRF SQQANVHCAY CND-SFR-QA ------NS-- -------RSE LQ-IHDSWLF LPWHRWYLYF HERILGKLLG DDTFALPFWN
PtrPPO13     KAITIMKSLP D---TDPRSY TRQANLHCLF CTG-AYN-QQ ------GS-- -------NSP LN-IHRSWLF FPWHRMLIYF HERILGSLIG DDTFALPFWP
VviPPO4      NAVSIMKSLP Y---DDPRSF LSQANLHCIY CTG-AYK-QK ------NS-- -------DIP LH-VHRSWLF FPWHRMMLYF HERILGSLIG DDSFALPFWN
AmePPO1      KAIAIMKSLP Y---DDPRNY MRQANMHCIY CTG-AFI-SE ------HT-- -------NSL LK-IHRSWMF FPWHRMMIYF HERILGSLIG DENFALPYWN
AcoPPO4      SALSIMKSLP Y---DDPRSF ARQADVHCIY CTG-SYN-KK ------HS-- -------NEF LN-VHRSWFF FPWHRMYIYF HERILASLID DDTFSLSFWN
AcoPPO2      RALKIMKSLP Y---DDPRSF GRQADMHCIY CTG-AYN-QK ------YT-- -------NDI LS-IHRSWLF FPWHRMLIYF HERILASLIE DDTFSLSFWN
AcoPPO3      RALSIMKSLP Y---DDPRSF GRQADIHCLY CTG-AYN-QK ------YT-- -------NEG LS-IHRSWFF FPWHRMMIYF HERILASLID DDTFSLSFWN
PpaPPO9      RAYQLMKALP N---SDPRSL YAQARMHCAY CGA-AFY-YP ------RS-- -------DYS LE-IHDGWFF FPWHRMFLYF HERILASLIG DPTFALPFWN
PpaPPO12     RAYGLMRALP N---SDPRSL YAQARMHCAY CGG-AFY-FP ------NS-- -------AYP LE-IHDGWFF FPWHRMFLYF HERILASLIG DPTFTLPFWN
PpaPPO2      KAYAAMRALP R---SDPRSL YAQAQLHCAY CGG-AFK-YK ------GS-- -------AWG LE-IHGGWFF FAWHRAFLYF HERILASLIR DHTFALPFWN
PpaPPO10     LAYKRMAALP N---TDPRSL YAQANLHCAY CGG-AFK-FN ------GS-- -------SWP LE-IHGGWFF FAWHRAFLYF HERILASLIG DDSFSLLFWN
PpaPPO1      RAVEIMRALP D---TDGRSY LAQYRLHCAY CNN-HLY-YE ------GR-- -------EHP LE-IHQNWLF LPWHRLYLYF HERILAKLIG DDEFAIPYWA
PpaPPO7      RAMELMRALP E---TDGRSF QAQYRLHCAY CNNHLYY--- ------ESP- -------EQP LE-IHQNWLF LPWHRLYMYF HERILAKLIG DDEFALPYWN
PpaPPO11     KAVELMRALP E---TDGRSF TAQYRLHCAY CNN-HLH-FP ------EN-- -------EYP LE-IHQTWLF LPWHRLYLYF HERILAKLIG DDTFALPYWN
PpaPPO6      KGYKLMRALP D---TDPRSL LNQMNLHCLY CDN-GLY-FK ------GA-- -------DYP LE-VHNGWFF LPWHRMFLYF HERILGELLG DDTFALPYWN
PpaPPO8      KAYKILRELP D---SDPRSL KNQMNLHCLY CDN-GLY-FP ------GH-- -------KYP LE-VHNGWFF LPWHRMFIYW HERILAKILD DETFALPYWN
PpaPPO3      RAYQLQKDLP D---SDPRSF TNQAKLHCLY CDS-GIY-YP ------DNP- --------WP LE-IHNGWFF FPWHRMFLYF HERILAKLLD DDTFALPFWN
PpaPPO5      KGYQLLRSLS E---SDPRSL SNQAKLHCLY CDN-GIY-YP ------GM-- -------IWP LE-IHNHWLF LPWHRMFLYF HERILAELLD DDSFALPYWN
PpaPPO13     RGYKLLRELP D---LDPRSL SNQANLHCLY CDN-GIY-YN ------NM-- -------TWP LE-IHNHWLF LPWHRMFLYF HERILAKLLD DDTFALPYWN
BdaPPO5      LALQRMKELP D---DDPRSF KNQAAIHEAY CDG-KYN-VV ARSP--GEPD -------ATK FD-VHFSSIF APWHRMYIYF FEGILGELVG DPAFGLPYWN
BdaPPO4      AGVRMMKALP D---DDPRSF KSQATIHEAY CNF-HYK-FL T-----TTP- -------PVD FD-VHFSSVF APWHRMYIYF YERIIGELTN DPAFALPYWN
SitPPO3      RALAKMKALP A---SDPRSF AAQAAIHEAY CDG-HYR-YG ------GGGG -------DAP FD-VHFSWVF APWHRMYLYF YERILGDLIG DDTFALPYWN
SbiPPO6      EAVRKMRALD K---SNPLSF AAQAAVHESY CDG-HYR-LD ------PSEK -------NRP FD-VHFSWIF APWHRMYIYF YERALGDLIG DDTFALPYWG
ZmaPPO4      EAVGKMRALD G---SNPLSF AAQAAVHQSY CDG-HYR-LD ------PTEK -------NRP FD-VHFSWIF APWHRMYIYF YERALGQLIG DDTFALPYWN
SbiPPO5      EAVAKMKALP E---SNPLSF KAQAAIHQAY CDS-YYS-YH ------RSSS AAKKDGVDPA FD-VHFSWIF APWHRMYIYF YERALGQLIG DDTFALPFWS
ZmaPPO5      EAVAKMKALP E---ENPLSF AAQAAIHQAY CDA-YYK-YD ------PTAK -------DAP FD-VHFSWIF APWHRMYIYF YERALGQLIG DDTFALPFWN
SbiPPO7      EATSKMKALP E---SNPLSF AAQAAIHQAY CDS-YYS-YH RSSSSGGSTT AAAKD--DPT FD-VHFSWIF APWHRMYIYF YERALGDLIG DDTFALPFWN
A. marina    DGVSEMKRLI NTDPEDPRGW ILQAYIHGN- CND-----FT ------DC-- ---------- ---KHSNWYF APWHRSYLYY FEQLIQHFSG NATFALPYWD
```

```
             410        420        430        440        450        460        470        480        490        500
             ....|....| ....|....| ....|....| ....|....| ....|....| ....|....| ....|....| ....|....| ....|....| ....|....|
BdaPPO1      WDV---PE-G -----MRMPV EFA----N-- --------SS SVLYD--PIR NPS--HA-PP KLVDL--DF- ----LGPEK- -------NFT D---------
BdaPPO3      WDV---PE-G -----MRMPV EFA----N-- --A-----SS P-LYD--PVR NPR--HA-PP KVVDL--EF- --VRSSVD-- -------DKF TD--------
SbiPPO1      WDV---PE-G -----MGIPE VFT----D-- --E-----AS P-LYD--PIR EPS--HA-PP KVADLDFSY- ---RLEKNL- -------TD- ----------
OsaPPO1      WDV---PE-G -----MRMPL QFA----N-- --A-----SS P-LYD--QMR NPW--HA-PP KLVDL--DY- ---AMDVVE- -------NNY TD--------
ZmaPPO1      WDV---PE-G -----MRVPP EFA----D-- --A-----SS P-LYD--PIR NPE--HA-PP RVVDL--DF- ---SKVDKNY -------TD- ----------
SitPPO1      WDV---PE-G -----MRVPQ EFA----D-- --E-----VS P-LHD--PSR NPR--HA-PP RVVDL--DF- SYAEKNCTD- ---------- ----------
SbiPPO8      WDV---PE-G -----MRVPP EFA----D-- --A-----AS P-LYD--PMR NPD--HA-PP RLVDL--DF- SYVDRNCTD- ---------- ----------
ZmaPPO2      WDA---PG-G -----MTLPA IYS----S-- --A-----SS P-LYD--ERR NTA--HQ-PP SFLGL--DF- ----SDTDP- -------DDM PR--------
OsaPPO2      WDA---PD-G -----MSFPA MYA----N-- --R-----WS P-LYD--PRR NQA--HL-PP FPLDL--DY- ----SGTDTN I------PK- ----------
BdaPPO6      WDA---PA-G -----MALPS IYN------- ---------- ---------- --------PP FLLGL--HY- ----SGTDD- -------DAS GISR------
SbiPPO3      WDA---PG-G -----MSLPA IYA----N-- --K-----SS P-LYD--ERR DPA--HQ-PP FTVDL--DY- -----DRIE- -------QNI PR--------
ZmaPPO6      WDA---PG-G -----MSLPA IYA----D-- --K-----SS P-LYD--ERR DPA--HQ-PP FTLDL--DY- ----DGTEP- -------TIP R---------
SbiPPO4      WDA---PG-G -----MSMPA IYA----N-- --K-----TS P-LYD--ERR DPA--HQ-PP FTLDL--DY- ----DGTEPT I------PR- ----------
MguPPO1      YDS---PA-G -----MTIPA MYT----N-- --P-----SS P-LYD--KLR NQG--HQ-PP ALIDL--NF- ----SGSGT- -------ITA ----------
BdaPPO2      WDA---PG-G -----MRMPA MYV----D-- --P-----KS P-LFD--PRR DAR--HA-PP ELINL--DY- ----NGREP- -------TFT D---------
SitPPO2      WDA---PD-G -----MRMPA MYA----D-- --------QS SQLFD--PRR DGR--HA-PP KLIDL--DY- ----NGSER- -------RFT D---------
SbiPPO2      WDA---PD-G -----MRMPA MYA----D-- --P-----SS Q-LFD--PRR NSR--HA-PP KLINL--DY- ----NGREPR FF-----TD- ----------
ZmaPPO3      WDA---PD-G -----MRMPA MYA----D-- --R-----SS Q-LFD--PRR DSR--HA-PP KLINL--DY- ----NANVR- -------EP- R-FTY-----
AcoPPO6      WDT---PE-G -----MYMPG MYL----D-- --R-----NS P-LYD--DNR NHN--HF--T ALMDY--DF- ---TWGAPN- -------PT- PEQE------
AcoPPO7      WDT---PE-G -----MYMPG MYL----D-- --R-----NS P-LYD--DNR NHN--HF--T ALMDY--DF- ---TWGAPN- -------PT- PEQE------
AcoPPO1      YDQ---PS-G -----MRFPD IYK----N-- --S--PKDQN P-LFD--CIR NPE--HL-NG ALMDY--RY- ---EFGDPS- -------PT- PEQE------
VviPPO1      WDN---PD-G -----MYMPT IYA----S-- --------SP SSLYD--EKR NAK--HL-PP TVIDL--DY- ----DGTES- -------TIP D---------
VviPPO3      WDN---PD-G -----MYMPA IYA----S-- --------SP SSLYD--EKR NAK--HL-PP TVIDL--DY- ----DGTEPT I------PD- ----------
AcoPPO5      WDA---PG-G -----MRMPT LYA----D-- --------SN SALYD--PLR DAK--HQ-PP VMVDL--DY- ----DLIDP- -------QIS E---------
VviPPO2      WDA---PA-G -----MQMPA MFA----D-- --P-----NS P-LYD--KLR DAK--HQ-PP KLIDL--DY- ----NLTDS- -------NDT N---------
PtrPPO3      WDS---PG-G -----MQLPA MYA----D-- --P-----NS P-LYD--SLR NKN--HQ-PP TLLDL--DW- ----SGTDT- -------PTS N---------
MesPPO1      WDS---PE-G -----MQLPA LYA----N-- --P-----KS P-LYD--HYR NKN--HQ-PP TLVDL--DY- ---NRTENP- -------TSN ----------
RcoPPO1      WDS---PA-G -----MQLPA IYV----D-- --P-----KS P-LYD--QFR NKN--HQ-PP TLVDL--DY- ----NGSEN- -------PTS K---------
PtrPPO11     WDS---PQ-G -----MQIPA FFA----D-- --P-----KS A-VYD--PLR DKS--HQ-PP KIIDL--DF- ----PGVDF- -------PLP D---------
PtrPPO1      WDS---PS-G -----MQMPY IFT----D-- --P-----KS P-LYD--QFR DQN--HQ-PP ILLDL--DY- ---GAGYPN- -------PTN A---------
PtrPPO12     WDA---PA-G -----MQMPA IFT----D-- --P-----KS P-LYD--PLR DAN--HQ-PP ILLDL--NY- ---ATGDAN- -------PD- PAKA------
PtrPPO5      WDA---PA-G -----MQIPA IFT----D-- --P-----KS P-LYD--PLR DAN--HQ-PP TLFDL--NY- ---ATGDAN- -------PD- PAKA------
PtrPPO14     WDA---PA-G -----MQMPA IFT----D-- --P-----ES P-LYD--PLR DAN--HQ-PP TLLDL--NY- ---AKGDAN- -------PD- PAKA------
PtrPPO2      WDA---PA-G -----MQMPA IFT----D-- --P-----KS P-LYD--PLR DAN--HQ-PP TLLDL--NY- ---AKGDAN- -------PD- PAKA------
PtrPPO9      WDA---PA-G -----MQMPA IFT----D-- --P-----ES P-LYD--PLR DAN--HQ-PP TLLDL--NY- ---AKGDAN- -------PD- PAKA------
PtrPPO15     WDA---PA-G -----MQMPA IFT----D-- --P-----ES P-LYD--PLR DAN--HQ-PP TLLDL--NY- ---AKGDAN- -------PD- PAKA------
GmaPPO2      WDN---PA-G -----MRIPP IFT----D-- --K-----SS P-LYD--EHR NSD--HV-NA -FIDL--DY- ---KKDDSP- -------VK- PPQTILWPPV
GmaPPO4      WDN---PA-G -----MRIPP IFT----D-- --K-----SS P-LYD--EHR NSD--HV-NA -FIDL--DY- ---KKDHSP- -------VK- PPQTIIWPPV
GmaPPO3      WDN---PP-G -----MAMPA IYT----D-- --E-----NS P-LYD--PFR NAL--HQ-PP VIVDLANEA- ----NILDP- ---------- ----------
GmaPPO5      WDN---PDGG -----MVIPL IYR----D-- --E-----DS P-LYD--PRR NPD--IT-PD TLVDL--NY- ---GSGKDP- ---------- ----------
GmaPPO6      WDN---PDGG -----MVLPS IFA----D-- --E-----DS P-LYD--PRR NPD--IT-PT TLVDL--NY- ---GSGKEP- ---------- ----------
GmaPPO8      WDN---PDGG -----MVLPS IFA----D-- --E-----DS P-LYD--PRR NPD--IT-PT TLVDL--NY- ---GSGKEP- ---------- ----------
GmaPPO7      WDN---PKGG -----MTIPS FYV----D-- --K-----NS P-LYD--PLR NLN--HQ-PP VLIDL--DY- -NRRDDDDP- -------SAS ESVDP-----
GmaPPO1      WDA---PK-G -----MQLPS IYA----D-- --P-----KS P-LYD--PLR NAN--HQ-PP TLVDF--DF- ----NLDNP- -------IS- ----------
GmaPPO9      WDA---PD-G -----MQLPS IYA----D-- --P-----KS P-LYD--TLR NAN--HQ-PP TLVDL--DF- ----NLEDP- -------IS- ----------
GmaPPO10     WDA---PK-G -----MQLPS IYA----D-- --P-----KS P-LYD--TLR NAN--HQ-PP TLVDL--DF- ----NLEDP- -------IS- ----------
PpaPPO4      WDSMMEPERP -----SQIPE YFY------- --------TM PALVD--PLR NPR--HR-TP AVPDLSTDL- ----TGRSP- -------MDI KNATL-----
SmoPPO6      WDN---QDGG -----SYIPE MFR----R-- --E-----GT P-IFD--ANR NEA--NY-AP ARVDL--GY- ---APGVDPS FGEPTYKTD- ----------
SmoPPO5      WDN---QDGG -----SYIPE MFR----R-- --E-----GT P-IFD--ANR NEA--NY-AP ARVDL--GY- ---APGVDPS FGEPTYKTD- ----------
SmoPPO7      WDN---QDGG -----SYIPE MFR----R-- --E-----GT P-IFD--ANR NEA--NY-AP ARVDL--GY- ---APGVDPS FGEPTYKTD- ----------
SmoPPO8      WDN---QDGG -----SYIPE MFR----R-- --E-----GT P-IFD--ANR NEA--NY-AP ARVDL--GY- ---ASEVDP- -------KSK PNKPD-----
SmoPPO9      WDQ---EG-G -----RYIPD MFR----R-- --------ET A-LCD--AKR NTS--HY-EP TRVDL--IY- ---SPGSDD- -------KSD ----------
SmoPPO4      WDQ---EG-G -----RYIPD MFR----R-- --------ET A-LYD--AKR DTS--HY-EP TRVDL--FY- ---SPGSDK- -------KSD ----------
SmoPPO11     WDQ---EG-G -----RYIPD MFR----R-- --------EI A-LYD--KKR DTS--HY--D TRVDL--IY- ---SGSDGK- -------SD- ----------
SmoPPO10     WDQ---EG-G -----RYIPD MFR----R-- --------ET A-LYD--AKR NTS--HY-EP TRVDL--IY- ---SLGSDK- -------KSD ----------
MguPPO2      WDN---PA-G -----MVMPE RFA----V-- --Q-----DS P-LFN--PRR NQT--HL-PP TPTDL--AY- ----SAKSP- -------TN- P---------
MguPPO5      WDN---PP-G -----MVMPD MFA----V-- --T-----TS P-LYD--ARR NLE--HL-PP AAADL--NF- ----ASKPT- -------TD- P---------
MguPPO6      WDN---PK-G -----MQMPK IFD----N-- --S-----GS P-LYD--ANR NQK--NR-PP AIIDL--GF- ---SGATED- ---------- ----------
MguPPO3      WDN---PK-G -----MTIPP MFL----D-- --E-----KS A-IYN--SKR NQD--NL--K AIVDL--GM- ---TGNKDP- ---------- ----------
MguPPO4      WDN---PK-G -----MTIPP MFV----D-- --P-----KA A-IYD--AKR NPA--NM-PP AVVDL--GL- ---TGNKDP- ---------- ----------
MguPPO7      WDN---PK-G -----MTIPP MFV---ED-- --P-----RS A-LYD--RKR NQK--NL--R AVVDL--GL- ---TDATDT- ---------- ----------
MguPPO8      WDN---PK-G -----MTMPP MFV----D-- --P-----KS S-LYD--KKR NQE--HL-PP AVVDL--GL- ---TNSTDT- ---------- ----------
MguPPO9      WDN---PK-G -----MTIPP AFI----D-- --P-----KS A-LYD--AKR NPD--NM-PP AVVDL--GF- ---TGSTDP- ---------- ----------
GmaPPO11     WDS---VE-G -----MQIPS YFN----N-- --P-----NS S-LYH--QLR NQN--HL-PP HVVDL--NY- ---NKLDPN- -------DDT PS--------
SmoPPO2      WDN---QS-A SDPLPNVIPQ MYL------- --------TN PVLFD--DFR NPD--HF-PP RLADL--NF- ---VPGDGA- -------PIS D---------
SmoPPO3      WDN---QF-A SDPLPNVIPQ MYL------- --------TN PVLFD--DFR NPD--HF-PP RLADL--NF- ---VPGDGA- -------PIS D---------
SmoPPO1      WDN---QAAG -SPLANVIPS AYL----N-- --------QA P-LID--SLR NQQ--HL-PP RLVDL--NYS GQ-DSGPIP- -------PD- ----------
PtrPPO13     WDI---PE-G -----MVIPE MYM------- --------KA P-FFH--EAR DFS--HF-PP SVVDL--DY- ---SCTTPSS -------EDY RCFESGLGP-
VviPPO4      WDN---PD-G -----MVIPE MYV------- --------TG S-LVD--TDR ENS--HL-PP QVVDL--NYN LQ-EKGLGP- ---------- ----------
AmePPO1      WDS---PD-G -----MFIPD MYL------- --------NG S-MID--TER DNS--HL-PP QVADI--NYD YV-ESGLGP- ---------- ----------
AcoPPO4      WDT---PA-G -----MVMPD MYF------- --------QE P-FLD--TER SDK--HIHRP RVVNL--DY- -N-LVEINR- -------TS- ----------
AcoPPO2      WDA---PE-G -----MVTPD MYF------- --------KE P-FID--TER SET--HVRRP KVADI--NY- NFVESNLKP- ---------- ----------
AcoPPO3      WDA---PE-G -----MLTPE MYF------- --------EE P-FID--IER SDN--HVRRP KVADINYNY- ---VDDFNL- -------KP- ----------
PpaPPO9      WDN---QSQG -ATQGNVMPR YYS----E-- --RRWSNKSN P-LYD--PNR NRC--AL-PP HLIDL--NS- ---GGGCSGM -------PD- ----------
PpaPPO12     WDN---QS-P IAPLGNLVPR YMS----E-- --AYWNRRPN P-LYN--RNR NRC--AL-PP FLIDL--NS- ---GVGCVR- -------MSS A---------
PpaPPO2      WDN---QL-P IEPLGNIIPC YFD--EVD-- --------NN P-LYS--PNR AQC--AR-PP NIVDL--AYT GAGLNGECP- -------DM- ----------
PpaPPO10     WDN---QSPG -AVQGNVMPS FFD----D-- --PI----DN P-LYN--ANR DQC--AR-PP NVVDL--GYI SQ-TPGGPC- -------PDS ----------
PpaPPO1      WDN---QS-P IPPFPGEIPK AFA---HEWS LSPNAINKTS S-LYD--PDR NSC--SK-NP QIVEF--QN- ---FTACPL- -------PQN K---------
PpaPPO7      WDN---QS-P VAPYPGAVPR VYD---LEWS LSPDAINKTS S-LYD--ADR NNC--SK-PP RLPEF--HN- ---FTACEP- -------QNL ----------
PpaPPO11     WDN---QDST ----QNPLPN IFP----K-- --------TS P-LFD--PLR NACATQSVPP RLVEF--QD- ---VVGIPCI -------PKN P---------
PpaPPO6      WDN---PE-A TEPLPNAIPS FYW----N-- --P-----NS S-LYD--PNR NIC--SR-PP FIVDL--DS- ---IGGCTN- -------KTA ----------
PpaPPO8      WDN---QS-P EPPFGNTIPE IYA----N-- --P-----KS P-LYD--VNR NNC--SV-PP FVVDM--DT- ---RTGCTN- -------KTS ----------
PpaPPO3      WDN---QT-P EEPYANVLPI SYA----K-- --S-----GS P-LWN--QQR NKC--AE-PP LIIDL--NT- ---IGGCTA- -------KSP ----------
PpaPPO5      WDN---QS-E EEV-ANTLPD IYA----S-- --N-----RS SSLWN--HNR NKC--AQ-QP NIVNL--NT- ---VGGCT-- -------EKT P---------
PpaPPO13     WDN---QS-S SEE-ANILPR IYS----T-- --N-----ET SYLRD--LNR NKC--AQ-PP NLVHL--NS- ---IGGCT-- -------DKT A---------
BdaPPO5      WDA---PA-G -----MMLPA AFT----N-- --P-----DS P-LYD--ANR KPE--NE-RG AFIDL--SL- ---SPKVKS- -------DPN KFKDDL----
BdaPPO4      WDS---PD-G -----MSLPA IFA----N-- --P-----AS P-LYD--AKR NQE--NV--K STIDL--NL- ---GPGKQND LPLCSSTEDG D---------
SitPPO3      WDA---PA-G -----MALPD IFK----D-- --A-----GS P-LYD--AKR NPA--NL--G AYLNL--HI- ---AKAGDT- -------TVI PFDPQAAHLN
SbiPPO6      WDT---PA-G -----MGIPA VFK---GD-- --D-----DN P-LFD--PYR NMD--N--ND ALIDL--DY- ---LKTPRR- -------PTV PFTPPPVSDA
ZmaPPO4      WDA---PA-G -----MGIPA IFKQGVST-- --A-----DN P-LYD--PYR NME--NM-DA L-LDL--DY- ---LKKPRR- -------DTI PFEPP--TDP
SbiPPO5      WDH---PA-G -----MVVPA LFK---GS-- --F-----VN P-LCN--PNR NMA--NV-DA L-VDL--DY- ---LSHKDD- -------KPI DFKGP-----
ZmaPPO5      WDT---PA-G -----MVVPP LFK----D-- ------SMGN P-LYD--PNR NPS--NV--D ALVDL--DY- ---LNDRNA- -------EPI PFKGPRDEKY
SbiPPO7      WDH---PA-G -----MVVPP LFR----DAG --AAGSTASN P-LYD--ANR NPA--NL--D ALIDL--DY- ---LNDKDN- -------EPI PFSG------
A. marina    WSQ---TH-- ------TVPS SFY---GD-- --------AN P-LDDNISIQ SIC--SS-AP ---------- ---TAGRGR- -------PD- ----------
```

```
               510        520        530        540        550        560        570        580        590        600
               ....|....| ....|....| ....|....| ....|....| ....|....| ....|....| ....|....| ....|....| ....|....| ....|....|
BdaPPO1        --------EQ QIQHNLRVMY KQ------MI GN-AALPSLF HGQ------P YRAGQ--ND- ---------- --MP------ ---GAGTVEL APHNTVHTWT
BdaPPO3        --------EQ QIQQNLRVMY KQ------MI SN-AALPSLF HGQ------P YRAGE--SD- ---------- --RP------ ---GAGTVEL FPHNTMHTWT
SbiPPO1        --------EQ QILHNLRVMY KQVLDPSAMI SG-AALPSLF MGQ------P YRAGD--AA- ---------- --KP------ ---GAGTVEL APHNSMHTWT
OsaPPO1        --------DE QIKHNLWIMY KQ------MI SS-APLASLF HGQ------P FRAGE--AS- ---------- --KP------ ---GAGTVEL QPHNLMHVWV
ZmaPPO1        --------EQ QIQLNLRTMY KQ------MV TN-APLPSLF YGQ------P YRAGD--RE- ---------- --MP------ ---GAGTVEL SPHNTIHVWA
SitPPO1        --------EQ QIQLNLRIMY KQ------MV TN-APLPSLF HGQ------P YRAGD--RG- ---------- --MP------ ---GAGTVEL WLHNIVHRWT
SbiPPO8        --------EQ QIQLNLRIMY KQ------MV TN-APLPSLF HGQ------P YRAGD--RA- ---------- --MP------ ---GAGTVEL SPHNVIHRWS
ZmaPPO2        --------DL QVDQNLKVMY RQ------MI SG-AKRRELF MGQ------P YRAGD--AA- ---------- --DP------ ---GAGTVEN VPHGPLHVWV
OsaPPO2        --------DQ LIDQNLNIMY RQ------MI SG-ARKAELF MGQ------P YRAGD--QP- ---------- --EP------ ---GAGTVES VPHNPVHRWT
BdaPPO6        --------DN QIDENLRIMY RQ------MV SN-AKKPQLF LGQ------P YRAGD--DA- ---------- --DP------ ---GAGSIEN VPHGIVHFWT
SbiPPO3        --------AQ QIDQNLMIMY RQ------MI SG-AKKKELF FGL------P YHQGD--QP- ---------- --DP------ ---GAGTVES VPHGPVHFWS
ZmaPPO6        --------AQ QIDQNLMIMY RQ------MI SG-AKKKELF FGM------P YRQGD--QP- ---------- --DP------ ---GAGTVES IPHNPVHFWS
SbiPPO4        --------HQ QIDQNLMIMY RQ------MI SG-AKKKELF FGL------P YHQGD--QP- ---------- --DP------ ---GAGSVEL IPHNTVHFWS
MguPPO1        --------EQ QVKRNLTVMY RQ------MV SN-SKTPRLF FGS------A YRRGE--DP- ---------- --NP------ ---GSGSVEN IPHGPVHVWT
BdaPPO2        --------RQ QVDHNLRVMY RQ------MV SL-SPTPSLF FGG------A YRAGD--EP- ---------- --DQ------ ---GPGPMEN IPHGPVHIWC
SitPPO2        --------NQ QIDRNLRVMY RQ------MV SL-SPTPSLF FGG------A YRAGD--DP- ---------- --NQ------ ---GPGPVEN IPHGPVHIWC
SbiPPO2        --------KQ QVDHNLRVMY RQ------MV SL-SPTPSLF FGS------A YRAGD--EP- ---------- --NQ------ ---GPGPVEN IPHGPVHIWC
ZmaPPO3        --------KQ QVDHNLRVMY RQ------MV SL-SPTPSLF FGS------A YRAGD--DP- ---------- --NQ------ ---GPGPVES IPHGPVHIWC
AcoPPO6        --------EE VKIRNLKKIH NM------FT ET-IGAPSLF LGG------P LSAGQ--VP- ---------- --QD------ ---IAGALEN Q-HGLPHQWT
AcoPPO7        --------EE VKIRNLKKIH NM------FT ET-IGAPSLF LGG------P LSAGQ--VP- ---------- --QD------ ---IAGALEN Q-HGLPHQWT
AcoPPO1        --------ES VILENQKYLY NM------FA EN-LFNPGNF MGQ------P ISEGQ--TL- ---------- --N------- ---APGTLEN L-HAIPHRFA
VviPPO1        --------DE LKTDNLAIMY KQ------IV SG-ATTPKLF LGY------P YRAGD--AI- ---------- --DP------ ---GAGTLEH VPHNIVHKWT
VviPPO3        --------DE LKADNLAIMY KQ------IV SG-ATTPKLF FGY------P YRAGD--AI- ---------- --DP------ ---GAGTLEL VPHNIVHKWT
AcoPPO5        --------QE QININLSIMY RS------VV SN-GKTAQLF LGS------P YRAGD--EP- ---------- --DP------ ---GPGSFEN VPHGTVHLWA
VviPPO2        --------EQ QISSNLSIMY RQ------IV SS-AKTTSLF MGA------A YRAGD--EP- ---------- --DP------ ---GQGTLEN IPHGPVHIWC
PtrPPO3        --------EE QLSSNLTIMY RQ------MV SN-GKTPRLF FGG------A YRAGD--EP- ---------- --GP------ ---EPGPIEN IPHGPVHIWT
MesPPO1        --------QA QISSNLTIMY RQ------MI SN-GKTAKLF MGS------S YRAGD--EP- ---------- --DP------ ---GAGSIEN IPHGPVHIWT
RcoPPO1        --------QE LLSSNLTIMY RQ------MV SN-GKTARLF HGS------A YRAGD--EA- ---------- --DP------ ---GAGSIEN IPHGPVHVWC
PtrPPO11       --------PV QVASNLNVMY RQ------VV T--AKYPTLF MGR------P YRAGD--EP- ---------- --EP------ ---GAGSLED VPHTTVHIWT
PtrPPO1        --------NQ LYSSNLTVMY KQ------MV SG-AAKPTLF FGK------S YRAGE--DT- ---------- --SP------ ---GAGTIET TPHNNIHRWT
PtrPPO12       --------EE LYASNLNVMY RQ------MV SG-ATKPTLF FGK------P YRAGD--DP- ---------- --SP------ ---GMGTIET TPHTEIHFWT
PtrPPO5        --------EE LYASNLNVMY RQ------MV SG-ATKPTLF FGK------P YRAGD--DP- ---------- --SP------ ---GMGTIET TPHTQIHIWT
PtrPPO14       --------EE LYASNLNVMY RQ------MV SG-ATKPTLF FGK------P YRAGD--DP- ---------- --SP------ ---GMGTIET TPHTQIHYWT
PtrPPO2        --------GE LYASNLNVMY RQ------MV SG-ATKPTLF FGK------P YRAGD--DP- ---------- --SP------ ---GMGTIET TPHTQIHYWT
PtrPPO9        --------EE LYASNLNVMY RQ------MV SG-ATKPTLF FGK------P YRAGD--DP- ---------- --SP------ ---GMGTIET TPHTQIHYWT
PtrPPO15       --------GE LYASNLNVMY RQ------MV SG-ATKPTLF FGK------P YRAGD--DP- ---------- --SP------ ---GMGTIET TPHTQIHYWT
GmaPPO2        EKKNNSIVDN LIVNNLIKVY TA------VA SKTNSSPDYF LGP------A FEAGS--AP- ---------- --QQ------ ---HFGSLES L-HNTVHSWT
GmaPPO4        VKKNNSIVDN LIVNNLIKVY TA------VA SKTNSSPDSF LGP------A FEVGS--DP- ---------- --KK------ ---HFGSLES L-HNTVHSWT
GmaPPO3        --------SK QIVNNEITMF RH------VV IE-GISPSLF LGK------P YYAGC--EP- ---------- -NNP------ ---GAGSLEK GPHNNVHRWT
GmaPPO5        ---------- SVEQNLGVMY TS------VV SG-AKRASLF HGK------A FFAGK--QP- ---------- --EL------ ---GGGTVEL GPHTALHRWT
GmaPPO6        ---------- SVEQNLGVMY TS------VV SG-AKRASLF HGK------P FLAGK--QP- ---------- --EL------ ---GGGTVEL GPHTAVHRWT
GmaPPO8        ---------- SVEQNLGVMY TS------VV SG-AKRASLF HGK------P FLAGK--QP- ---------- --EL------ ---GGGTVEL GPHTAVHRWT
GmaPPO7        --------NE QIASNLSIMY RN------VI SN-GKLPRLF LGS------P YRAGD--EP- ---------- --EP------ ---GAGSLEN VPHGPVHSWT
GmaPPO1        --------NG RISTNLTIMY RQ------LV SN-GKTPTLF LGN------P YRAGD--AP- ---------- --DP------ ---GGGSVEG VPHGPVHLWT
GmaPPO9        --------NG KISNNLTIMY RQ------VV SN-GKTPTLF LGN------P YRAGD--EP- ---------- --DP------ ---GFGSVEN VPHGSVHLWT
GmaPPO10       --------NG KISNNLTIMY RQ------VV SN-GKTPTLF LGN------P YRAGD--EP- ---------- --DP------ ---GFGSVEN VPHGPVHLWT
PpaPPO4        --------EE VIESNNNLLY QT------VV SG-ATLPSLF LGK------P LRGGV--EK- ---------- -TSL------ ---GASAFEV GPHGAIHGWV
SmoPPO6        --------DQ IRQDNISVMY NN------VA K--VKLQDAF FGA------P IRKGN--NN- ---------- --G------- ---GDGSLEK APHTAVHVFG
SmoPPO5        --------DQ IRQDNISVMY NN------VA K--VKLQDAF FGA------P IRKGN--NN- ---------- --G------- ---GDGSLEK APHTAVHVFG
SmoPPO7        --------DQ IRQDNISVMY NN------VA K--VKLQDAF FGA------P IRKGN--NN- ---------- --G------- ---GDGSLEK APHTAVHIFG
SmoPPO8        --------DQ IRQDNISIMY NN------VA K--VNNQEAF FGA------P VRKGN--NN- ---------- --G------- ---GDGSLEK TPHTAVHIFG
SmoPPO9        --------EQ IREDNLSIMY NN------VA K--VKQPDAF FGH------P IRKGS--GS- ---------- --KN------ ---GPGGVER APHNAVHIFG
SmoPPO4        --------EQ IREDNLSIMY NN------VA K--VKQPDAF FGY------P LRKGS--SS- ---------- --IN------ ---GPGGVER APHNAMHIFC
SmoPPO11       --------EQ IREDNLSIMY NN------VA K--VKQPDAF FGY------P IRKGS--SS- ---------- --VN------ ---GPGGVER APHNALHIFC
SmoPPO10       --------EQ IREDKLSVMY NN------VA K--VKQPDAF FGY------P LRKGS--SS- ---------- --IN------ ---GPGGVER APHNAVHIFC
MguPPO2        --------KK IIPNNLTIMY GE------MV RN-VKKLEDF YGA------K YVVGT--KP- ---------- --DP------ ---GPGSVER GSHTAMHAWV
MguPPO5        --------SK IVTNNLTTMY GE------MV RN-VSKLEDF YGA------K YVVGT--AP- ---------- --DP------ ---GPGTVER GSHTALHVWV
MguPPO6        --------LQ VVVNNLTVMY SE------MI RS-VNSTLDF MGQ------P YKAGD--AA- ---------- --SP------ ---GMGSSER GSHVAAHVWV
MguPPO3        --------LQ VVSNNLTVMY SE------MI RG-NADVYDF MGQ------P YREGT--AV- ---------- --NP------ ---GPGSSER GSHTAIHVFV
MguPPO4        --------LQ VVSNNLTVMY SE------MV RA-NSDIYDF MGK------P YREGT--LV- ---------- --NP------ ---GPGASER GSHVAIHVFV
MguPPO7        --------LQ VVSNNLTIMH NE------MI RG-ISDPLDF MGA------R YSEGS--DP- ---------- --NP------ ---GEGSVER GSHTSIHAWV
MguPPO8        --------LQ VVANNLTIMY SE------MI RG-NSDANDF MGQ------A YREGD--ES- ---------- -SAK------ ---AAGSSER GSHTSVHAWV
MguPPO9        --------LQ VVSNNLTVMY TE------MV RG-NSDVYDF MGK------A YREGT--PV- ---------- --NP------ ---GPGSSER GSHTAIHAFV
GmaPPO11       --------HQ QVSYNLAFMY KQ------MV L--ASTKELF MGS------P FRLGD--NP- ---------- --TP------ ---GMGSIEA APHNTVHTWV
SmoPPO2        --------SK QREENNALMY KQ------LV N--ARTTRLF YGK------E YHPGD--SK- ---------- --PD------ ---GLGTIES APHNTVHKWT
SmoPPO3        --------SK QREENNALMY KQ------LV N--ARTTRLF YGK------E YHPGD--SK- ---------- --PD------ ---GLGTIES APHNTVHKWT
SmoPPO1        --------ST QRAENNALMF QQ------IV SG-SRTPSLF FGQ------A YRAGD--AN- ---------- --AP------ ---GGGTIEN APHGTVHVWT
PtrPPO13       --------ED QVHTNLVMMY NQ------MV AG-AKKMELF MGC------P YKAGE--GG- ---------- -SCN------ ---GPGTIEL APHNTVHKWV
VviPPO4        --------EE QIKINMALMY TQ------MV SG-AKTTELF MGC------P YKGGE--GG- ---------- -FCE------ ---GPGTIEL APHNTLHDWT
AmePPO1        --------ED QISTNIAFMY NQ------MV SG-AKKTELF MGC------P YKAGE--LG- ---------- -FCD------ ---GPGTIEL APHNALHTWV
AcoPPO4        --------EE QISHNLAVMY HQ------VV SG-AKKMELF MGC------I YKSGI--NG- ---------- -SCD------ ---GAGTVEQ APHDSLHGWV
AcoPPO2        --------EE QISKNLAFMY HQ------MV SG-AKKVELF MGC------T YKPGT--NN- ---------- -SCD------ ---GPGTVEL APHNALHSWV
AcoPPO3        --------DE QISKNLAFMY HQ------MV SG-AKKVELF MGC------T YKRGT--TG- ---------- -FCN------ ---GPGTIEV APHNALHGWV
PpaPPO9        --------DY LRTENNRLMY NQ------MV SG-PTTPSLF FGS------T YRFGW--SG- ---------- --GM------ ---GGGTLEG APHGTVHIFT
PpaPPO12       --------RS ILTENNRLMY TQ------MV SG-ATTPSLF HGS------T YRFRW--MG- ---------- --GM------ ---GGGTLEG APHGTVHIFT
PpaPPO2        --------EG LQDSNGEIMR LQ------MS ---AETARLF HGN------P YRYNT--RG- ---------- --GR------ ---SPGNMER TPHGNVHVFA
PpaPPO10       --------LG LDTYNNDIMR LQ------MA ---AETARLF HGA------P VRYNT--RG- ---------- --GR------ ---SPGNVER TPHGNVHIFT
PpaPPO1        --------SA VREQNAQLMW TQ------AV SV-SVTPLIM AGA------P YRFGD--DG- ---------- --GM------ ---GMGGIEG RPHGPVHVWT
PpaPPO7        --------TL AREQNAQLMW TQ------AV SV-SVTPLIM SGA------P YRFGD--YG- ---------- --GM------ ---GIGGIEG RPHGPVHLWV
PpaPPO11       --------EA VRLENAHLLW TQ------FL SG-GPTPLTF HGA------P YRFGD--YG- ---------- --GV------ ---GMGTLEA RPHGPVHIWT
PpaPPO6        --------DF LRVQNTRLQY TQ------MV LG-GDTPALF YGM------P YRLGD--FG- ---------- --GG------ ---GPGTFED QPHGTIHAWV
PpaPPO8        --------DF LRVQNTRLTY SQ------IV LG-GGTPSLF YGL------P YRKGD--FG- ---------- --GA------ ---GSGVFED QPHGTIHAWV
PpaPPO3        --------SV LRTENDRIMY TQ------VV SG-AMSPQLF YGM------P YRYGD--AG- ---------- --GH------ ---GPGTFED NPHGTVHLWV
PpaPPO5        --------TE LRIENTRLMY TQ------IV SG-APTPRLF FGQ------A YSYGD--GG- ---------- --GH------ ---GPGTFED NPHGTVHLWV
PpaPPO13       --------DE LRIENTQVMY TQ------IV TG-APTPRLF FGE------P YSYGD--SG- ---------- --GY------ ---GPGTFED NPHGTVHLWV
BdaPPO5        --------LG LIDSNLCAMY RQ------MN ---VKEPVDF HGG-----YP SRTTK--PP- ---------- ---------- ----VGSLET GAHTAAHIWV
BdaPPO4        --------SC VKAANLCTVY RQ------MA ---VDTAAQF HGD------R FCAAG--VK- ---------- ---------- ---APGSLEN AAHTAVHIWT
SitPPO3        --------NQ VVQNNLATLY VQ------MM R--NKKAQDF LGG------K FCSSY--PGT ---------- -RSSG----- ---TSGSLES MAHTSVHVWT
SbiPPO6        AARQAY--DD AVRTNLATVY IQ------QI RD-GKGPRAF LGE------K LCSEA--SSR VKEVNERSKR RQGAGAPKVK ASNAQGTLER MAHTTVHVWA
ZmaPPO4        AARAKY--DD AVQTNLCTIY LQ------QV RD-GKGPRAF LGEKLCSESS FRVKEINERS KRRQADGQSS KVSPSKSQ-- ---SQGTLER MAHTTVHVWT
SbiPPO5        -KDEKY--KD LVNRNLCTIY TQ------QV R---KGPESF LGE------K YCTAI--EG- ---------- --RG------ ---SAGTLES MAHTAMHVWV
ZmaPPO5        --------KE LVNKNLCTVY TQ------QI RS-GKGAESF LGE------K YCTDI--GS- ---------- -STS------ ---SMGSLER MAHTAVHVWV
SbiPPO7        --PKDEEYKK LVNKNLCTVY TQ------QV RK-GAES--F LGE------K YCTDL--GS- ---------- -STS------ ---SMGSLER MAHTAVHVWV
A. marina      --------GE EITQQELEEY VG-------- ---PVRIESI QQN------P DYATY--GG- ---------- --IG------ ---GSGELEV IPHNFIHRWV
```

```
                 610        620        630        640        650        660        670        680        690        700
           ....|....| ....|....| ....|....| ....|....| ....|....| ....|....| ....|....| ....|....| ....|....| ....|....|
BdaPPO1    GD-----IT- ---------- ---LP---N- --------VE NMGDYYSAGR DPIFYPHHNN IDRLWQAWRD ---AGVARGY RGHVDFT-DP DWLDSSFL-F
BdaPPO3    GD-----LA- ---------- ---RP---S- --------VE NMGVYYSAGR DPIFYPHHNN IDRLWEVWRD ---VGAARGY RGHVDFT-DP DWLDSSFL-F
SbiPPO1    GD-----NS- ---------- ---RP---N- --------AE NMGVYYSAGR DPLFYPHHGN IDRLWECWRR IA-TGNRT-- --HEDFT-DP DWLDSSFL-L
OsaPPO1    GDL----LS- ---------- ---YP---N- --------AE DMGAYYAAGR DPIFYTHHAN IDRLWDVWRS ---NGKGE-- ----DFT-DP DWLDSSFL-F
ZmaPPO1    GD-----LS- ---------- ---QP---N- --------HE NMGTYYSAAR DPIFFPHHTN SDRLWEVWRG ---GPARHA- ----DFT-DP DWLDSSFL-F
SitPPO1    GD-----LS- ---------- ---RP---N- --------HE DMGAYYSSAR DPIFYPHHAN SDRLWEVWRR DD-AGAGGDR PRHADFT-EP DWLDSSFL-F
SbiPPO8    GD-----LS- ---------- ---RA---N- --------HE NMGAYYSAAR DPIFYPHHAN SDRLWEVWRG -V-GGGHA-- ----DFT-DP DWLDSSFL-F
ZmaPPO2    GD-----PT- ---------- ---QP---N- --------GE DMGNFYSAAR DPVFFAHHGN IDRLWHVWRG LS-PRNT--- ----DFA-DA DWLDASFH-F
OsaPPO2    GD-----PR- ---------- ---QP---N- --------GE DMGIFYSAAR DPVFFAHHGN VDRMWHIRRG LLFPGDT--- ----DFT-DP DWLDASFF-F
BdaPPO6    GD-----PR- ---------- ---EA---N- --------QE DMGNFYSAAR DPVFFAHHGN IDRLWHVWRS QIHNNNNGGF ----DFA-DP EWLDAAFL-F
SbiPPO3    GD-----PS- ---------- ---QP---N- --------NE DMGNFYSAAR DPLFFAHHGN IDRLWSVWNG LR-AGNT--- ----NFT-DP DWLNASFL-F
ZmaPPO6    GD-----PR- ---------- ---QP---N- --------GE DMGNFYSAAR DPLFFAHHGN IDRMWSVWNG LR-PGNS--- ----DFT-DP SWLDASFL-F
SbiPPO4    GD-----PR- ---------- ---QP---N- --------GE DMGNFYSAAR DPLFFAHHGN IDRLWSVWNG LR-AGNT--- ----NFT-DP DWLNASFL-F
MguPPO1    GD-----PT- ---------- ---QP---N- --------GE NMGNFYSAGR DPIFYAHHSN IDRLWSLWKG -L-GGRRQ-- ----DIT-DP DFLDASFL-F
BdaPPO2    GD-----PN- ---------- ---QP---A- --------GE DMGNFYSAGR DPLFYAHHGN IDRMWSVWKG LD-PRRHR-- ----DLT-DP DWLDSSFL-F
SitPPO2    GD-----PS- ---------- ---QP---S- --------RE DMGNFYSAGS DPLFYAHHAN IDRMWTVWKG -L-DDVRRRR T---DLA-DP DWLDASFL-F
SbiPPO2    GD-----PN- ---------- ---QP---H- --------GE DMGNFYSAGF DPLFYAHHAN IDRMWSVWKG -L-DARRHT- ----DLT-DP DWLNASFL-L
ZmaPPO3    GD-----PN- ---------- ---QP---D- --------GE DMGNFYSAGF DPLFYAHHAN IDRMWSVWKG LD-VRRHT-- ----DLT-DP DWLDASFL-L
AcoPPO6    GP-----EA- ---------- ---IP----- --------RH DMGNFYTAAR DTMFFGHHAN VDRLWDIYSD LR-GNKV--- ----EFD-NP DWLDASFI-F
AcoPPO7    GP-----EA- ---------- ---IP----- --------YH DMGNFYTAAR DTIFFGHHAN VDRLWDIYSD LR-GNKV--- ----EFD-NP DWLDASFI-F
AcoPPO1    SQ-----VE- ---------- ---RP----- --------RW HMGNFHTAAR DPVFYAHHAQ IDRLWTIYKE -R-RAITTP- ----EFP-TD EWLEASFI-F
VviPPO1    GL-----AD- ---------- ---KP----- --------SE DMGNFYTAGR DPIFFGHHAN VDRMWNIWKT -I-GGKNRK- ----DFT-DT DWLDATFV-F
VviPPO3    GL-----AD- ---------- ---KP----- --------SE DMGNFYTAGR DPIFFGHHAN VDRMWNIWKT -I-GGKNRK- ----DFT-DT DWLDATFV-F
AcoPPO5    GD-----RT- ---------- ---QP---N- --------NE NMGNFYSAGR DPIFFCHHSN IDRMWTIWKG -L-GGKRK-- ----DFT-DP DWLNAGFL-F
VviPPO2    GD-----RT- ---------- ---QP---N- --------IE DMGNFYSAAR DPIFFSHHSN VDRMWTVWKG -L-GGKRK-- ----DFT-DP DWLDAGFV-F
PtrPPO3    GD-----NT- ---------- ---QP---N- --------SE DMGNFYSAAR DPIFFCHHSN VDRMWSVWKT -L-GGRRT-- ----DLT-DP DWLNASFF-F
MesPPO1    GD-----NT- ---------- ---QP---N- --------LE NMGNFYSAGR DPIFFAHHSN VDRMWTIWKT -L-GGKKRT- ----EFT-DP DWLDASFL-F
RcoPPO1    GD-----NT- ---------- ---QP---N- --------LE DMGNFYSAAR DPIFFAHHSN VDRMWTIWKT -L-GGNKRT- ----EFT-DP DWLDAEFI-F
PtrPPO11   GD-----AD- ---------- ---QP---N- --------RE NMGVFYAAAR DPIFFAHHGN IDRLWEVWKK LP-GGKRE-- ----NFT-DP DWLDAAFL-F
PtrPPO1    GD-----PT- ---------- ---QE---N- --------NE DMGNFYSAAR DPIFFCHHSN VDRMWTIWKT IP-GGTRR-- ----DIS-DP DWLNSEFL-F
PtrPPO12   GD-----PN- ---------- ---QT---N- --------GE NMGNFYSAGR DPIFFCHHSN VDRMWDLWKK IP-GGKRK-- ----DFK-DP DWLNSEFL-F
PtrPPO5    GD-----PN- ---------- ---QT---K- --------GE NMGNFYSAGR DPIFYCHHSN VDRMWDLWKK IP-GGKRK-- ----DIE-DP DWLNSEFL-F
PtrPPO14   GD-----PN- ---------- ---QT---N- --------GE NMGNFYSAGR DPIFYCHHSN VDRMWDLWKK IP-GGKRE-- ----DIE-DS DWLNSEFL-F
PtrPPO2    GD-----PN- ---------- ---QT---N- --------GE NMGNFYSAGR DPIFYCHHSN VDRMWDLWKK IP-GGKRK-- ----DIE-DP DWLNSEFL-F
PtrPPO9    GD-----PN- ---------- ---QT---N- --------GE NMGNFYSAGR DPIFYCHHSN VDRMWDLWKK IP-GGKRK-- ----DIE-DP DWLNSEFL-F
PtrPPO15   GD-----PN- ---------- ---QT---N- --------GE NMGNFYSAGR DPIFYCHHSN VDRMWDLWKK IP-GGKRK-- ----DIE-DP DWLNSEFL-F
GmaPPO2    GE-----RE- ---------- ---NN----- --------HH DMGLLATAAK DPIFFAHHSN VDRMWNIWKT ELLDGRRF-- ----DHK-SD DWLESSFF-F
GmaPPO4    GE-----LE- ---------- ---KP----- --------NH DMGLLATAAR DPIFFAHHSN VDRMWNIWKT ELLDGRRF-- ----DHK-SD EWLESSFF-F
GmaPPO3    GD-----PT- ---------- ---QP---H- --------RI DMGYLAWAAR DPIFFAHHSN VDRIWNIWKT IP-GGFRR-- ----DFN-DD DWLESSFL-F
GmaPPO5    GD-----PR- ---------- ---QP---N- --------KE DMGRFYSAGR DPIFYSHHAN VDRMWNIWKT IP-TGKRR-- ----DFK-NR DWLETTFF-F
GmaPPO6    GD-----PR- ---------- ---QP---N- --------KE DMGRFYSAGR DPAFYSHHAN VDRMWNIWKT IP-SGKRR-- ----DFK-NR DWLETSFF-F
GmaPPO8    GD-----PR- ---------- ---QP---N- --------KE DMGRFYSAGR DPAFYSHHAN VDRMWNIWKT IP-SGKRR-- ----DFK-NR DWLETSFF-F
GmaPPO7    GD-----KR- ---------- ---EP---N- --------RE NMGIFYSAAR DPIFFSHHAN VDRMWSIWKT -I-P------ ---------- ---ESAFF-F
GmaPPO1    GD-----IN- ---------- ---QP---N- --------IE NMGDFYSAAR DPIFYSHHSN VDRMWSIWKT -L-GGKRR-- ----DFT-DS DWLESGLL-F
GmaPPO9    GD-----IN- ---------- ---QP---N- --------IE DMGTFYSAAR DPIFFSHHSN IDRMWSIWKT -L-GGKK--- ----DFT-DS DWLESGFL-F
GmaPPO10   GD-----IN- ---------- ---QP---N- --------IE NMGTFYSAAR DPIFYSHHSN IDRMWSIWKT -L-GGKRR-- ----DFT-DS DWLESAFL-F
PpaPPO4    GD-----PA- ---------- ---QP---N- --------GE DMVHLYSAAR DPIFYAHHAN CDRMWCDWKN LP-GGKRT-- ----GFEDDP DWMESQLL-F
SmoPPO6    GS-----PN- ---------- ---NP---N- --------GE DLGNFYSAGK DPLFYCHHGN VDRMWDVWRS -L-GNQ---- ----DFT-DA DYLNTEFL-F
SmoPPO5    GS-----PN- ---------- ---NP---N- --------GE DLGNFYSAGK DPLFYCHHGN VDRMWDVWRS -L-GNQ---- ----DFT-DA DYLNTEFL-F
SmoPPO7    GS-----PN- ---------- ---NP---N- --------GE DLGNFYSAGK DPLFYCHHGN VDRMWDVWRS -L-GNQ---- ----DFT-DA DYLNTEFL-F
SmoPPO8    GS-----PK- ---------- ---NP---N- --------GE DLGNFYSAGK DPLFYSHHAN VDRMWDVWRS -L-GNH---- ----DFR-DA DYLNTEFL-F
SmoPPO9    GS-----PY- ---------- ---QP---N- --------FE DMGNFYSAGR DPLFYSHHGN VDRMWDVWRS -L-GNK---- ----DFT-DS DYLNTEFL-F
SmoPPO4    GS-----PN- ---------- ---QP---N- --------NE DMGNFYSAGK DPLFYCHHGN VDRMWDVWRS -L-GNM---- ----DFT-DP DYLNTEFL-F
SmoPPO11   GS-----PK- ---------- ---EP---G- --------YE DMGNFYSAGR DPLFYCHHGN VDRMWDVWRS -L-GNK---- ----DFT-DP DYLNTEFL-F
SmoPPO10   GS-----PD- ---------- ---EH---N- --------NE DMGNFYSAGR DPLFYCHHGN VDRMWDVWRS -L-GNK---- ----DFT-DP DYLNTKFL-F
MguPPO2    GE-----NT- ---------- ---AS----- --------GE DMGNFYSTGR DPLFYSHHTN VDRLWTIWRN QR-SGPKQPK ----DFP-DS DWLNADYL-F
MguPPO5    GE------N- ---------- ---TP---T- --------GE DMGNFYSAGR ESLFYSHHAN IDRLWSTWQG -L-RGKKPK- ----GFK-DK DWLDATYL-F
MguPPO6    GD-----PR- ---------- ---NK---Y- --------SE DMGNFYSAGR DVLFYCHHAN VDRMWSVWRT LK-TDVPK-- ----DIK-DP DYLNAAFL-F
MguPPO3    GD-----PR- ---------- ---EP---S- --------GE DLGNFYSAGR DSLFYSHHCN VDRMWTLWQY FL-PSNKVPD K---KIT-DP DFLNASFL-F
MguPPO4    GD-----PR- ---------- ---EP---S- --------GE DLGNFYSAGR DPLFYCHHAN VDRMWTLWQY FL-PNNKLID K---KIT-DP DYLNASFL-F
MguPPO7    GD-----PE- ---------- ---QP---S- --------GE DLGNFYSAGR DPLFYCHHAN VDRMWYLWQR -L-PNNKLPD K---RIT-DP DYLNASFL-F
MguPPO8    GDF----KD- ---------- ---QP---S- --------GE DMGNFYSAGR DPLFYSHHAN VDRMWTLWQY FL-PSNKVPD K---RIT-DT DFLNTAFL-F
MguPPO9    GDY----KN- ---------- ---QP---A- --------GE DMGNFYSAGR DPLFYSHHSN VDRMWTLWQF FL-PSNKIPD K---KIT-DP DFLNTAFL-F
GmaPPO11   GA-----AD- ---------- ---KP---H- --------HE DMGAFYTAAR DPIFYAHHPN SDRLWGLWKT LE-GGRK--- ----DYSDDP DWLDSEFY-F
SmoPPO2    GS-----SG- ---------- ---KP---H- --------FE DMGVFYSAAR DPIFYPHHAN LDRLWTVWKT LP-RNRKGPF RPRRDYK-DP DWLNATFL-F
SmoPPO3    GS-----SA- ---------- ---KP---H- --------FE DMGVFYSAAR DPIFYPHHAN LDRLWTVWKT LP-RNRKGPF RPRRDYK-DL DWLNATFL-F
SmoPPO1    GS-----AA- ---------- ---AP---N- --------RE DMGTLYSAAR DPIFFAHHGN IDRLWVVWKT LP-GGRRR-- ----DPT-DT DWLDAKFL-F
PtrPPO13   GS-----NL- ---------- ---NP---GS --------RE DMGVFYSAAR DPIFYPHHAN IDRLWDVWRT LH-GNK---- ----VIT-DP DWLDSSFF-F
VviPPO4    GT-----GL- ---------- ---NP---G- --------RE DMGSFYSAGR DPIFYGHHGN VDRLWEVWRR LQ-WEYYNS- ----EIT-DT EWLDSYFF-F
AmePPO1    GS-----NL- ---------- ---NT---E- --------RE DLGAFYSAAR DPIFYAHHSN IDRLWNVWTD -L-RGNNKP- ----AIE-DP EWLDSTFY-F
AcoPPO4    GK-----ES- ---------- ---NR---Y- --------LE HMGVFYAAAR DPIFFAHHAN LDRIWNVWEE LR-NNEP--- ----EIV-DP DWLDSYFF-F
AcoPPO2    GK-----ES- ---------- ---NR---Y- --------LE NMGVFYAAAR DPIFYAHHSN IDRLWGVWKE LR-NNEP--- ----EIV-DP DWLESYFY-F
AcoPPO3    GK-----DS- ---------- ---NP---Y- --------LE NMGSFHSAAR DPIFYAHHSN IDRLWGVWKE LR-NNVP--- ----EII-DP DWLDSYFY-F
PpaPPO9    GN-----PYS R--------- ---NP----- --------YD DMGNLNVAAL DPLFYAHHAN IDRLWEVWKG -M-GGRHK-- ----DIS-DP DYLKSQFV-F
PpaPPO12   GN-----PYA K--------- ---NP----- --------YD DMGNLNVAAL DPLFYAHHAN IDRLWEVWKG -M-GGRRR-- ----DLT-DP DYLRSQFI-F
PpaPPO2    GD-----PL- ---------- ---ADLRLNP DTQQKYAAYD DMGNLNRAAY DPLFYAHHTN LDRLWDFWIK -Q-GGAHT-- ----DFS-DP DYLNSKFI-F
PpaPPO10   GD-----PS- ---------- ---ANTRVNP LTLKPYAVRD DMGNLNRAAV DPLFYSHHAN LDRLWAFWIA -Q-GGMHS-- ----DYN-DP DYLNSKFI-F
PpaPPO1    NT-------- ---------- ---------- ---------K DMGTFKDSAA DPIFFSHHAN IDRLWEAWKT -L-PGKYRK- ----DVT-DP DYLNTAFT-F
PpaPPO7    NE-------- ---------- ---------- ---------K DMGVFKDSAS DPIFFAHHAN MDRLWEIWKT -F-PGKYRK- ----DVT-DP DYLNTALT-F
PpaPPO11   NV-------- ---------- ---------- ---------I KMSTNKDSAS DPIFFSHHAN VDRLWEMWKT -L-PGKYRK- ----DIS-DP DYLNTEFT-F
PpaPPO6    GDPNAEAPF- ---------- ---RP----- --------FD DMGNFGRSAF DPVFYAHHAN VDRLWCLWTH LR-GGKR--- ----TYPMDS DFVTSQFS-F
PpaPPO8    GSVDPLPGR- ---------- ---RP----- --------GD DMGNFGRSAF DPVFYAHHTN TDRLWELWLT KI-PGGQRT- ----HPT-DP DFLNSEFT-F
PpaPPO3    GN-----PD- ---------- ---SE---SG M-------FD DMGNFGRAAR DPIFYAHHSN IDRIWTVWNT -L-PGEQRK- ----APV-DP DFLDARFT-F
PpaPPO5    GD-----PD- ---------- ---AP---NK --------FD DMGNFGRAAR DPIFYPHHAN IDRIWTIWKT -M-PGKQRM- ----EPN-HT DFLDASFT-Y
PpaPPO13   GD-----PD- ---------- ---AA---TA --------FN DMGNFGRSAR DPVFYTHHSN IDRIWTIWKT -L-PGKQRT- ----EPT-HA DFLDSRFT-Y
BdaPPO5    GE-------- ---------- ---------- ---------- HMGNLKTAAR DPVFYSNHSN VDRMWHLWST KL-GNHE--- ----DLT-ND QWLNTNFV-F
BdaPPO4    GD-----PL- ---------- ---SA---NA --------GE DMGVLGTAAR DPVFYSHHSN VDRLWHLWRS TD-PGHA--- ----DFVGDE DWLQTSFV-F
SitPPO3    GD-----PG- ---------- ---SS---TT GHDGQKHSLA DMGFLATAAR DPVFYSHHAN VDRMWHLWST KL-GRR---- ----NFD-DP EWLDTSFV-F
SbiPPO6    GRSSPKAPAT CTVDGGGVVA HDGKP---HC --------NN DMGFLGTAGR DPLFYSHHAN VDRMWHLWST KL-GGK---- ----GFD-DA EWLDTSFV-F
ZmaPPO4    GRAN---PAT CSAEQGGVVG HDGKP---HC --------QV DMGFLGTAGR DPLFYSHHAN VDRMWHIWST RL-GGK---- ----GFD-DP EWLDTSFV-F
SbiPPO5    GK-----PTT KPCDASTGGV LSHKDGAFNC --------NN DMGFLGTAGN DPVFYSHHAN VDRMWHLWST RL-GGGQ--- ----GIT-DT DWLDATFV-F
ZmaPPO5    GK-----AGP TPSSEACSAA TGGFP---NH TKGG-YSCNN DMGFLGSAGR DPLFYSHHSN VDRMWHLWST RL-GGGQ--- ----GIT-EA DWLDTSFV-F
SbiPPO7    GK-----AGP TPATATCSEA SGGVP---NH SKGGVYSCNN DMGFLGTAGR DPLFYSHHAN VDRMWHLWST RL-GGGQ--- ----GFT-DT DWLDASFV-F
A. marina  GD-----VG- ---------- ---GK----- --------NS NMVQFFSPL- DPIFWMHHCN IDRLYSDWLS -R-PGHL--- ----PPS-ED EWQNQSFNEF
```

```
               710        720        730        740        750        760        770        780        790        800
        ....|....| ....|....| ....|....| ....|....| ....|....| ....|....| ....|....| ....|....| ....|....| ....|....|
BdaPPO1    YNE--DARLV --RITVRDVL DTEK-LRYTH AGV---GMPW LDAKPPTTPN V--------- ---------- ---------- -N-----TKK GSLKSVRFP-
BdaPPO3    YDE--EARLV --RITVRDVL DIDK-LRYAY DGV---GTPW LDAKPPATPN V--------- ---------- ---------N TK-----KG- -LLKSVRFPV
SbiPPO1    YDE--EARLV --RITVRDVL RTEK-LRYTY GGV---GLPW LHARPPTTAG V--------- ---------- -------NPK AK-----GGG RLEESVRFP-
OsaPPO1    YDE--EARLV --RITVRDVL DMDK-LRYTY RGV---GLPW LDARPPTTPN V--------- ---------- ---------K YR-----VKN RVEKPVMFP-
ZmaPPO1    YDE--DARLV --RVTVRDML DIGR-LRYAY AEV---GLPW LSARPPINPG D--------- ---------- --------VN RG-----RDP PHLESVRFP-
SitPPO1    YDE--EARLV --RVTVRDML DIGK-LRYTY AEV---GTPW LGARPPVNPD L--------- ---------- ---------- SR-----RSR QHLKPVRFP-
SbiPPO8    YDE--DARLV --RVTVRDML DVGR-LRYAY AEV---GLPW LSARPPVVAD V--------- ---------- --------NR GR-----GGP THLKSVRFPV
ZmaPPO2    YDE--EARLV --RARVRDCL DPAA-LRYAY QDV---GLPW LDARPARTAG A--------- ---------- -----GAPAP AT-----D-- ----GVVFPA
OsaPPO2    YDE--EARLV --RVRVRDCL DPSA-LRFTY QDV---GLPW LNAKPSTGAA S--------- ---------- ---------- ---------T PAPAAGAFP-
BdaPPO6    YDE--EARLV --RVRVRDCL DMAS-LGYAY QEV---PLPW LDARPTKPAS ---------- ---------- ---------- -------PGT PARALDALP-
SbiPPO3    YDE--EARLV --RVRVRDCL DTAA-LGYSY QDV---ALPW LNARPTTETG S--------- ---------- ---------- ---------- PVPAAGALP-
ZmaPPO6    YDE--EARLV --RVRVRDCL DTAA-LGYAY QDV---ALPW LNAKPAKEAG S--------- ---------- ---------- -------PAP TAGALP----
SbiPPO4    YDE--EARLV --RVRVRDCL DTAA-LGYTY QDV---ALPW LNAKPTTETG S--------- ---------- ---------- -------PAP AAGALP----
MguPPO1    YDE--NGQMV --RVKTRDCL DNTK-LGYTY QDV---EIPW LKSRPTPKVS S--------- ---------V LKKLKKLGKA KA-----AAA ETHRPPKEVL
BdaPPO2    YDE--TPKLV --RIRVRDVL DTDR-LRYRF QDV---PMPW TTARPTVTPR A--------- ---------- ----RSFGTP TA-----VAA SAKKATKFP-
SitPPO2    YDE--TPKLV --RIRVRDVL DTGA-LGYQY QDV---PMPW RAARPTVTAA A--------- --------AT RRADSLLTPA AQ-----AAS AAKKARKFP-
SbiPPO2    YDE--TSKLV --RIRVRDVL DTDR-LRYRY QDV---PLPW TAARPTVTAA A--------- -------RRA DSLLAPAAQA AT-----AAA AAKKAGEFP-
ZmaPPO3    YDE--TPKLV --RIRVRDVL DTDR-LRYRY QDV---PLHW TAARPTVTAG T--------- ---------R RADSFLAPAA QA-----APA AARKAGEFP-
AcoPPO6    YDE--NRQVV --NCKVRDCL ITEN-LGYTY TTE---RHAW KDIKRTYKKL Q--------- ---------- ---RGKKRSA GE-----GLS LIPVSEFGSA
AcoPPO7    YDE--NRQMV --NCKVRDCL ITEN-LGYTY TTE---QHAW KDIKRTYKKL Q--------- ---------- ---RGKKRSA GE-----GLS LIPVSEFGST
AcoPPO1    YDE--NRNVV --KCKVRDCL DPTQ-LRYMY QTD---EKAW LNIRRSYYTK K--------- ---------- ---KAAPRSA GA-----D-E LVAISEFGSE
VviPPO1    YDE--NKQLV --KVKVSDCV DTSK-LRYQY QDI---PIPW LPKMTKAKAK T--------- ---------T TKSSKSGVAK AA-----ELP KTTISSIGDF
VviPPO3    YDE--NKQLV --EVKVSDCV DTSK-LRYQY QDI---PLPW LPKNTKAKAK T--------- ---------T TKSSKSGVAK AA-----ELP KTTISSIGDF
AcoPPO5    YDE--NKQLV --RVKVRDCL DQKN-LRYTY QEV---DIPW LKTRPTAGKA K--------- ---------- APVAPKKASP RS-----LNS REESSNIFP-
VviPPO2    YDE--NAQLV --RVKVRDCL DPTK-LRYAY QEV---DIPW LKSRPKPPNA ---------- ---------- ---------- -------AEI PSSSSIVFP-
PtrPPO3    YDE--NANPV --RVKVRDCL DSRN-LGYVY QDV---EIPW LQSRPTPRRS A--------- --------KK VASNIFGHEK EAIAAEKKKN ALTPITAFP-
MesPPO1    YDE--NANLV --RVKIRDCM DNKK-LGYVY QEV---DIPW LKSKPTTRRS M--------- -----EKVAT VSHHHGQVAQ AA-----ETK NLSPISSFP-
RcoPPO1    YDE--NANPV --RVKVRDCI DTKK-LGYIY QDV---DIPW LKSRPKSRRL IKKVAN---- ---------K VFHHDGQAAH AA-----ETL NLPPITAFP-
PtrPPO11   YDE--NANLV --RVKIRDCL DTTK-LRYGF QDV---ASPW INARPKRKPN K--------- ---------- ---------P KP-----TVP VDTADPTKPI
PtrPPO1    YNE--NAELV --RCKVSDCL DNTG-LRYTY QNV---EIPW LESKPIPRRL G--------- ---------- -------KKA AE-----TKT ALTPITAFP-
PtrPPO12   WNE--NKELV --RVKVKDTL DTKK-LRYGF QDV---PIPW LKTRPTPKLT R--------- ---------- ---QEKSRRS AK-----KSV VLTPISAFP-
PtrPPO5    WDE--NKELV --RVKVKDTL DTKK-LGYGF QDV---PIPW LTTRATPKLT R--------- ---------- ---QEKSSRA AE-----KSV VLTPISAFP-
PtrPPO14   WDE--NKELV --RVKVKDTL DTKK-LGYGF QDV---PIPW LTTRATPKLT R--------- ---------- ---QEKSRRA AE-----KSV VLTPISAFP-
PtrPPO2    WDE--NKELV --RVKVKDTL DTKK-LRYGF QDV---PIPW LKARATPKFT R--------- ---------- ---QEKSRRS AK-----KSV VLTPISAFP-
PtrPPO9    WDE--NKELV --RVKVKDTL DTKK-LRYGF QDV---PIPW LKTRATPKLT R--------- ---------- ---QEKSRRA AK-----KSV VLTPISAFP-
PtrPPO15   WDE--NKELV --RVKVKDTL DTKK-LRYGF QDV---PIPW LKTRATPKLT R--------- ---------- ---QEKSRRA AK-----KSV VLTPISAFP-
GmaPPO2    YDE--NKNYV --RVKVKDCL DSKK-MGYDY QRV---DLPW LLAGELIKPK K--------- ---------- ----EIILLR SK-----PEA STFKTLQLP-
GmaPPO4    YDE--NSNYV --RVKVKDCL DSKK-MGRR- ------LFPF V-----QNQK L--------- ---------- ---------- ---------- ----------
GmaPPO3    YDE--NKNLV --RVKIKDCL DSRK-LGYDY EYV---DDTPW LNVRPRPKYA V--------- ---------- ---------- -------PLP LPFPLPQ-PL
GmaPPO5    YDE--NKNLV --SVKVKDSL DSKK-MGYVY QDV---DIPW LQKRTKPKRT R--------- ---------- KAKQVAFTQQ FGGIGAAIAA ETGPSSKFPL
GmaPPO6    YDE--NKTLV --RVKVKDSL DTNK-MGYVY QDV---AIPW LEKKPKKRT R--------- ---------- KAKKVAFAQQ FGGIGAAMAA ETGPSSKFPL
GmaPPO8    YDE--NKTLV --RVKVKDSL DTNK-MGYVY QDV---AIPW LEKKPKPKRT R--------- ---------- KAKKVAFAQQ FGGIGAAMAA ETGPSSKFPL
GmaPPO7    YDE--NKNLV --RVKVKDSL DTRK-LGYVY QDV---DIPW LKNKPKPKRS K--------- ------AKKL ALAQDFIVGA AQ-----AVE TTPKSVKFP-
GmaPPO1    YDE--NKNLV --RVKVKDCL DTRK-LGYVY QDV---EIPW LKSKPSPRRS R--------- -------VQK VALGPHFNTG VA-----RAA ETSRNVQFP-
GmaPPO9    YDE--NKNLV --RVKVKDCL DERK-LGYVY QDV---DIPW LNSRPTPRRS R--------- -------VQK VALAQNFGVG AA-----RAA ETSRNVKFP-
GmaPPO10   YDE--NKNLV --RVKVKDSL DTRK-LGYVY QDV---DIPW LNSKPTPRRS R--------- -------VQK VALAQNFGVG AA-----HAA ETSRNVKFP-
PpaPPO4    YDE--NADLI --RVKIKDSL DIVK-LRYTY EEV---DNPW AHHDSLRFKK H--------- ---------G KSLSSSVAAT GL-----SSI ILKQNVDKPV
SmoPPO6    FDE--NQDLV --RVKVKDSI DNQTKLKYKY ASMP-QDKVW INYKPLPLAK T--------- ---------- ---------- -------AFA QPKSSIVASL
SmoPPO5    FDE--NQDLV --RVKVKDSI DNQTKLKYKY ASMP-QDKVW INYKPLPLAK T--------- ---------- ---------- -------AFA QPKSSIVASL
SmoPPO7    FDE--NQDLV --RVKVKDSI DNQTKLKYKY ASMP-QDKVW INYKPLPLAK T--------- ---------- ---------- -------AFA QPKSSIVASL
SmoPPO8    FDE--NQDLV --RVKVKDAI DNKTKLKYEY ASMP-QDKVW IDYRPLPLAK T--------- ---------A FAQPVASLVP GATAMAAEYP LVRSSSQNAE
SmoPPO9    FDE--NQDLV --RVKVKDSL NSEKMLKYKY QAMP-DDKSW TAYEPKPLKQ T--------- ---------- -----PWSGS SY-----EYR LVKASAENPK
SmoPPO4    FDE--KQDLV --RVKVKDSL NNEKMLKYKY QAMP-DDETW TAYEPKPLKE T--------- ---------- -----PWSGS SY-----EYR LVKASAENPE
SmoPPO11   FDE--NQDLV --RVKVKDSL NNEKMLKYKY QAMP-DDKSW IAYEPKPLKQ T--------- ---------- -----PWSGS SY-----EYR LVKASAENPE
SmoPPO10   FDE--NQDLV --RVKVKDSL NNEKMLKYKY QAMP-DDKSW TAYEPKPLKQ T--------- ---------- -----PWSGS SY-----EYR LVKASAENPE
MguPPO2    YDE--NARLV --RVKNSDTL ENEK-MGYVY QEV---DLPW LKNRPAARVT K--------- ---------- -------SKV AA-----TTA APPPETVFP-
MguPPO5    YDE--NKNLV --QVNVKDAL DNEK-MGYTY QKV---DNPW VNYRPKPKVK K--------- ---------- ------SKAA KN-----SKA PTATDTIFP-
MguPPO6    YDE--NKQLV --RVKVGDCL DHKK-MGYEF EKV---DTPW LNYKPTKKAA K--------- ---------- ------AKIL KI-----AAG AETVEALFP-
MguPPO3    YDE--NKQLV --RVSVKDSL DNLK-MGYEY ERV---DLPW LDYRPPPQAA A--------- ---------- -----ARITR TS-----ASA APKAETLFP-
MguPPO4    YDE--NAQLV --RVTVKDCL DNTK-LGYDF ERV---DLPW LDYRPPPQTA Q--------- ---------- ------AKVM RT-----VST TRKADAVFP-
MguPPO7    YDE--NAQLV --KVYVRDTF DNRR-MGFDY ERI---DLPW MDYRPPLQAA R--------- ---------- --------TK IN-----KVD APNAKTLFP-
MguPPO8    YDE--NAQLV --RVTVKDCL DNLR-MGYDF ERI---DLPW LDYRPPRQSA R--------- ---------- -----AKINR AS-----STA APKAAALFP-
MguPPO9    YDE--NAQLV --RVTVKDCL DNLK-MGFEY ERV---DLPW LDYRPPAQAA S--------- ---------- -----AKVTR AG-----KTT VKVEDTVFPI
GmaPPO11   YDE--NANFV --RVKVRDCL DTKK-LGYVY EDV---DLPW LRTPPTSPKS K--------- ---------- -----LLREA NK-----SPL LSSKPSKFP-
SmoPPO2    YDE--KRRLV --RVRVRDAL KTEQ-LGYRY QDI---HNAW VTARPSTAVS G--------- ---------K IQKLESVFST KV-----KRP LKNKLTRLNK
SmoPPO3    YDE--KRRLV --RVRVRDAL KTEQ-LGYRY QDI---HNAW VTARPSTVAG A--------- ---------- ---------- ------KSKP LVSASPDSP-
SmoPPO1    YDE--NSRLV --RVRVRDAL TTEQ-LGYRY QDT---GNAW ASARPTIVAG A--------- ---------- ---------- -------RNL VSNAA-----
PtrPPO13   YDE--KLQLN --RIKIRDVL SITK-LQYAY EKV---DFTW LNNRPKPSVP L--------- ---------E VARNILRMRQ NAKQQQLQRN DILSSNFSPH
VviPPO4    HDE--KRQLV --RIKFQDVV NITR-LRYAY EEV---ELPW LNARPRPSVP P--------- -------KIA RDILKMRDLD HENNQELPSS PFLSADFGPH
AmePPO1    HNE--KLELV --RIRIRDVL DMSK-LRYTY EKV---DNVW LNAKPKPSVP P--------- ---------K IARQILKMRE NENPFLDSSN QAIVLPELGL
AcoPPO4    HNE--KSQLV --RIKVRDVL NMTK-LRYGY EKV---DNLW LNARPKPSMD P--------- ---------- TMARHALNTK HN-----GYL TAQSNDFRPN
AcoPPO2    HNE--KSQLV --RIKIRDVL NITK-LGYDY EKI---DHPW LNARPKPSMD P--------- ---------- TIARHALNMR HN-----GYL TTQSSDFTPK
AcoPPO3    HNE--KSQLV --RIKIRDVL NTTK-LGYEY EQI---DNPW LNARPKPSMD PAIARHALNT R--------H NAHQHMNSEL TSNGFVSGYL TAQSRDFRPK
PpaPPO9    YDE--NKRLV --SVSVGQVL NAGN-LRYGY QKV---PNPW MNPKAGGMST A--------- ---SVDGMDM SGQSVEATQC SALANSDVPA LIKSSPLLKK
PpaPPO12   YDE--NKRLV --SVSVRQVL SLRN-LRYGY QRV---ANPW MNPRAFGMGV M--------- ---SSDDMDM SGASMEAAKC SSLQYSDVSA LVKASPLLEN
PpaPPO2    YDE--KQNLV --SIQVSQVL DLDK-LRYKY DDM---INPW LMEPPLDPPD EIAPSPSITI AEAFA--TEP SPGPTADPTT CS-----PTF GPDVSRIIRY
PpaPPO10   YDE--NKTLI --SISVSQVL NLAN-LRYDY DQG---DNLW TIEPPLDPPL APAAAPVTIA SIMSF----E SRDESPPPPP SGPRICSPPN SPRLGRMIKN
PpaPPO1    YDE--DGDQV --SVTVAQAL DLDL-LRVKY QEL---PMAW TDKGTVTEGS K--------- --------PT WSFCNPKSSE SD-----IAE MVKNTPRFKE
PpaPPO7    FDE--DGDQV --SVTVAQAL NLDL-LRVKY QEV---PVAW ANKGAVQEGA D--------- --------PS WRFCNPHSSD AD-----IAA MINRTRRFKG
PpaPPO11   YDE--DGEQV --SVCVKQAL DLDL-LRYKY TNS---SADW VENGAVSKAA G--------- --------PQ WKHCNPNATA AD-----DDA LIAATPAFNG
PpaPPO6    YDH--KGNLV --KMNVSQTL DTDL-LRYKF EDS---PTPW ETNGVAAGNE F--------- ---------N IPRCNPASKS AV-----NRL IAATPKHSSK
PpaPPO8    YDE--DANLV --KMKVSQVL DSRK-LRYKY AEM---PTPW ITNGLEAGKE H--------- ---------T VPHCNPLTKS QV-----KSL IEDTRVSKGN
PpaPPO3    FDE--NGDLI --IVNVSQTL DNEL-LRFRY EQM---PTGW VTNGQLPGEE N--------- ---------S VPLCYRGNPT KT-----RKL IADTPYFKPS
PpaPPO5    YDE--NADRV --TVKVSQII NTTL-LRYEY EES---PTAW ITNGQKAGYE N--------- ---------S IPVCNPMNPS QT-----NAM IIATPELTIQ
PpaPPO13   YDE--NADQV --IVNLSQII NTPL-LRYEY EES---PTAW VSRGQKPGHE N--------- ---------N VTACNPLSPS QT-----NAM IYTTPELAAA
BdaPPO5    YDE--TKRPV --RIAVRDVL DAEKQLGYTY EEK--KSLEW MERRIKPAAI K--------- ---------- ---------I DD-----RTR DAKDNSSLPL
BdaPPO4    YDE--KRRPV --RIRVRDVL EPGN-LGYAY EAR--ERLEW LDKRPKPATG I--------- ---------- ---------- DR-----PTV KPSDVVSFPL
SitPPO3    YDE--RPRPV --RIRVRDVL DAAA-LGYSY DER--EPLRW MGARPAPLLA N--------- ---------- ------KGAA AA-----RST MRRAVPAFPL
SbiPPO6    YDDVKSPRLV --RMKFRDVL DVTN-LGYTY DKESEAALPW LKXQAHPVLV R--------- ---------Q DAVVVGAGGG AQ-----GGV GVPADADEGG
ZmaPPO4    YDDYRSPRLV --RMKFRDVL DATR-LGYTY DKESEAALPW LNSKPTRFSG G--------- ---------- --------GK AK-----AKA APKVASEFPL
SbiPPO5    YDDVKSPRKV --RIKFRDVL DTRD-LGYTY DAEYDRDLPW LRPKITKLVP H--------- ---------- ------GKES GA-----AAR SSAAAPVFPL
ZmaPPO5    YDDVKSPRKV --RIRFRDVL DTRD-LGYTY DAESDKDLPW LRCKISSLVP H--------- ---------- -----GKDSP PR-----SSS ARKAAPVFPL
SbiPPO7    YDDVESPRKV --RVKFRDVL DTRD-LGYTY DAEADKDLPW LRPKITSLVP H--------- ---------- -------GTD DS-----GAA KAAAAPVFPL
A. marina  YDV--DKNQV GSEFTCGQTV DSRV-MGYVY STD---QITN LTQTLAARSS I--------- ---------- ------IRQQ VV-----ESV IESKSILVEN
```

```
                 810        820        830        840        850        860        870        880        890        900
             ....|....| ....|....| ....|....| ....|....| ....|....| ....|....| ....|....| ....|....| ....|....| ....|....|
BdaPPO1      ---VSLDAAV SAE-VRR--P ---------- -----RVLRS ---RHEKMAQ EEVLV----- --------VE GVETNG--NE LVKFDVFVNA MEHEK--VE-
BdaPPO3      ---SLDDVAV TAE-VRR--P ---------- -----RVLRS --RREKE-VQ EEVLV----- --------ID GIETDG--AD MVKFDVYVNA -VEYEK-VE-
SbiPPO1      ---VALDAAV TAE-VRR--R ---------- -----RRPRA --QEKQAAA EEVLV----- --------VE GIEADA--GD FVRFDVYVNA -RDYHR-VP-
OsaPPO1      ---VSLGNVV TAE-VRR--P L--------- -----MLWRQ ----PKGATQ EEVLV----- --------VE HIQTDG---- VCKFDVFVNA -REHKK-IE-
ZmaPPO1      ---VSLDAPV TAEVSRR--P ---------- -----GKPRS --------HW EEVLV----- --------VE GIKADG--AD FVKFDVYVNA VDHEK--IM-
SitPPO1      ---VSLDAAV SAA-VTRTRP GN-------- -----TRSRR --------HE VEVLV----- --------VE GIEAHG--GD FVRFDVYVNA VEHEK--VS-
SbiPPO8      SLDAAVTAAV SRRRQGK--P ---------- -----RGQRE ---------- EEVLV----- --------VD GIEADG--AD FVKFDVYVNA -VDHEK-VG-
ZmaPPO2      ---RL-DRTV RVA-VAR--P ---------- -----RVSRS --QEEKD-EE EEVLV----- --------VD GIQVADH-LR YVKFDVFVNQ CAGGDA-S--
OsaPPO2      ---ATLDKTV RVA-VTR--P ---------- -----RASRS ---REEKEEE EEVEI----- ---------- ---PDH--ST YVKFDVFVNA PESGDG-AA-
BdaPPO6      ---ATLDKTV RVA-VAR--P ---------- -----RASRS --AKEKE-EQ EEVVV----- --------VE GIQVSDC-SR FVKFDVLVNV PDGDAE-SG-
SbiPPO3      ---ATLNQTV RVA-VTR--P ---------- -----RTSRT ---RQEKDAE EEVLV----- --------VE GIEVADHFSR FVKFDVFVNE CQGGGG-MG-
ZmaPPO6      ---ATLNQTV RVA-VTR--P ---------- -----KTSRT --RREKD-AK EEVLV----- --------VQ GIEIADHSSR FVKFDVFVND ---SQSGGGM
SbiPPO4      ---ATLNRTV RVA-VTR--P ---------- -----RTSRT --RQEKD-AE EEVLV----- --------VE GIEVADHFSR FVKFDLFVNE SQSGGG-MG-
MguPPO1      P--AKLDKVL KIM-VKR--P ---------- -----KKKRS --KKEKD-EL EEILI----- --------IQ GIELER--DV YAKFDVYIND -EDDET-ST-
BdaPPO2      ---IMLDSAT SVT-VRR--P ---------- -----VSSKR --SKLEKSAK EEVLV----- --------IG GIEVDM--DI AAKFDVFVNA GDDHAA-VG-
SitPPO2      ---VTLDEAM SVT-VKR--P ---------- -----VAARR --SEAEKASE EEVLV----- --------VD GIEVDR--DV AAKFDVFVNA -EDHGA-VG-
SbiPPO2      ---ITLDEAT SVT-VKR--P V--------- -----AVQRS --KAEKA-SK VEVLV----- --------ID GIEVDR--DV AAKFDVFVNT -EDHAA-VG-
ZmaPPO3      ---ITLDEAT IVT-VKR--P V--------- -----AGKRS --KAEKD-SK EEVLV----- --------ID GIEVDR--DV AAKFDVFVNT -EDHGA-VG-
AcoPPO6      P--RALNSTI RVL-VPR--P ---------- -----KVSRS --TDEKE-EF SEVVV----- --------VE GIKFGH--GE STKFDVYIAK -PIEGL-VG-
AcoPPO7      P--RALNSTI RVL-VPR--P ---------- -----KVSRS --TDEKE-EF SEVVV----- --------VE GIKFGH--GE STKFDVYIAK -PIEGL-VG-
AcoPPO1      P--RLLDTTI RVL-VTR--P ---------- -----KASRT --QEEKD-EA AENIF----- --------ID DFTFDH--TD YAGFDVFVTK -ATEGL-AT-
VviPPO1      P--KALNSVI RVE-VPR--P ---------- -----KKSRS --KKEKE-DE EEVLL----- --------IK GIELDR--EN FVKFDVYIND -EDYSV-SR-
VviPPO3      P--KALNSVI RVE-VPR--P ---------- -----KKSRS --KKEKE-NE EEVLL----- --------IK GIELDR--EN FVKFDVYIND -EDYSV-SK-
AcoPPO5      ---RALDSVV KVK-VAR--P ---------- -----KKSRS --KKEKE-DE EEILV----- --------IE GIEYDR--DS MVKIDIYIND -EDETT-TN-
VviPPO2      ---IKLDSTL RVD-VPR--P ---------- -----RKSRS --KKEKE-DE EEVLV----- --------IK GIELER--DK FVKFDIFIND -EDDPV-SR-
PtrPPO3      ---LVLNKVI SVK-VAR--P ---------- -----KKSRS --KKEKE-DE EELLV----- --------IQ GLEFDK--TK ALKFDVYIND -EDDSL-SA-
MesPPO1      ---LILDKVI STV-VKR--P ---------- -----KKSRS --KEDKE-EE EEILV----- --------VD GIELER--DA AVKFDVYVND -EDDSP-SG-
RcoPPO1      ---LVLDKVI STV-AKR--P ---------- -----KKSRS --KKEKE-EE EEVLL----- --------ID AIEFER--NA LVKFDVYVND -EHDSTSAR-
PtrPPO11     G--LL-NKTV SVV-VQR--P ---------- -----NKKRS --KKDKD-EA EEVLV----- --------IE GIEYRI--DL YIKFNVLIND -VPDTP-GK-
PtrPPO1      ---LVLDKTI VTV-VSR--P ---------- -----KKSRS --RKEKE-EE DEVLV----- --------IE GIEYDK--GK FVKFDVFIND -DVEMP-SK-
PtrPPO12     ---VVLDKVI SVE-VSR--P ---------- -----KKSRS --ATEKE-DE DEVLV----- --------IE GIEYEE--NQ LIKFDVLVND -EPDSP-GG-
PtrPPO5      ---VVLDKVI SVE-VSR--P ---------- -----KKSRS --ATEKE-DE DEVLV----- --------IE GIEYEE--NQ LIKFDVLVND -EPDSP-GG-
PtrPPO14     ---VVLDKVI SVE-VSR--P ---------- -----KKSRS --ATEKE-DE DEVLV----- --------IE GIEYEE--NQ LIKFDVLVND -EPDSP-GG-
PtrPPO2      ---VVLDKVI SVE-VSR--P ---------- -----KKSRS --ATEKE-DE DEVLV----- --------IE GIEYEE--NQ LIKFDVLVND -EPDSP-GG-
PtrPPO9      ---VVLDKVI SVE-VSR--P ---------- -----KKSRS --ATEKE-DE DEVLV----- --------IE GIEYEE--NQ LIKFDVLVND -EPDSP-GG-
PtrPPO15     ---VVLDKVI SVE-VSR--P ---------- -----KKSRS --ATEKE-DE DEVLV----- --------IE GIEYEE--NQ SIKFDVLVND -EPDSP-GG-
GmaPPO2      ---LPLESIE RTN-VKR--P ---------- -----KPRSR NENEEEEEGV EEVLV----- --------ID -VEYDS--TD GVRFDVFIND QGDNE--IG-
GmaPPO4      ---------- ---------- ---------- ---------- ---------- ---------- ---------- ---------- ---------- ----------
GmaPPO3      PFPLTLNSIQ RTT-VKR--P ---------- -----KKEES WSEVEKA--- -EVLV----- --------ME -VEYDM--TE DVKFDVFIND -QGDDE-IG-
GmaPPO5      ---TLLDSKV TLL-VKR--P ---------- -----KQLRS --KKDKE-ED EEVLV----- --------ID GIEFDG--DD DVKFDVYITD -EDVEE-IG-
GmaPPO6      ---TLLDSKV TLL-VKR--P ---------- -----KQLRS --KRDKE-EE EEVLV----- --------ID GIEFDG--DD DVKFDVYITD -EMSRD-IG-
GmaPPO8      ---TLLDSKV TLL-VKR--P ---------- -----KQLRS --KRDKE-EE EEVLV----- --------ID GIEFDG--DD DVKFDVYITD -EDVED-IG-
GmaPPO7      ---LTLDSKV STL-VKR--P ---------- -----KLLRS ---KEEKEEE EEVLV----- --------ID GIEFDR--NK AVKFDVFIND -EDDKV-IT-
GmaPPO1      ---LVLDSVV SIV-VKR--P ---------- -----KKSRS --KKEKE-EE EEVLV----- --------IE GVEYDS--NI PVKFDVLIND -EDDKQ-IQ-
GmaPPO9      ---LVLDSVV STM-VKR--P ---------- -----NKSRS --RKEKE-EE EEVLV----- --------IE GIEFER--NT PVKFDVFIND -EDDKQ-IR-
GmaPPO10     ---LVLDSVV STM-VKR--P ---------- -----NKSRS --KKEKE-EE EEVLV----- --------IE GIEFER--NT PVKFDVFIND -EDDKQ-IR-
PpaPPO4      T--KCGERTR DISFTNN--A FHVYIPLGSV NAATKRRGLF GLFQSSSVVV EEILI----- --------LE NIQSPT--GK KISFKIFMDI -DADDT-TS-
SmoPPO6      PV-KVGAGPL TIT-VAQ--P ---------- -----KGSAS ---------- DELLV----- --------LE EMVVGM--QN NTNFNVFINL PEANET-TT-
SmoPPO5      PV-KVGAGPL TIT-VAQ--P ---------- -----KGSAS ---------- DELLV----- --------LE EMVVGM--QN NTTFNVFINL PEANET-TT-
SmoPPO7      PV-KVGAGPL TIT-VAQ--P ---------- -----KGSAS ---------- DELLV----- --------LE EMVVGM--QN NTNFNVFINL PEANET-TT-
SmoPPO8      PV-KVGAGPL TVT-VAQ--P ---------- -----KGSAS ---------- DELLV----- --------LE EMVIGM--QN NTNFNVFINL PEANES-TT-
SmoPPO9      AL-EVGVEPV TIT-VDQ--P ---------- -----KGSAC ---------- DEILV----- --------LE GMEINM--QS PISFNAFINL PEANKN-TR-
SmoPPO4      AL-EVVAEPV TIT-VDQ--P ---------- -----KGSGS ---------- DETLV----- --------LE GMEIDM--RS AISFNAFINL PEANKN-TP-
SmoPPO11     AL-EVRTEPV TIT-VDQ--P ---------- -----KGSAC ---------- DEILV----- --------LE GMEIDM--QL PMSFNAFINL PEANKN-TR-
SmoPPO10     AL-KVEAEPV TIT-VDQ--P ---------- -----KGSAC ---------- DEILV----- --------LE DMEIDM--RS AISFNAFINL PEANKN-TR-
MguPPO2      ---VKLDKVV KVL-VSR--P ---------- -----KKSRS --KKEKE-SE EELLV----- --------IE GIEVDT--AK FVKFDVFVND EDDEAA-DE-
MguPPO5      ---VKLDKVV KVL-VDR--P ---------- -----RKSRS --KKDKE-DE EELLV----- --------IE GIEVDN--SK FVRFEVFIND -EDDKP-EDL
MguPPO6      ---LNLKKIT RVL-VNK--S ---------- -----AKGKA ---------- DEVLV----- --------LE NIETDT--TK FIKFDVFVND -EDDGS-VE-
MguPPO3      ---LKLDRVV RFE-VQK--S ---------- -----KKGKA ---------- DELLV----- --------LE NITVDT--TK FLKFDVFVND -EDDGA-TE-
MguPPO4      ---LKLDGIA RVL-IQK--T ---------- -----KKGKA ---------- DELLV----- --------IE DITVDT--SK FLKFDVFVND EDDN---VGE
MguPPO7      ---LKLDRVV RFQ-VDK--T ---------- -----KKGKA ---------- DEILV----- --------LE NITVDA--SK LLKFDVFVND -EDDNP-SE-
MguPPO8      ---LTLDGVV RFQ-VDK--T ---------- -----KKGKA ---------- DESLV----- --------LE DIKVDT--TK LLKFDVFVND -EDDNP-GE-
MguPPO9      ---VL-DKIV RVL-LPK--T ---------- -----KKGKA ---------- DELLV----- --------IE NIVVDT--SK FLKFDVFVND -EDDNV-AE-
GmaPPO11     ---LVLDSIT STV-VKR--P ---------- -----KKLRS ---KEEKEQE EEVLV----- --------IE GIEFGS--DK YVKFDVHIDD -DEDNL-SE-
SmoPPO2      ALGPLFRMFS YVPLNNL--P TLFSG----- -----GGGDK SDDDDDDDEV EEILV----- --------IQ GVELDY--NK ATKFDVYINY PNANEN-TP-
SmoPPO3      ---------- ---------P ---------- -----TAAAD ---VIEEFGE AEILV----- --------IQ GVELDY--NK ATKFDVYINY PNANEN-TS-
SmoPPO1      ---------- ----VDS--P A--------- -----KLRIG KKEEEEDDEE EEVLV----- --------IQ GVEIDC--DR ATKFDVYINL PGADEN-TS-
PtrPPO13     G--RFLDATL RTR-VNR--P ---------- -----KVRRT --KKEKE-EE EEILV----- --------VH GIDIPE--ER YVKFDVYVNV --VNETIMN-
VviPPO4      G--RTLDTTI RVK-VHR--P ---------- -----KKHRS --KKQKDFKE EEVMV----- --------VY GIEVKE--DS YVKFDVYVNA VDETLM-VG-
AmePPO1      NG-KRLDTTI RVK-VHR--P ---------- -----KTHRS --KIEKE-EE EEVLV----- --------VY GIEIKK--DM YVKFDVYVNA -VDETI-IG-
AcoPPO4      G--RTLDSTI TAK-VQR--P ---------- -----KNQRK ------AENE EEILV----- --------VY GIDTKE--DV YIKFDVFVNA VDAIT--IG-
AcoPPO2      G--RTLDSTI TVK-VHR--P ---------- -----KNQRK ------TENE EEILV----- --------VY GIDIKE--DV YVKFDVYVNA IDATT--IG-
AcoPPO3      G--RTLDSTI TVK-VHR--P ---------- -----KRQRK --TE----DE EEVLV----- --------VH GIDVKE--NV YVKFDVYVNA -INTSI-IG-
PpaPPO9      SGNILFTTPL TFR-VRRLAP ---------- -----TGNRQ ---------- -EILE----- --------IN NIEIDW--TK KAKINAFLFY PGASG--SS-
PpaPPO12     SGNVLSIEPL TFR-VRRLDP ---------- -----TGSWQ ---------- -EILV----- --------IE NIAIDW--TQ TAKINAFLFH PSASG--SS-
PpaPPO2      A--PPLRKNG TQF-STT--P L--------- -----TFRVR --RLDTTGKG FEVLT----- --------FS DLVMDW--TE PAQIHAFLFL -PNATA-AS-
PpaPPO10     A--KLLKKGW TVF-SGE--P L--------- -----TFRAR --RIDPSGNS FEYLT----- --------IN DLVLDW--TK PAQIHAFLFL -PSATE-YS-
PpaPPO1      PV-WLKEGPV TFKLKRR--P ---------- -----WLSDR --------HG EELLQ----- --------IK A-NFPW--NS SHLINVFAFL PDANYETSS-
PpaPPO7      PV-WVQQDGV TFKLRRQ--P ---------- -----WMSEH --------LG EEMLE----- --------IK G-NFNW--SS NHALNAFAYY PDANYERTS-
PpaPPO11     FL-FVKSAPT TFKLKRE--P ---------- -----YRSEL --------HG EELLE----- --------LK G-KLDW--HQ RHLQNIFVFL PNSTYETTS-
PpaPPO6      D--ILDITPL TFK-VRR--P ---------- -----ARSSV ---------G TEVLE----- --------IS GLQIPNI-TL QAHWKAFLFF PQVNFT-HG-
PpaPPO8      E--TLFVTPI TFR-LKR--P ---------- ------KRRS --------EG TEVLE----- --------IS GLFIPNI-TD QVHVKAYVFF PQAVIT-DG-
PpaPPO3      M--VLHTVPL TFK-VQR--P ---------- -----RRRPE --------VG VEVLE----- --------LS GLSLIP--AL QAHWEAFLFL PNGNNK-TT-
PpaPPO5      D--KLGAEPL TFR-VPR--P ---------- -----ERSDV ---------G VEVLE----- --------IE GIKVDG--RF QSHWEAYLFF PSVDIN-TP-
PpaPPO13     G--TLDAKPL TFR-VTR--P ---------- -----ERSDV ---------G VEVLE----- --------IQ GIKVDN--TL QSHWGAYLFF PSAELN-TS-
BdaPPO5      ---VLKKGRM EYLPVER--P ---------- -----KKDGT --------KT DEVLV----- --------VD -VTLDP--CE QVKFDVLVNV PRGEEDKVG-
BdaPPO4      ---ALKNGRK EYLTVKR--P ---------- -----EKNGG --SKKAP--- -EVLV----- --------ID -VDIDP--CE YAKFDVLVNV PRGQEGKVS-
SitPPO3      A--LTEGQVV EVPSVAK--P R--------- -----RAQKA AAGGGGKQPA DTILV----- --------FD GVEFEP--GK GGKFDVVINV PPEQAAGAG-
SbiPPO6      GGRAVGGDPG EAA-REG--P G--------- -----AAHRG --HRVRPADQ QQVRRDHQRA QGGRRQGGAQ GLRVRRQLHR RAELQRRRRD ARGEGHALH-
ZmaPPO4      ---TLTDEAV DVPAVAV--P A--------- -----RQAGK ---------- DLVLL----- --------IE GIEYDP--QI NNKFDVVINV AREDAARVG-
SbiPPO5      A--LTKGQVV EVPAVAV--P ---------- -----AREAG ---------K EQLLV----- --------ID GIEFDP--QA NNKFDVAINL PADKALQVG-
ZmaPPO5      ---------- ---------- ---------- -----ALTKG ---------- -QVVE----- --------VP AVPVPA---- ---------- -------QG-
SbiPPO7      A--LTKGQVV EVPAVAV--P ---------- -----AREAG ---------K EQLLV----- --------ID GIEFDP--QA NNKFDVAINV PADKASQVG-
A. marina    V--LSFEANV DLS-LRT--R ---------- -----RLIST --AAVGVPNY VVRLR----- --------IE GVKTPK--QQ NTGVFVFLGP -DITAE-TP-
```

```
                    910        920        930        940        950        960        970        980        990       1000
             ....|....| ....|....| ....|....| ....|....| ....|....| ....|....| ....|....| ....|....| ....|....| ....|....|
BdaPPO1      AGGRELAGTF VSM---KQP- SMDHR----T GKRKPMKTSM RVALNELLED LGAD-GDESV TVTLV-PRRG N--------- --VRIGG--- LRIVYMTE--
BdaPPO3      PGGREMAGSF VCL---MHP- SMDG-----T GKGMGIQTSM RVALNELLED LGAD-GDDSV TVTLV-PRNG K--------- --VSIGG--- LRIVYMME--
SbiPPO1      SGGREMAGSF VTL---KHP- ---------G KEGTALRTRM TVALNELLED LGAE-GDDSV TVTLV-PVTG Q--------- --VTIGG--- LRIVYMDE--
OsaPPO1      PCGREMVGSF VCL---RHH- NTQN-----N VTRRGVQTTM RVALNDILKD LGAE-QDESV TVTLV-PRHG K--------- --VRIGG--- VRIEYNGM--
ZmaPPO1      PGGREMAGSF VSL---KHP- ---------- -GNMVVQSSM RVALNELLED LGAE-GDDSV TVTLV-PVEG R--------- --VRIGG--- LRIVYMAE--
SitPPO1      PGAREMAGSF VSL---KQP- RME------A AVGEVASTSM RVALDELLED LGAD-GDDSV TVTLV-PVAG R--------- --VRIGG--- LRIVYMVE--
SbiPPO8      PGGREMAGSF VSL---KHP- ---------- -GEVVVQTSM RVALKEILED LGAE-GDDSV TVTLV-PVEG R--------- --VRIGG--- LRIVYMTE--
ZmaPPO2      TAAAECAGSF VLT---PHVI QQKEE----G GGGSPVKTAA RFGITDLLDD IGAD-GDVTI VVSLV-PRCA GDV------- --VTVDG--- VSVTYVK---
OsaPPO2      TCAATCAGSV ALA---PHG- IHR------E GQLSPRKTEA RFGICDLLDD IGAD-GDKTI VVSIV-PRCG CDS------- --VTVAG--- VSIGYAK---
BdaPPO6      MGAAYCAGSV ALT---PHA- EKR------K EKGAMMKTVA RFGVCDLLEN IGAD-GDEMV VVSLV-PRCG --GEL----- --VTIGG--- VSIGYAK---
SbiPPO3      AAVAQCAGSV AMT---PHL- VRP------G KGRGSVKTAA RFGICDLLDN IGAD-GDKTI VVSLV-PRCA GDM------- --VTVGG--- VRIEYVK---
ZmaPPO6      PAAAQCAGSV ALT---PHA- VRP------G KGKGTVKTVA RFGICDLLDD IGAD-GDKTI IVSLV-PRCA GDM------- --VTVGG--- VGIEYVG---
SbiPPO4      AAAAQFAGSV AMT---PHL- VRP------D KRRGSVKTAA RFGICDLLDD IGAD-GDKTI VVSLV-PRCA GDM------- --VTVGC--- VRIEYIK---
MguPPO1      EENTEFAGSF VNV---PHK- ---------H KHGKKIKTQL RLSITDILED LDAE-DDEHV LVTLV-PTNA GDS------- --ITIHG--- VKIELDD---
BdaPPO2      SGGRELAGSF VSV---PHRH RH-------D KKEKKIKTKL RLALNEQLED LDAE-GDESV VVTLV-PRQG KGK------- --VKIGS--- VKIELID---
SitPPO2      SGGRELAGSF VNV---PHR- HAHGHGR--G KKGGGIKTTL RLALNEQLED LEAE-DDESV EVTLV-PRQG RGK------- --VKVGS--- VRIELVR---
SbiPPO2      SGGRELAGSF VNV---PHGH GHRHGH---G KKGRGIKTKL RLALNXAARG PQGRRRRERG GHPRAAPRQG ---------- ---------- ----------
ZmaPPO3      SGGRELAGSF VNV---PHRH GHGHGH---A KKGRGIKTKL RLALNEQLED LEAE-GDESV VVTLV-PRQG KGR------- --VKIGG--- VKIELMH---
AcoPPO6      PDLGELAGSF VRV---THT- HH-------K KDKVETTSKI ELGVTNLLDD IEAE-ASDSL VVSLV-PQHG D--------- --VTIGG--- VRIDLFASDI
AcoPPO7      PDLGELAGSF VRV---THT- HH-------K KDKVETTSKI ELGVTNLLDD IEAE-ASDSL VVSLV-PQHG D--------- --VTIGG--- VRIDLFASDI
AcoPPO1      ADLGEFAGSF VHI---PH-- ---------A HGPSDHRNIL HLGITYLLED IADAEASEQL VVSIV-PRSG E--------- --VTLTG--- VSIQLVPKEG
VviPPO1      PKNSEFAGSF VNV---PHK- ---------H MKEMKTKTNL RFAINELLED LGAE-DDESV IVTIV-PRAG GDD------- --VTIGG--- IEIEFVSD--
VviPPO3      PRNSEFAGSF VNV---PHK- ---------H MKEMKTKTNL RLAINELLED LGAE-DDESV IVTIV-PRAG GDD------- --VTIGG--- IEIEFVSD--
AcoPPO5      PAQSEFAGSF VNV---PHK- H--------G MKMKKLNTCL RLGIADLLEE IDAE-DDDEI VVTLV-PRQG KD-------- --VTIQG--- IKIVVAS---
VviPPO2      PDNTEFAGSF VNV---PHQ- ---------H SHGKKKNTIL RIGISELLED LEAE-DDDSV VVTLV-PRYG ADV------- --ITIGG--- IEIELAS---
PtrPPO3      PDKTEFAGSF VNV---PHK- ---------H KHGKKMTTCF RLALTDLLED LDVE-GDDSL IVTLV-PRYG KGL------- --AKIGG--- IKIEFDQD--
MesPPO1      PENSEFAGSF VNV---PHA- ---------H KHGKKMKTCL RLGITDLLED LGAE-DDDSV VVTLV-PKCG NGL------- --VKIGG--- IKIDFTQD--
RcoPPO1      PDNSEFAGSF VNI---PHK- ---------H KHGKKMKTCL RLGLTDLLED MGAE-DDDTV VVTLV-PRYG KGL------- --VKIGG--- IMIDYIQD--
PtrPPO11     ADDAEFAGTF VNV---PHN- ---------- -RNKTVKTSL RLGISELLED LEAE-DDESV VVTLV-PVTN IGE------- --ATIGT--- LRIELLKD--
PtrPPO1      PENTEFAGSF VNV---SHK- ---------- -HAKKSKTRL ILGITELLED LESD-GDDSI VVALV-PRSN SVSDP----- --VVISG--- VKIEFVKE--
PtrPPO12     PDKSEFAGSF VNV---PHK- ---------- -HAKKSKTTM VLGITGLLED LEAE-GDDTL VVTLV-PRTG GDS------- --VTVAN--- VKIEFVAD--
PtrPPO5      PDMSEFAGSF VNV---PHK- ---------- -HAKKSKTTM VLGITGLLED LEAE-GDDTL VVTFV-PRTG GDS------- --VTVAN--- VKIEFVAD--
PtrPPO14     PDNSEFAGSF VNV---PHK- ---------- -HAKKSKTTM VLGITGLLED LEAE-GDDTL VVTLV-PRSG GDS------- --VTVAN--- VKIEFVAD--
PtrPPO2      PDKSEFAGSF INV---PHK- ---------- -HAKKSKTTM VLGITGLLED LEAE-GDDTL VVTLV-PRTG GDS------- --VTVAN--- VKIEFVAD--
PtrPPO9      PDKSEFAGSF VNV---PHK- ---------- -HAKKSKTTM VLGITGLLED LEAE-GDDTL VVTFV-PRSG GDF------- --VTVAN--- VKIEFVAD--
PtrPPO15     PDKSEFAGSF VNV---PHK- ---------- -HAKKSKTTM VLGITGLLED LEAE-GDDTL VVTLV-PRTG GDF------- --VTVAN--- VKIEFVAD--
GmaPPO2      PQDSEFAGSF VTL---PHSP H--------V NHNNITKASF KLPLTYKLKD LGVTKDDDSI SVTLA-PIYG DKP------- --VTIKD--- VRIKRVYPEV
GmaPPO4      ---------- ------QHS- ---------- -RHYSSLSFW N--------- ---------- ------P--- ---------- --LSVQT--- ----------
GmaPPO3      PEDSEFAGSF MTL---AHS- H--------G HQSKRTTTSL RLAITDLLKD LHAL-DDESI AVTLA-PRYG NKP------- --VTIKG--- IKIKLVPLVE
GmaPPO5      PENTEFAGSF ATL---GHS- HSN------M NMDKKIKTSL TLGITDLLED LDAE-NDDSI LVTLV-PRSE NVT------- --ITIQS--- IKIEFEKEE-
GmaPPO6      PESTEFAGSF STL---GHS- ---------- HSNMNMDEKI KTSL------ ---------- ---------- ---------- ---------- ----------
GmaPPO8      PESTEFAGSF STL---GHS- HSN------M NMDKKIKTSL TLGITDLLED L----DADNA IVFCI----- --IDD----- --KEIND--- SRKN------
GmaPPO7      PSNTEFAGSF VSV---PHS- H--------V HKNKKIKTCF RVGLTDLLED LEAE-NDDSI LVTLV-PKYG KGL------- --VTIRD--- IKIELETE--
GmaPPO1      PEDSEYAGSF VTV---PHS- H--------K HKNKKIITCL RLGLTDLLEE LEAE-DDDSV VVTLV-PRYG KGR------- --VQIGG--- IKIDLVAD--
GmaPPO9      PDNTEFAGSF VSV---PHS- H--------K HKNKDIITCL RLGLTDLLEE LEAE-DDDSV RVTLV-PRYG KGR------- --VKIRG--- IKIKLLAD--
GmaPPO10     PDNTEFAGSF VSV---PHS- H--------M HKNKDIITCL RLGLTDLLEE LEAE-DDDSV RVTLV-PRYG KGR------- --VKIRG--- IKIELLSD--
PpaPPO4      TADLSFVAIY NNL---PLR- SI-------D EEQKQDKVTF SIAMGMKLRS LELR-KRTCI PISLV-PVPG TNGNAHM--- --ISVSN--- IYIKSVIP--
SmoPPO6      LSCAEYVGSF YNI---PHF- MPG------M TESSTRTTNA RFSIKPNVEI LGLK-DADKL VVTLV-PRGK DKEKV----- --FTFKG--- ASIQYA----
SmoPPO5      LSCAEYVGSF YNI---PHF- MPG------M TESSTRTTNA RFSIKPNVEI LGLK-DADKL VVTLV-PRGK DKEKV----- --FTFKG--- ASIQYA----
SmoPPO7      LSCAEYVGSF YNI---PHF- MPG------M TESSTRTTNA RFSIKPNVEI LGLK-DADKL VVTLV-PRGK DKEKV----- --FTFKG--- ASIEYA----
SmoPPO8      LSCAEYVGSF YNI---PHF- MPG------M TESSTRTTNA SFSIKPNVEI LGLK-DADKL VVTLV-PRGK DKEKV----- --FTFKG--- ARIQYA----
SmoPPO9      VSCAEYVGTY SNV---PHFM L--------G IGEHTETVNT TFSIKANVEI LGLK-SADKL VVTLV-PRGQ GT-------- --FTLKG--- ASIQYA----
SmoPPO4      VSCAEYVGTY SNV---PHFM LE-------S GKWHTETVNT SFSIKANVEI LGLK-SEDKL VVTLV-PRGD GS-------- --FTFNG--- ASIQYA----
SmoPPO11     VSCAEYVGSY SNV---PHF- IL-------K SGEHTEIVNI TFSIPDRLAS HSRGLPFSML DFDFF----- ---------- --LVINK--- ----------
SmoPPO10     VSCAEYVGTY SNV---PHF- MSD------I GKGHTEIVNA TFSIKANVEI LGLK-SADKL VVTLV-PRGD --GS------ --LTFRG--- ASIQYA----
MguPPO2      LDKAEYAGSY SQV---PHR- ---------- -NSTTVKTRI RLGLTELLED LDVE-GDDKI LVTLV-PKAG GED------- --ITIGG--- INIIYADDD-
MguPPO5      RDKAEFAGAY SQV---PHK- ---------- -NSTRTKSKI RLGLTELLED LDVD-DDDQI LVALV-PKAG GED------- --ITIGG--- IKIIYAS---
MguPPO6      LDKAEYAGTF AQV---PHK- ---------- TKDKKGKSTI SLRLTELYED VDVA-DDDTV VVTLI-PRSN GDD------- --VTIGG--- IKIIASPPRS
MguPPO3      LDKAAYAGTY AQV---PHK- ---------- TANKTATTSI RLKLTELYDD MDVG-DDDTI VVTLV-PRHQ GPGEAASFLY FIFSLIG--- LQCYVLCLLI
MguPPO4      LDKAAYAGTY AQV---PH-- ---------K TNEKTATTTI RLKLTELYDD MDVT-EDDTV LVTLV-PRHE VPR------- --LNLNGTVP LKWKYVIAHI
MguPPO7      LDKASYLGTY AQL---PHK- --------PR SGTTSNSSSI RLKLTDLYDD IDIG-DDDAI VVTLV-PRHQ GPG------- --VTIGG--- IKIIENPTKP
MguPPO8      LDKAAYLGTY AQV---PHK- ---------S ANNNSSTSSI KLKLTELYDD MDID-DDDTI VVTLV-PRHQ GPVLP----- --LVVSR--- LSRIHRNRLP
MguPPO9      IDKASYAGTY AQI---PH-- ---------K TKEKTATTSI RLKLTDVYED MDVT-DDDTI LVTLV-PRHE GPG------- --VTIGG--- IKIIENPTPA
GmaPPO11     PDQTEFVGTF VNL---FHG- ---------- -QGHNINTSF KVGISKVLEC LEAE-EDDVV LVTLV-PKVG KGD------- --VIIGG--- IKIEFIPK--
SmoPPO2      VNVVEFAGTF VNV---PNS- ---------A GKGHIMETEV RLGISDTLEQ LGLE-DADYI VVTLV-PRGS SGP------- --VTFKG--- FKIKYET---
SmoPPO3      VNVVEFAGTF VNV---PDS- ---------A GKGHIMETEM RLGISDTLEQ LGLE-DADYI VVTLV-PRGS SGP------- --VTFKG--- FKIKYET---
SmoPPO1      CNVAEYAGTF VNV---PHF- GDRR-----G GPGHMRTTDL RLGISDTLDQ LGIK-DARNI VVTLV-PRGV GRLDP----- --IKFKG--- FKIEYE----
PtrPPO13     PRFREFAGTF VHI---DPG- VTRVARES-N IEVFRKKTDL KLGISELLED LEAE-GDENI WVTLL-PRSE GCIN------ --TTVDG--- LRIEYIR---
VviPPO4      PEFREFAGTF VNI---PIG- ---------V WPVMRRKSNL KLGISELLQD LEAD-EDESI WVTLI-PRAE DCMN------ --VTIDG--- IRIEYIE---
AmePPO1      PESREFAGTF VNM---KRG- VRLIMNK--E DLIVKRKTVL KLGISELLED LEAE-GGESI WVTLV-PRGG TGIN------ --TTIDG--- VRIEYMR---
AcoPPO4      PESREFAGTY VNL---PRG- VTRVLND--G DVETKSKSTL KLGISELLDD LQAN-EEDSI WVTLV-PRGG TGVH------ --TSVEG--- IRIEYMA---
AcoPPO2      PESTEFAGTY VSL---PKG- ITLVLNE--G EVKRKSKSTL KLGISELLDG LQAN-EEESL WVTLV-PRGG TGVH------ --TSVDG--- IRIEYMA---
AcoPPO3      PESTEFAGTY ISL---PHG- RTLLLNQ--G DVKTKSKSTL KLGISELLND LQAN-EEESI WVTLV-PRGR TGVH------ --TRVDG--- IQIEYIA---
PpaPPO9      IDCMEFFGTF AHI---PAS- N--------H KKKLGYSRSW RLGLTTKLAQ LGKS-GFSQV VVTLV-QVGS NQH------- --IKFGA--- TKIKLEK---
PpaPPO12     IDCMEFFSTF AHI---PEA- H--------H RKKKGYTRSW KLGLTEKLVQ LGLS-QVSHA VITLV-QVGS DQQ------- --IKFGS--- ARILLED---
PpaPPO2      ISCPEFFGTF SHI---PEA- A--------R TRPKDFSMTW TLGLTTRLRE IGKL-HVRHV VVTLV-QIGS EQL------- --IRFQD--- AIIEIDSS--
PpaPPO10     TGCHEFFGTF SHI---PQA- D--------H HRPSDFSMQW TLGLTSKLQD IRKP-YVSHV VVTLV-QVES NQQ------- --IKFKG--- ITMEIDRKW-
PpaPPO1      IGCLEYIGNL LSF---PHV- GQA------P AVPPVVKVYW KFALLP---- ---------- ------PRRS SAG------- --CSSSG--- ----------
PpaPPO7      VGCFEYIGNL LSF---PHT- G--------M SPGIKPVIRF RMGIQRKLEL MSRA-HLKEV VITLVGEAPV ASTDDTGETG --FNLTS--- LTIIYSKE--
PpaPPO11     IGCLEFIGNM FSF---PHP- G--------M SPDLQPELTF RFGIRRKLEV MGRA-NLTEI VITMV---GE NAAPPFIAGE AGFNLTS--- AKIVYSKN--
PpaPPO6      AICPEFVGTY NFI---PHV- ---------G QAKYNPKRVW RVAVGPKLKA LGLD-HVKSV IVTLVQPPGP WLQD------ --IKFTK--- AKILYDTSG-
PpaPPO8      PVCPEFLGTF NFI---PHI- ---------G QALYNPGRVW RAAVGPKLKA LGLD-YVNHV VFTIV-QTGK --PQM----- --LSFQK--- ARIIYDLSPE
PpaPPO3      VSCPEFIGTF NYV---AHV- ---------G QALFNRERRW RVALGPKIMA LGKE-NYTDV VITLA--QFG PNIQP----- --ITFER--- AVVVFDTDGV
PpaPPO5      VSCPEFFGTF NFV---PHV- ---------G QAQFNRDRVW RVAVKDKLVA LGKD-NYTDI IVTLV--QFG PNVQP----- --LGIGK--- ARIVYDASPA
PpaPPO13     VSCPEFFGTF NFS---PHV- ---------G QAQVTRDLVW RVGIRQKLID LGKD-DYDDI VVTLV--RFG PSIQQ----- --LQLGG--- TQVLYDTSPT
BdaPPO5      PQNSQFAGSF STV---PHG- GTMT-----G SATTKPVVSC RFKLQELIQD LNCN-RSKML NITLV-PVEG DK-------- --TIVDN--- LRVELC----
BdaPPO4      PQNSEFAGSF VNL---PHG- GGDGGM---K VGMGRLKLAY RFALRELIED LRCG-GDATL DVTLV-PVAG EM-------- --VVVDG--- VRVELLK---
SitPPO3      PRHSEYAGSF ATL---PRG- GS-------K KPGETVVVPF VLPLDEVLAD IGVGDEDGAV NVVIV-PRTP G--------- --IKIIS--P PRIEIRER--
SbiPPO6      RRGAGRPRAH QRE---RRR- ---------H RAGSSHRR-- ---------- -----GDQDL ------PAAD HRERM----- ---------- ----------
ZmaPPO4      PKDSEYAGSF SAV---PSS- ---------N AAGGTLVGKF TLALDGVLAD LGLA-GASAV DIVLV-PHTE --GEI----- --KLYLP--- PTIENA----
SbiPPO5      PQYKEYAGSF AVV---PGS- ---------- GGGETRIGKV TLCITDVLYD LDAE-DDSSV DVVIV-PRTD AK-------- --VTINV--- RPIIKNRN--
ZmaPPO5      PGEGAAAGDR GHRVRPP--- ---------- ---------- ---------- -----GEQ-- ---------- ---------- ---------- ----------
SbiPPO7      PQYKEYAGSF AVV---PG-- ---------S GGGGTRKGKV TLCITDVLYD LDAE-DDSSV DVVIV-PRTD AK-------- --VTINA--- RPTIKNRN--
A. marina    ISAPGYVGNF TFF---EGQ- GGGNRKRWSH GHKKNVLLNA TEAVKRLYGD TRLS-ESNSL KVSIV-TRSL FGDQNAFASV EEIQPNM--- VQIDVVDLNA
```
